# Brain MRI changes in degenerative cervical myelopathy: a systematic review

**DOI:** 10.1016/j.ebiom.2023.104915

**Published:** 2023-12-19

**Authors:** Amir Rafati Fard, Oliver D. Mowforth, Melissa Yuan, Samuel Myrtle, Keng Siang Lee, Arka Banerjee, Maaz Khan, Mark R. Kotter, Virginia F.J. Newcombe, Emmanuel A. Stamatakis, Benjamin M. Davies

**Affiliations:** a School of Clinical Medicine, University of Cambridge, Cambridge, UK; b Division of Academic Neurosurgery, Department of Clinical Neurosciences, Addenbrooke's Hospital, University of Cambridge, Cambridge, UK; c Department of Neurosurgery, King's College Hospital, London, UK; d PACE Section, Department of Medicine, Addenbrooke's Hospital, University of Cambridge, Cambridge, UK

**Keywords:** Cervical, Myelopathy, Magnetic Resonance Imaging, Structural, Functional

## Abstract

**Background:**

Degenerative cervical myelopathy (DCM) is the most common cause of adult spinal cord dysfunction globally. Associated neurological symptoms and signs have historically been explained by pathobiology within the cervical spine. However, recent advances in imaging have shed light on numerous brain changes in patients with DCM, and it is hypothesised that these changes contribute to DCM pathogenesis. The aetiology, significance, and distribution of these supraspinal changes is currently unknown. The objective was therefore to synthesise all current evidence on brain changes in DCM.

**Methods:**

A systematic review was performed. Cross-sectional and longitudinal studies with magnetic resonance imaging on a cohort of patients with DCM were eligible. PRISMA guidelines were followed. MEDLINE and Embase were searched to 28th August 2023. Duplicate title/abstract screening, data extraction and risk of bias assessments were conducted. A qualitative synthesis of the literature is presented as per the Synthesis Without Meta-Analysis (SWiM) reporting guideline. The review was registered with PROSPERO (ID: CRD42022298538).

**Findings:**

Of the 2014 studies that were screened, 47 studies were identified that used MRI to investigate brain changes in DCM. In total, 1500 patients with DCM were included in the synthesis, with a mean age of 53 years. Brain alterations on MRI were associated with DCM both before and after surgery, particularly within the sensorimotor network, visual network, default mode network, thalamus and cerebellum. Associations were commonly reported between brain MRI alterations and clinical measures, particularly the Japanese orthopaedic association (JOA) score. Risk of bias of included studies was low to moderate.

**Interpretation:**

The rapidly expanding literature provides mounting evidence for brain changes in DCM. We have identified key structures and pathways that are altered, although there remains uncertainty regarding the directionality and clinical significance of these changes. Future studies with greater sample sizes, more detailed phenotyping and longer follow-up are now needed.

**Funding:**

ODM is supported by an Academic Clinical Fellowship at the University of Cambridge. BMD is supported by an 10.13039/501100000272NIHR Clinical Doctoral Fellowship at the University of Cambridge (NIHR300696). VFJN is supported by an 10.13039/501100000272NIHR Rosetrees Trust Advanced Fellowship (NIHR302544). This project was supported by an award from the Rosetrees Foundation with the Storygate Trust (A2844).

**Research in context**

**Evidence before this study**

There has been increasing evidence to show that chronic injury to the spinal cord, as occurs in degenerative cervical myelopathy (DCM), not only impacts function at the spinal level, but also upstream structures within the brain. Previous studies have utilised magnetic resonance imaging (MRI) to investigate key structures and pathways within the brain that may contribute to DCM pathology. There has been no previous review to aggregate these findings. To this end, we searched MEDLINE and Embase for cross-sectional and longitudinal studies published until 28th August 2023 that utilised MRI to assess brain changes in a cohort of patients with DCM.

**Added value of this study**

In this systematic review of brain MRI changes in DCM, we show that structures and pathways within the brain are altered on MRI compared to healthy controls. Moreover, their response to treatment and relationships to clinical measures suggest that these brain changes are related to the disease biology. Whilst risk of bias of included studies was low to moderate, we identify several significant sources of potential bias, and suggest how they can be reduced in future studies.

**Implications of all the available evidence**

MRI provides researchers an elegant means to probe brain changes that occur in DCM, but to date, studies have been heterogenous, both in terms of methodological approaches (e.g., MRI sequence, type of analysis) and results. Despite this, in keeping with the wider evidence, we suggest that this confirms imaging of the brain in DCM as an important area for future research, offering potential dividends for diagnosis, prognostication and stratification, relevant to both research and the clinical care of patients with DCM. Future studies with larger sample sizes, more detailed phenotyping and longer follow-up are now required to provide more certainty to the current evidence base.

## Introduction

Degenerative cervical myelopathy (DCM) describes degenerative changes in the cervical spine that lead to symptomatic cervical spinal cord compression.^1^ DCM is common and can manifest as a wide range of disabling symptoms, including limb dysfunction, sensory abnormalities, gait disturbance and bladder dysfunction.^2^ In severe cases, DCM can progress to paralysis. Timely surgical decompression is currently the only disease modifying treatment.^3^ DCM severely impacts quality of life,^4^ and alongside an increasing prevalence due to population ageing, necessitates a global research effort to better inform diagnosis, stratification, and prognostication.

Whilst most research to date in DCM has focussed on the structural and functional abnormalities of the cervical spinal cord, emerging evidence has shown that chronic spinal cord compression can secondarily impact brain structure and functional networks.^5^ The ‘intrinsic brain networks’ paradigm suggests that spatially remote regions of the brain collaborate in certain functions,^6^ with core networks including the default mode network (DMN), sensorimotor network (SMN) and visual network (VN). This paradigm has been recently extended to encapsulate the spinal cord in an ‘intrinsic central nervous system network’,^7^ reinforcing the notion that a spinal condition, such as DCM, may affect upstream brain networks. In support of this, patients with even mild DCM have been shown to have brain changes on MRI.^8^

Conceptually, neuroimaging using MRI can be divided into structural, functional and ‘other’ techniques (Table 1). Structural techniques analyse anatomical information enabling investigation of geometric properties, such as grey matter volume (GMV).^9^ Diffusion-weighted MRI (DWI) provides an assessment of fractional anisotropy (FA), apparent diffusion coefficients (ADCs) and structural connectivity (SC). In contrast, functional techniques focus on the spatiotemporal organisation of brain activity, such as functional connectivity (FC) or volume of activation (VOA), and underlying brain function, e.g., by use of graph theory analysis, associated with a particular task or during resting state.^10^ fMRI measures the blood oxygen level dependent (BOLD) signal; this can be further analysed to measure regional homogeneity (ReHo) and amplitude of low-frequency fluctuation (ALFF), which each provide insight into different aspects of regional neural activity. Other MRI techniques (separate to structural and functional), include arterial spin labelling (ASL) and MR spectroscopy.

**Table 1:** Summary of brain MRI outcomes measured in DCM neuroimaging research.

MRI outcome	Description
**Grey Matter Volume (GMV)/White Matter Volume (WMV)**	Structural MRI measure that assesses the volume of grey matter or white matter in specified brain structures.
**Fractional Anisotropy (FA)**	Structural MRI measure that assesses the directionality of water diffusion as a marker of axonal diameter and fibre density in white matter tracts. **Generalised Fractional Anisotropy** (**GFA**) and **Normalized Quantitative Anisotropy** (**NQA**) represent more advanced measures of anisotropy.
**Apparent Diffusion Coefficient (ADC)**	Structural MRI measure that assesses the rate of water diffusion as a marker of histological architecture. **Mean Diffusivity** (**MD**) similarly assesses the molecular diffusion rate of water as a marker of tissue integrity.
**Structural Connectivity (SC)**	Structural MRI measure that assesses the anatomical organisation of fibre tracts in the brain.
**Functional Connectivity (FC)**	Functional MRI measure that assesses the temporal correlation of BOLD signal changes between regions of the brain as a marker of cooperativity. **Functional Connectivity Strength** (**FCS**) is a marker of voxel-level degree centrality. **Global Functional Connectivity Density** (**gFCD**) is a marker of whole-brain FC patterns at the voxel level. **Effectivity Connectivity** (**EC**) is a marker of the directional connection across brain regions.
**Volume of Activation (VOA)**	Functional MRI measure that assesses the spatial extent of brain regions involved in a specific task.
**Amplitude of Low-Frequency Fluctuation (ALFF)**	Functional MRI measure that assesses spontaneous fluctuations in BOLD signal intensity as a marker of regional neuronal activity. **Static Amplitude of Low-Frequency Fluctuation** (**sALFF**) is a marker of local neuronal activity. **Dynamic Amplitude of Low-Frequency Fluctuation** (**dALFF**) is a marker of the temporal variability of intrinsic brain activity.
**Graph Theory**	Functional MRI measure that assesses brain networks by modelling brain regions as nodes and their connections as edges.
**Blood Oxygen Level Dependent (BOLD) Signal**	Functional MRI measure that assesses changes in blood oxygenation as a marker of neuronal activity.
**Regional Homogeneity (ReHo)**	Functional MRI measure that assesses the synchronisation between the time series of a given voxel and its nearest neighbour as a marker of regional neuronal activity.
**Metabolites**	‘Other’ MRI measure that assesses metabolite (e.g., N-acetylaspartate) concentrations as a marker of metabolism and neurochemistry.
**Cerebral Blood Flow (CBF)**	‘Other’ MRI measure that assesses the perfusion to different regions of the brain as a marker of activity.

Importantly, the implications of employing MR imaging to better understand brain changes in DCM are of significance to both the research and clinical community. Firstly, whilst structural MRI of the cervical spine is conventionally regarded as the gold-standard diagnostic and prognostic imaging modality for DCM, its utility as a prognostic tool has been limited to date. MR imaging of the brain, therefore, has potential to offer different prognostic insights. This is the experience from traumatic spinal cord injury (SCI), where various cerebral biomarkers have been recommended for trials.^11^ Practically, imaging the brain is also attractive, since it is less prone to artefacts, as cervical spine MRI is susceptible to the surgical hardware frequently used to treat DCM (e.g., implants), and easier to analyse, as spinal cord anatomical deformity may cause challenges to automated segmentation and labelling algorithms that are critical for consistent analysis. Secondly, and perhaps more importantly, investigating brain changes associated with DCM may shed light on key mechanisms underlying its pathogenesis (such as neuroplasticity). DCM is now proposed to be a function of mechanical stress, time and vulnerability to acquire injury and symptoms.^12^ Therefore, as a chronic disease, how the central nervous system adapts to spinal cord injury is likely relevant. Brain MRIs may also help to explain the increasing description of symptoms that are classically unrelated to a disease of the spinal cord (e.g., visual loss, face numbness) and the reported differences in surgical outcomes between patients.^13^

Our objective was to provide an overview of the expanding literature on brain changes observed in patients with DCM using MRI, to characterise the current methodological approaches and findings, and through aggregate analysis consider key regions, structures and pathways that change in the brain that may relate specifically to DCM.

## Methods

This systematic review was reported in accordance with the Preferred Reporting Items for Systematic Reviews and Meta-Analysis (PRISMA) guidelines (Supplementary Data 1).^14^ The protocol was registered on PROSPERO (ID: CRD42022298538).

### Search strategy and selection criteria

The search strategy was developed and iteratively refined, including review by a medical librarian at the University of Cambridge. Published DCM search filters were utilised.^15^^,^^16^ Searches were performed in MEDLINE and Embase using Ovid (Wolters Kluwer, Netherlands) from inception to 28th August 2023 (Supplementary Data 2). Search sensitivity was evaluated using nine papers known to meet inclusion criteria: all papers were successfully captured.

Primary studies reporting on a cohort of patients with DCM (*Population*) before and/or after surgery (*Intervention*) using brain MRIs (*Outcome*) were included. There were no specified comparators for inclusion. Studies were excluded if the title and abstract indicated failure to meet all of the specified inclusion criteria or satisfied at least one of the exclusion criteria (Table 2). Four reviewers (ARF, KSL, AB and MK) independently performed title and abstract screening with blinding using Rayyan (Rayyan Systems Inc., Cambridge, USA). A pilot screen of 100 publications was first conducted to ensure concordance between reviewers. Any disagreements following unblinding were resolved by discussion between the reviewers until mutual agreement was reached.

**Table 2:** Inclusion and exclusion criteria.

Inclusion criteria	Exclusion criteria
Primary study DCM cohort Cross-sectional and longitudinal brain MR imaging	Non-English language Non-human study Mixed cohort unable to have results separated (e.g., cervical spondylosis without myelopathy) Systematic review or meta-analysis Editorial, case report, opinion article, letter, conference abstract, correction Full text unavailable

### Data extraction and critical appraisal

Articles were retrieved for full text screening and data extraction using a piloted proforma. Data extracted from articles included: author, year of publication, study objectives, study design, inclusion/exclusion criteria, sample characteristics (size, mean age, handedness and symptom duration), MRI scanner(s) (brand and strength), MRI sequence(s) collected, MRI outcome(s) assessed, type of surgery and follow-up duration, clinical measures before and after surgery, MRI brain changes before and after surgery, key associations between clinical measures and MRI brain changes, relevant statistical analysis, main conclusions and limitations.

Risk of methodological bias in individual studies was assessed using the Joanna Briggs Institute (JBI) critical assessment tools for analytical cross-sectional studies or cohort studies, depending on study methodology. As advocated by JBI, no study was excluded because of risk of bias. Criteria 3 and 6 were deemed not applicable to the studies included in our review for the analytical cross-sectional and cohort study checklists respectively. Each study was assigned a score from 0 (no criteria satisfied) to 7 or 10 (all criteria satisfied for cross-sectional or cohort studies respectively), where a ‘Yes’ scored 1, ‘Unclear’ scored 0.5 and ‘No’ scored 0. We trichrotomised this ordinal scale to classify the risk of bias as high (scores 0–2 or 0–3 for cross-sectional and cohort studies respectively), moderate (scores 2.5–4.5 or 3.5–6.5 for cross-sectional and cohort studies respectively), and low (scores 5–7 or 7–10 for cross-sectional and cohort studies respectively).

Full text screening, data extraction and risk of methodological bias were assessed independently in duplicate (ARF and MY/SM) with blinding. Any disagreements following unblinding were resolved by discussion between reviewers until mutual agreement was reached. Any outstanding questions were resolved by discussion with the senior reviewer (ODM).

### Data analysis and reporting

Due to significant heterogeneity between methodologies, meta-analysis was not possible and a qualitative synthesis without meta-analysis (SWiM) was conducted (Supplementary Data 1).^17^ Studies were first grouped based on when they assessed brain changes: either only before surgery or both before and after surgery. They were further grouped based on whether they utilised structural, functional or ‘other’ MRI techniques. Finally, studies were grouped based on specific MRI outcomes assessed. The differences (including mean/median/*p* values) between patients with DCM and comparators reported by individual studies were initially summarised in the extraction table. Akin to a harvest plot, brain maps were created to visually summarise the location, direction of effect by colour (increase/decrease/no change) and confidence by colour intensity (number of patients, as a proportion of total sample size).

### Generation of brain maps

Brain maps were generated to provide a visual summary of our results using the BrainPainter software.^18^ In order to do so, studies were first grouped based on the MRI technique used: structural or functional. MR Spectroscopy and ASL (grouped under ‘other' MRI techniques) were deemed too dissimilar, so no brain maps were generated for these MRI techniques. Studies were then further selected based on whether they utilised the specified comparator. For pre-surgical brain maps, the specified comparator against pre-surgical patients with DCM was healthy controls (HCs). For post-surgical brain maps, two separate brain maps were generated for post-surgical patients with DCM with the specified comparators being pre-surgical patients with DCM or HCs respectively. In addition to not meeting the aforementioned criteria, a study was excluded if its analysis was not based on discrete brain regions (e.g., white matter tract or whole-brain network analyses), or if the results were reported for each individual patient with DCM (rather than for the DCM cohort as a whole).

Following selection of appropriate studies for a brain map, regions of brain reported to have significantly changed (increase/decrease) within a study were allocated valence based on the sample size of patients with DCM in that study. The brain regions reported in studies were matched to the most appropriate structure(s) in the Desikan-Kiliany atlas. For example, a study of 10 pre-surgical patients with DCM compared to HCs, which found significantly increased structural MRI changes in the L primary motor cortex, would have been allocated a valence of 10 to L precentral gyrus in a structural pre-DCM versus HCs brain map. Once all the included studies for a brain map were totalled, the final valence for each brain region was divided by the sum of the DCM sample size across all included studies in that brain map. This ensured standardisation of our colour scale across brain maps.

Using the BrainPainter software,^18^ brain maps were generated to show brain regions reported as: (1) significantly increased (white-red colour scale, where increasing intensity of red signifies greater number of patients with DCM reporting increase in that brain region), and (2) significantly decreased (white-blue colour scale, where increasing intensity of blue signifies greater number of patients with DCM reporting decrease in that brain region). Recognising the valence within a region could be conflicting (i.e., both reported to have significant increase and significant decrease), a third brain map was generated, where brain map (2) was subtracted from brain map (1), to provide a ‘net difference’ (blue-white-red colour scale, where increasing intensity of blue signifies that a greater number of patients with DCM reported to have significant decrease for that brain region compared to increase, vice-versa for red). For example, if the L precentral gyrus had a valence of 150 for brain map (1) (increased MRI measures) and a valence of 100 for brain map (2) (decreased MRI measures), then it would score an overall valence of 50 in the direction of increased MRI measures (i.e., red). The following orientations were used in our brain maps: top, cortical-outer-right-hemisphere, cortical-inner-right-hemisphere, cortical-outer-left-hemisphere, cortical-inner-left-hemisphere, and subcortical.

### Role of funders

None.

## Results

**Fig. 1:**
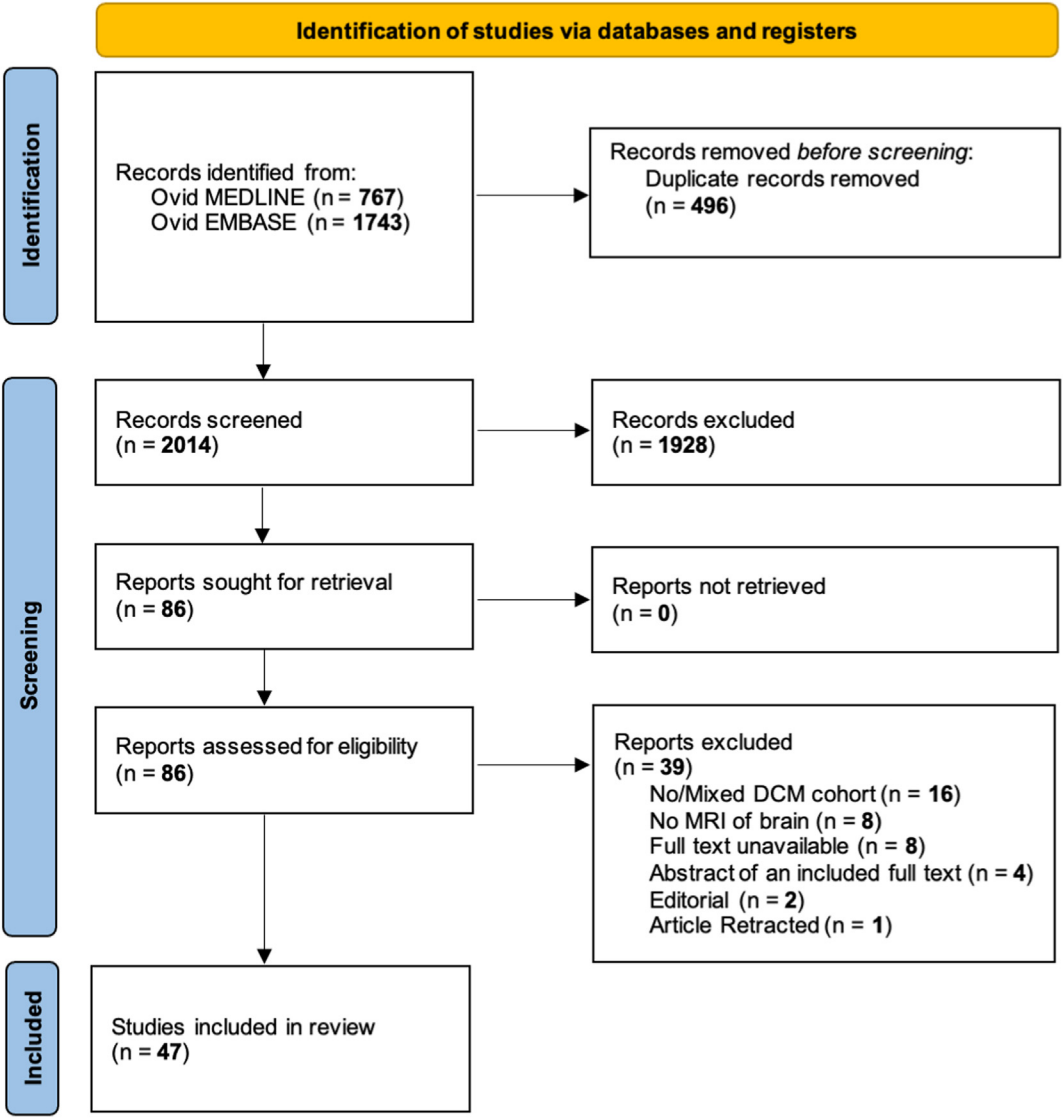
**PRISMA flow diagram of study selection**.

From 2510 results, 47 studies were included in the final analysis (Fig. 1).^19^, ^20^, ^21^, ^22^, ^23^, ^24^, ^25^, ^26^, ^27^, ^28^, ^29^, ^30^, ^31^, ^32^, ^33^, ^34^, ^35^, ^36^, ^37^, ^38^, ^39^, ^40^, ^41^, ^42^, ^43^, ^44^, ^45^, ^46^, ^47^, ^48^, ^49^, ^50^, ^51^, ^52^, ^53^, ^54^, ^55^, ^56^, ^57^, ^58^, ^59^, ^60^, ^61^, ^62^, ^63^, ^64^, ^65^

**Fig. 2:**
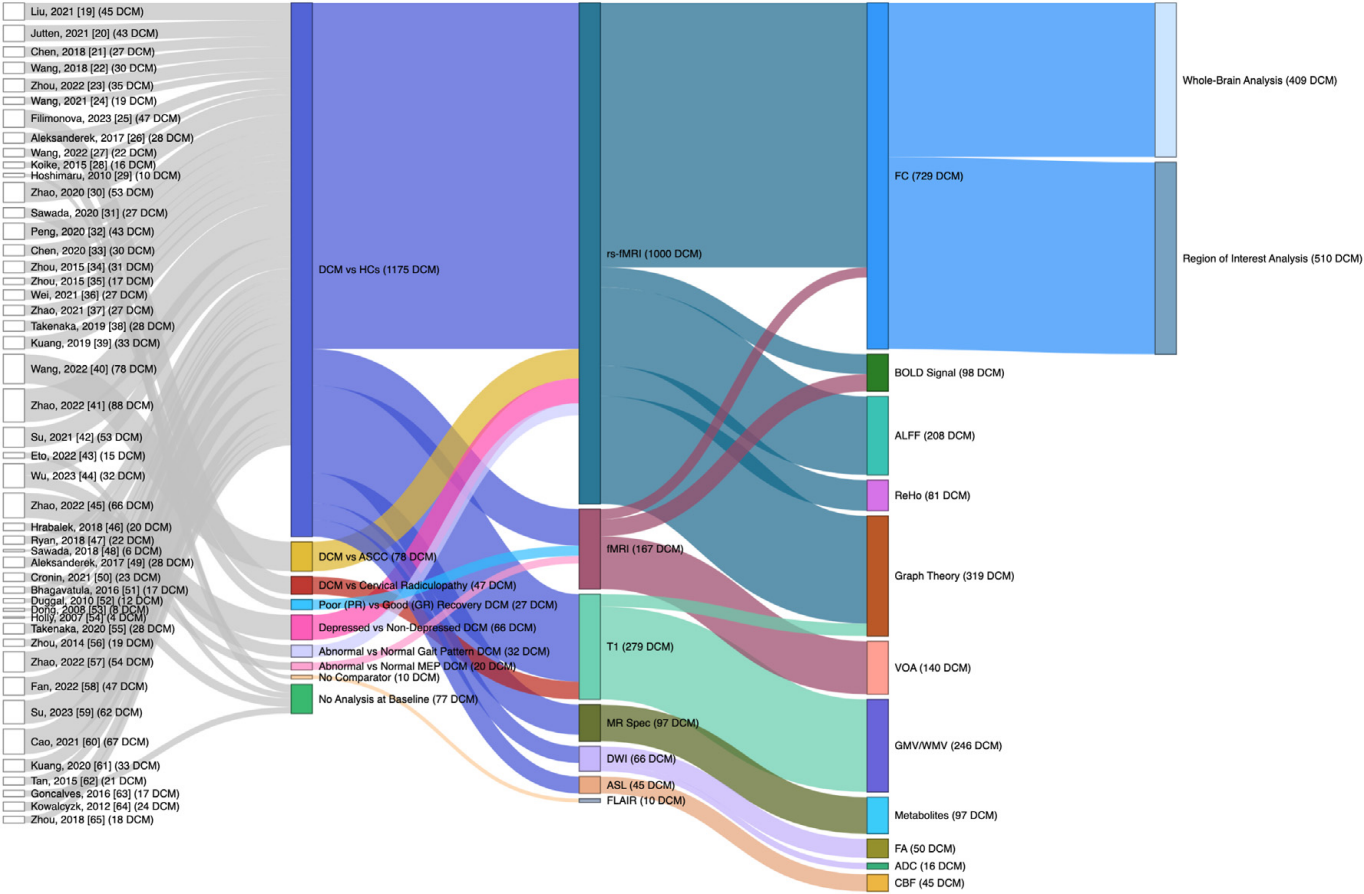
**Study characteristics of 47 studies included at baseline.** Node and link size is proportional to the number of patients with DCM included. Columns from left to right represent study, comparator used, MRI sequence, MRI outcome assessed and type of MRI analysis. ADC = apparent diffusion coefficient; ALFF = amplitude of low-frequency fluctuations; ASCC = asymptomatic spinal cord compression; ASL = arterial spin labelling; BOLD Signal = blood oxygen level dependent signal; CBF = cerebral blood flow; DCM = degenerative cervical myelopathy; DWI = diffusion-weighted MRI; FA = fractional anisotropy; FC = functional connectivity; FLAIR = fluid attenuated inversion recovery; GMV = grey matter volume; HCs = healthy controls; MEP = motor evoked potentials; MR Spec = MR spectroscopy; ReHo = regional homogeneity; rs-fMRI = resting state fMRI; VOA = volume of activation; WMV = white matter volume.

**Fig. 3:**
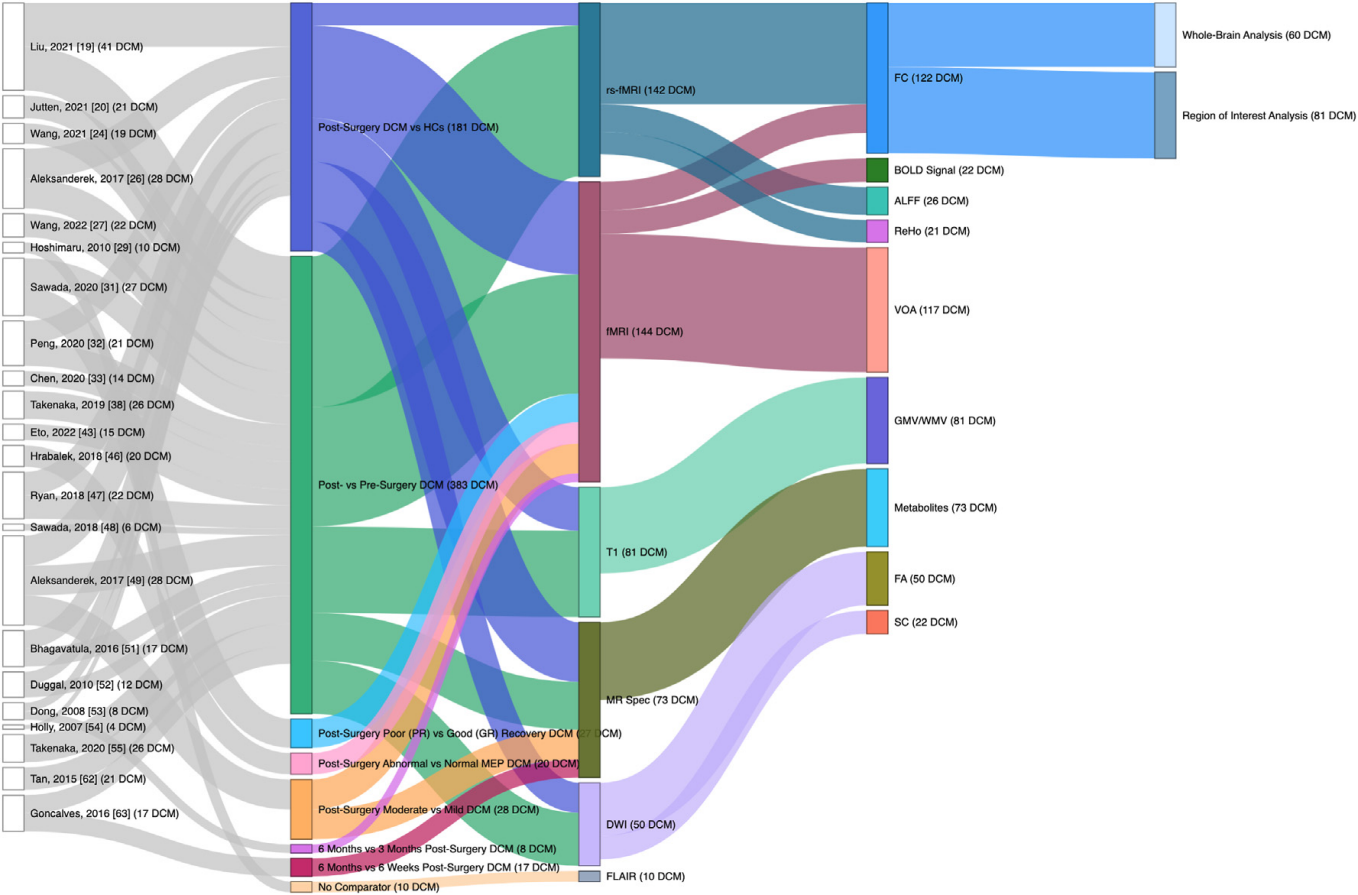
**Study characteristics of 22 studies included post-surgery.** Node and link size is proportional to the number of patients with DCM included. Columns from left to right represent study, comparator used, MRI sequence, MRI outcome assessed and type of MRI analysis. ALFF = amplitude of low-frequency fluctuations; BOLD Signal = blood oxygen level dependent signal; DCM = degenerative cervical myelopathy; DWI = diffusion-weighted MRI; FA = fractional anisotropy; FC = functional connectivity; FLAIR = fluid attenuated inversion recovery; GMV = grey matter volume; HCs = healthy controls; MEP = motor evoked potentials; MR Spec = MR spectroscopy; ReHo = regional homogeneity; rs-fMRI = resting state fMRI; SC = structural connectivity; VOA = volume of activation; WMV = white matter volume.

**Fig. 4:**
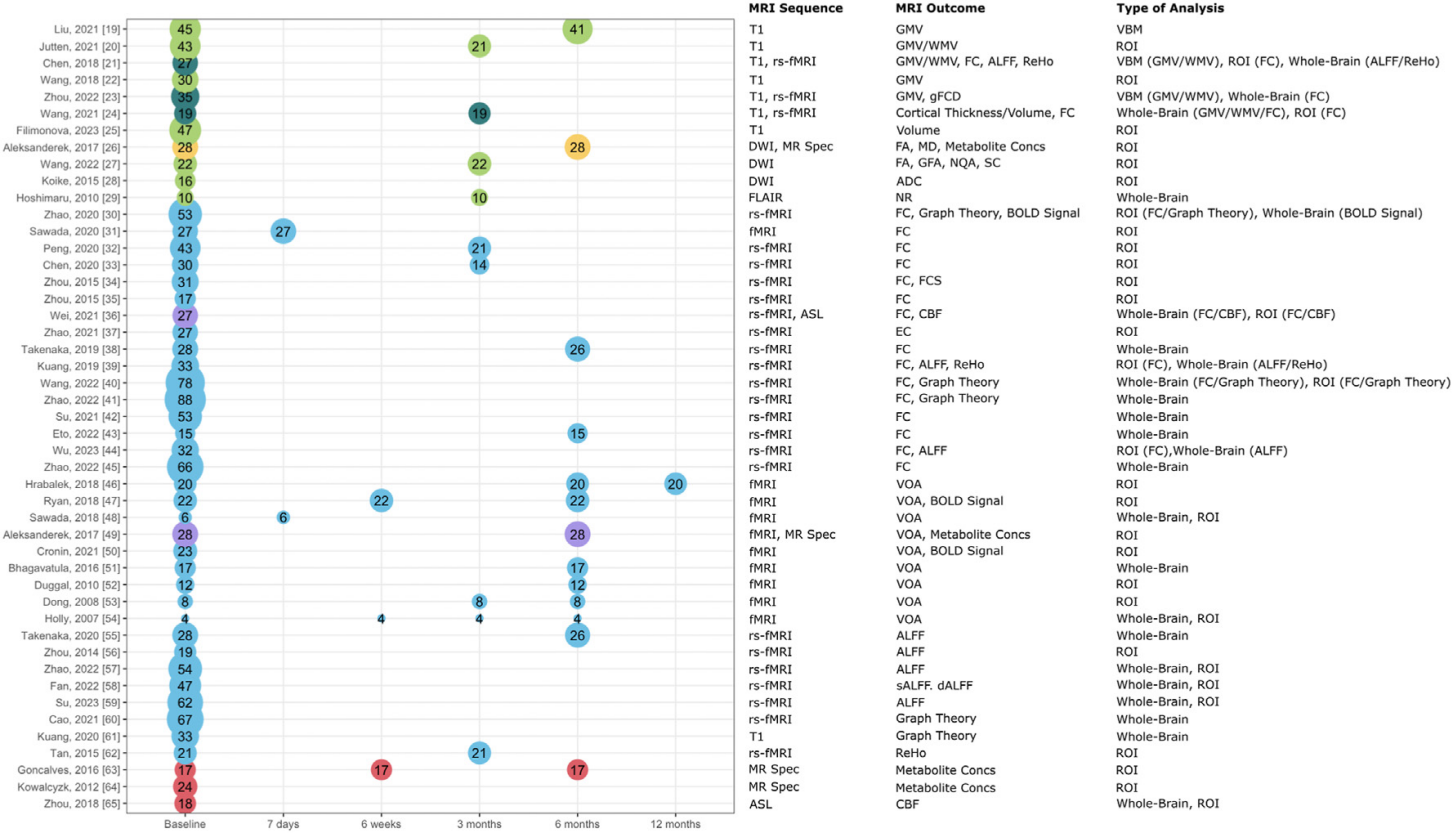
**Timeline of MRI scans of the 47 included studies.** Green circles represent studies that used structural MRI techniques, blue circles represent studies that used functional MRI techniques, red circles represent studies that used ‘other' MRI techniques, teal circles represent studies that used structural and functional MRI techniques, yellow circles represent studies that used structural and ‘other' MRI techniques; purple circles represent studies that used functional and ‘other' MRI techniques. Circle size is proportional to the number of patients with DCM in the analysis (equal to the number within the circle). ADC = apparent diffusion coefficient; ALFF = amplitude of low-frequency fluctuations; ASL = arterial spin labelling; BOLD Signal = blood oxygen level dependent signal; CBF = cerebral blood flow; dALFF = dynamic ALFF; DWI = diffusion-weighted imaging; EC = effective connectivity; FA = fractional anisotropy; FC = functional connectivity; FCS = FC strength; FLAIR = fluid attenuated inversion recovery; GFA = global FA; gFCD = global FC density; GMV = grey matter volume; MD = mean diffusivity; Metabolite Concs = metabolite concentrations; MR Spec = MR spectroscopy; NQA = normalised quantitative anisotropy; NR = not reported; ReHo = regional homogeneity; ROI = region of interest; rs-fMRI = resting state fMRI; sALFF = static ALFF; SC = structural connectivity; VBM = voxel-based morphometry; VOA = volume of activation; WMV = white matter volume.

The study characteristics of included papers are summarised in Fig. 2, Fig. 3, Fig. 4 and Supplementary Data 3. The number of patients with DCM included at baseline varied between four and 88 with a median study size of 28 (IQR 19–43). Mean age of patients with DCM included at baseline ranged from 47.5 to 67 years, with a median of 54.3 (IQR 51.3–58.1). Healthy controls (HCs) were used as a comparator in 79% (37/47) of studies. Of the remaining 21% (10/47) of studies, three studies compared post-surgical against pre-surgical patients with DCM,^27^, ^43^, ^63^ one compared patients with DCM to those with asymptomatic spinal cord compression,^40^ one compared patients with DCM to patients with cervical radiculopathy,^25^ three studies compared patients with DCM by either visual analogue scale (VAS) recovery,^31^ presence of depression,^45^ or motor evoked potentials (MEPs),^46^ and two studies used no comparators.^29^^,^^50^ In addition to comparing patients with DCM to HCs, Wu et al. (2023) also compared patients with DCM with an abnormal gait pattern to those with a normal gait.^44^ At least one quantitative measure of symptom severity (e.g., VAS) was reported in 96% (45/47) of included studies. Of the remaining two studies, Chen et al. (2020) analysed associations of Japanese orthopaedic association (JOA) and best corrected visual acuity (BCVA) but did not report on exact values,^33^ whilst Koike et al. (2015) did not report any quantitative measure of symptom severity.^28^ Over half of the included studies (25/47) investigated brain MRIs at baseline only (i.e., prior to any surgical intervention), whilst the remaining studies (22/47) investigated brain MRIs at baseline and at least one point after surgery, with five studies following up more than once. Most (38/47) of the included studies utilised the same type of MRI scanner for all of their scans, except six studies,^24^^,^^30^^,^^42^, ^46^, ^57^, ^63^ which used more than one type of MRI scanner across their protocol, two studies, which did not specify the type of MRI scanner beyond the brand,^32^^,^^51^ and one study,^29^ which did not report on the type of MRI scanner(s) used. The most utilised types of MRI scanner were: 3.0T Trio, Siemens (n = 14), 3.0T Discovery MR750, General Electric (n = 13), 3.0T Prisma, Siemens (n = 12), and 3.0T Ingenia, Philips (n = 4). There were no multi-centre studies amongst the included studies. Time points after surgery at which brain MRIs were taken include: 7 days (n = 2), 6 weeks (n = 3), 3 months (n = 9), 6 months (n = 13) and 12 months (n = 1) (Fig. 4).

**Fig. 5:**
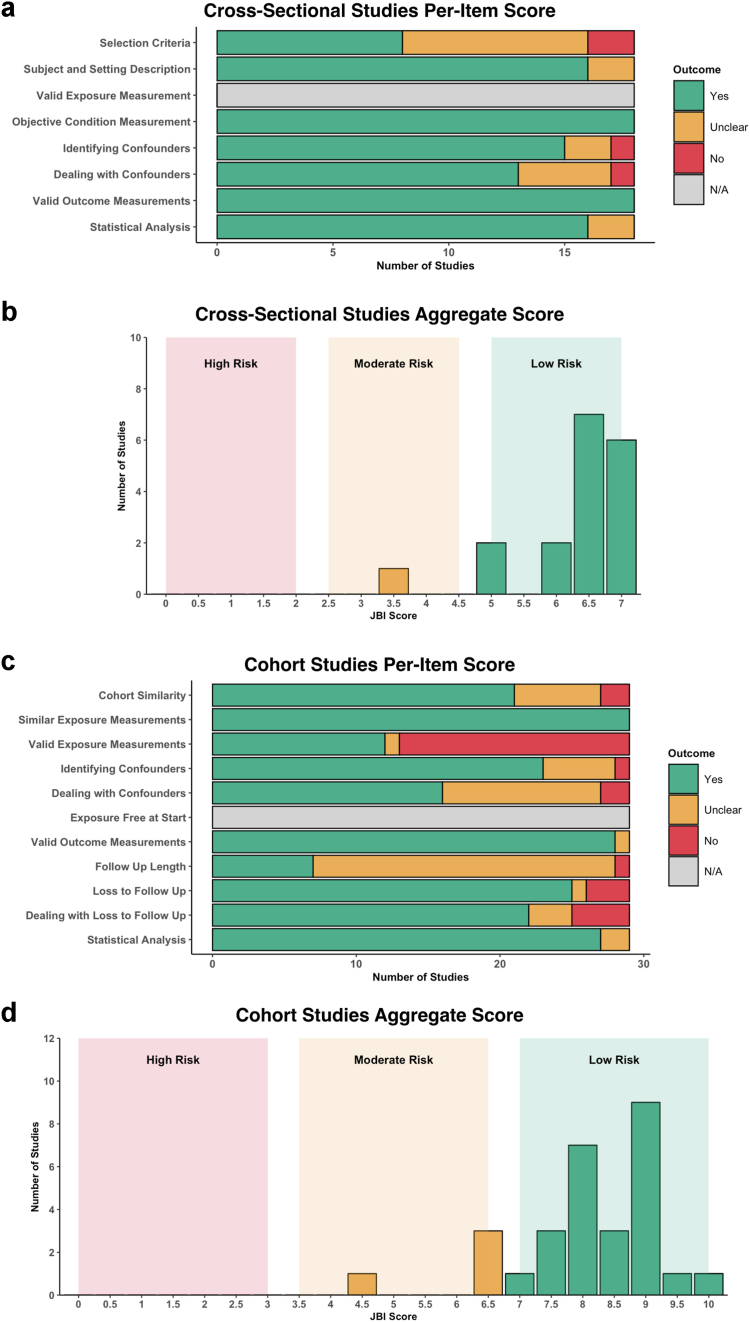
**Risk of bias assessment.** a. Histogram of distribution of per-item scores for cross-sectional studies, indicating for each item, the number of articles scoring ‘Yes’, ‘Unclear’, ‘No’ or ‘N/A’. b. Histogram of distribution of aggregate JBI scores for cross-sectional studies. ‘Yes’ scored 1 point per item, ‘Unclear’ scored 0.5 points per item and ‘No’ scored 0 points per item. c. Histogram of distribution of per-item scores for cohort studies, indicating for each item, the number of articles scoring ‘Yes’, ‘Unclear’, ‘No’ or ‘N/A’. d. Histogram of distribution of aggregate JBI scores for cohort studies. ‘Yes’ scored 1 point per item, ‘Unclear’ scored 0.5 points per item and ‘No’ scored 0 points per item.

Overall, 42 studies were deemed low risk, whilst five studies were deemed to have a moderate risk of bias (Supplementary Data 4). The distribution of per-item scores and aggregate scores from the JBI critical appraisal tool for cross-sectional and cohort studies is depicted in Fig. 5. Selection criteria, identification of confounders and subsequent strategies to deal with confounders were major sources of bias in both cross-sectional and cohort studies. Additionally, description of exposure (i.e., type of decompression surgery) and insufficient length of follow-up were further sources of bias in cohort studies.

In total, 1500 patients with DCM were analysed at baseline across the 47 included studies.

**Fig. 6:**
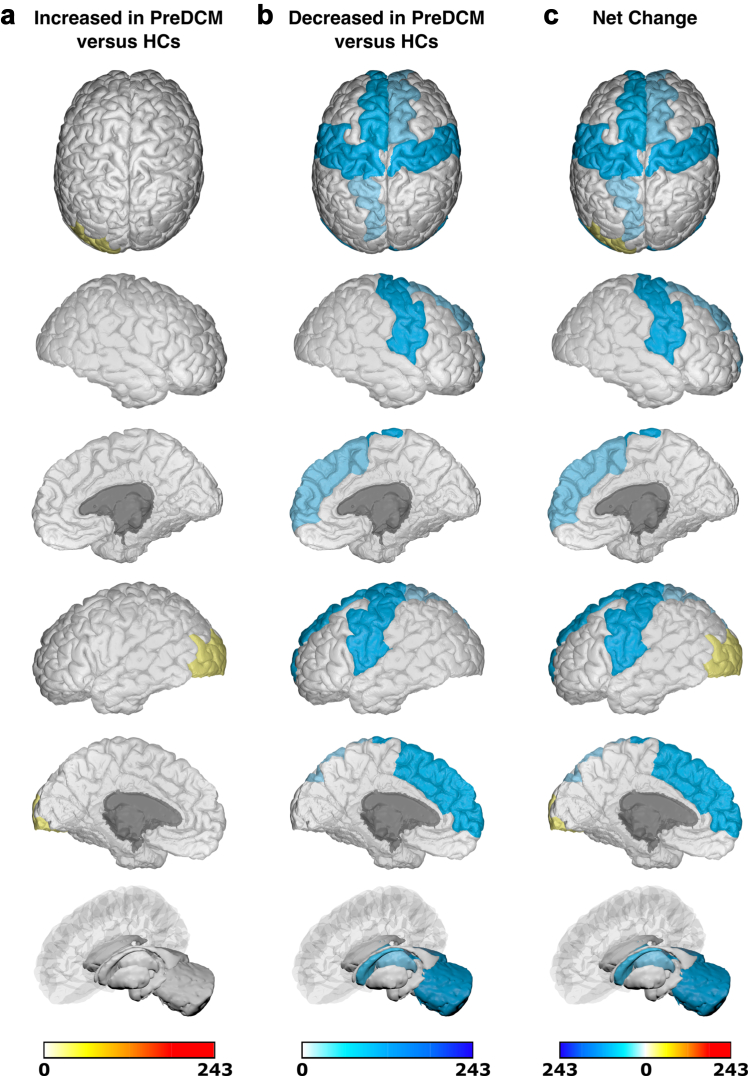
**Anatomical location of foci comparing changes in structural MRI measures in pre-surgical patients with DCM (PreDCM) against healthy controls (HCs).** a. Intensity map of foci showing increased structural MRI measures in PreDCM compared to HCs with min (no colour) value of 0 PreDCM and max (red colour) value of 243 PreDCM. b. Intensity map of foci showing decreased structural MRI measures in PreDCM compared to HCs with min (no colour) value of 0 PreDCM and max (blue colour) value of 243 PreDCM. c. Intensity map of foci showing net change in structural MRI measures in PreDCM compared to HCs with min (blue colour) value of net 243 PreDCM, mid-point (no colour) value of net 0 PreDCM and max (red colour) value of net 243 PreDCM.

11 studies examined structural changes in 322 patients with DCM before surgical intervention (Supplementary Data 5). Seven assessed GMV/WMV changes,^19^, ^20^, ^21^, ^22^, ^23^, ^24^, ^25^ two assessed FA,^26^^,^^27^ one assessed ADC,^28^ and one assessed qualitative changes on MRI.^29^ Structural changes in anatomical brain regions of 75% (243/322) of patients with DCM were compared against HCs and mapped onto the Desikan-Killiany atlas (Fig. 6). No regions were consistently reported as increased. Key regions found to have decreased changes in structural MRI measures include: superior frontal (L), precentral (L/R), postcentral (L/R), and cerebellum (L/R). These same regions are highlighted for net decrease in structural MRI measures.

**Fig. 7:**
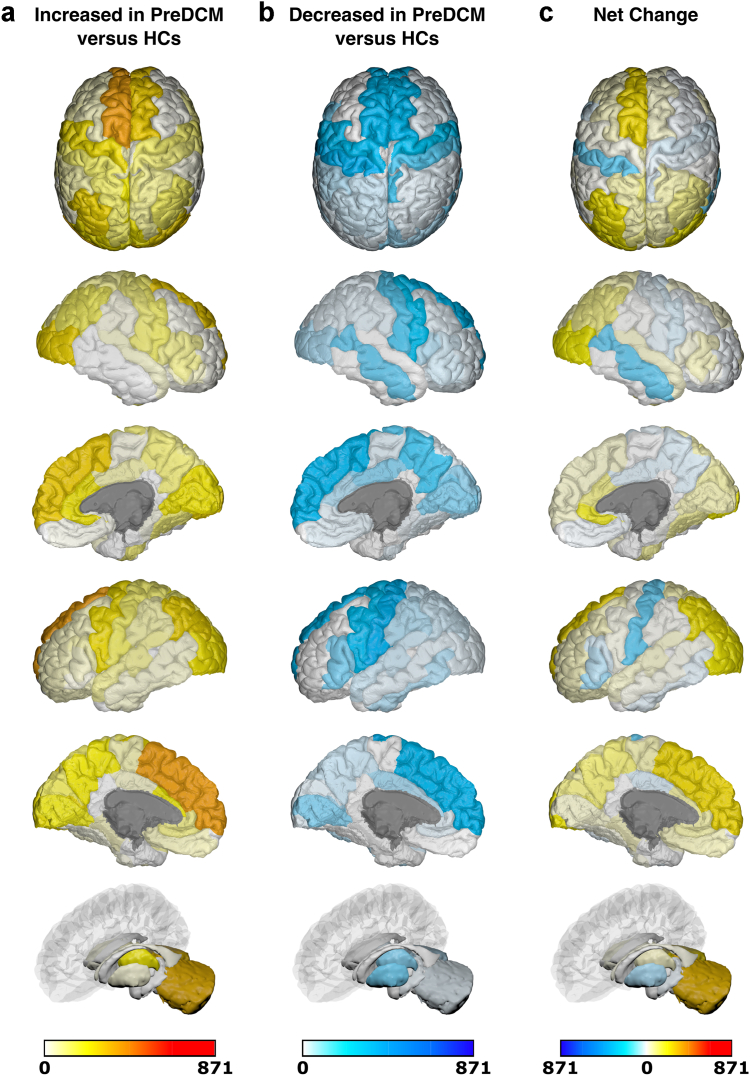
**Anatomical location of foci comparing changes in functional MRI measures in pre-surgical patients with DCM (PreDCM) against healthy controls (HCs).** a. Intensity map of foci showing increased functional MRI measures in PreDCM compared to HCs with min (no colour) value of 0 PreDCM and max (red colour) value of 871 PreDCM. b. Intensity map of foci showing decreased functional MRI measures in PreDCM compared to HCs with min (no colour) value of 0 PreDCM and max (blue colour) value of 871 PreDCM. c. Intensity map of foci showing net change in functional MRI measures in PreDCM compared to HCs with min (blue colour) value of net 871 PreDCM, mid-point (no colour) value of net 0 PreDCM and max (red colour) value of net 871 PreDCM.

A total of 36 studies explored functional changes in 1200 patients with DCM before surgical intervention (Supplementary Data 6). Of these, 19 assessed FC,^21^^,^^23^^,^^24^^,^^30^, ^31^, ^32^, ^33^, ^34^, ^35^, ^36^, ^37^, ^38^, ^39^, ^40^, ^41^, ^42^, ^43^, ^44^, ^45^ nine assessed activation/VOA,^46^, ^47^, ^48^, ^49^, ^50^, ^51^, ^52^, ^53^, ^54^ eight assessed ALFF,^21^, ^39^, ^44^, ^55^, ^56^, ^57^, ^58^, ^59^ five utilised graph theory analysis,^30^, ^40^, ^41^, ^60^, ^61^ three assessed BOLD signal changes,^30^, ^47^, ^50^ and three assessed ReHo.^21^, ^39^, ^62^ Functional changes in anatomical brain regions of 73% (871/1200) of patients with DCM were compared against HCs and mapped onto the Desikan-Killiany atlas (Fig. 7). Key regions found to have increased changes in functional MRI measures include: superior frontal (L/R), inferior parietal (L), lateral occipital (R), and cerebellum (L/R). Key regions found to have decreased changes in functional MRI measures include: postcentral (L). Key regions found to have net increased changes in functional MRI measures include: superior frontal (L), anterior cingulate (R), inferior parietal (L), lateral occipital (L/R), and cerebellum (L/R). Key regions found to have net decreased changes in functional MRI measures include: postcentral (L), and middle temporal (R).

Six studies utilised ‘other’ MRI techniques in 142 patients with DCM before surgical intervention (Supplementary Data 7), of which four analysed metabolite concentrations,^26^, ^49^, ^63^, ^64^ and two analysed cerebral blood flow.^36^^,^^65^

In total, 425 patients with DCM were analysed post-surgery across 22 of the included studies.

**Fig. 8:**
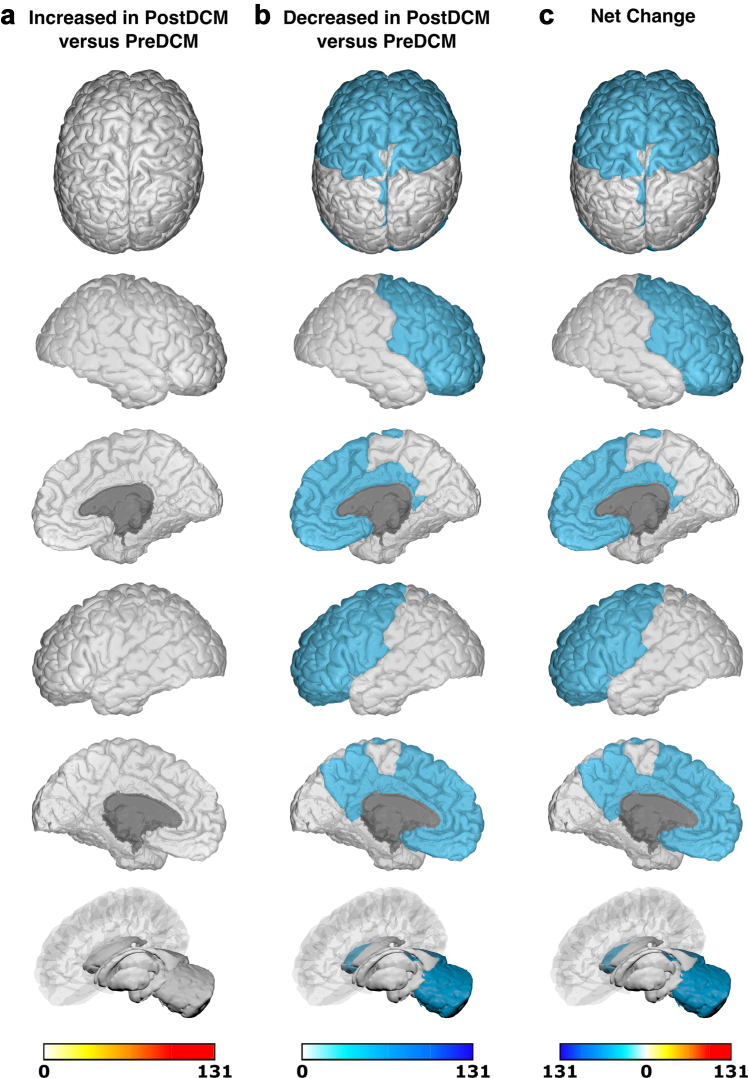
**Anatomical location of foci comparing changes in structural MRI measures in post-surgical patients with DCM (PostDCM) against pre-surgical patients with DCM (PreDCM).** a. Intensity map of foci showing increased structural MRI measures in PostDCM compared to PreDCM with min (no colour) value of 0 PostDCM and max (red colour) value of 131 PostDCM. b. Intensity map of foci showing decreased structural MRI measures in PostDCM compared to PreDCM with min (no colour) value of 0 PostDCM and max (blue colour) value of 131 PostDCM. c. Intensity map of foci showing net change in structural MRI measures in PostDCM compared to PreDCM with min (blue colour) value of net 131 PostDCM, mid-point (no colour) value of net 0 PostDCM and max (red colour) value of net 131 PostDCM.

Six studies examined structural changes in 141 patients with DCM after surgical intervention (Supplementary Data 8). Three assessed GMV/WMV,^19^, ^20^, ^24^ two assessed FA,^26^, ^27^ one assessed SC,^27^ and one assessed qualitative changes on MRI.^29^ Structural changes in anatomical brain regions of 93% (131/141) of post-surgical patients with DCM were compared against pre-surgical patients with DCM and mapped onto the Desikan-Killiany atlas (Fig. 8). No regions were found to have increased changes in structural MRI measures post-surgery. Key regions found to have decreased changes in structural MRI measures post-surgery include: cerebellum (L/R). These same regions are highlighted for net decrease in structural MRI measures post-surgery. Only two studies compared structural changes across anatomical brain regions of post-surgical patients with DCM against HCs,^19^, ^26^ so no brain maps were created here.

**Fig. 9:**
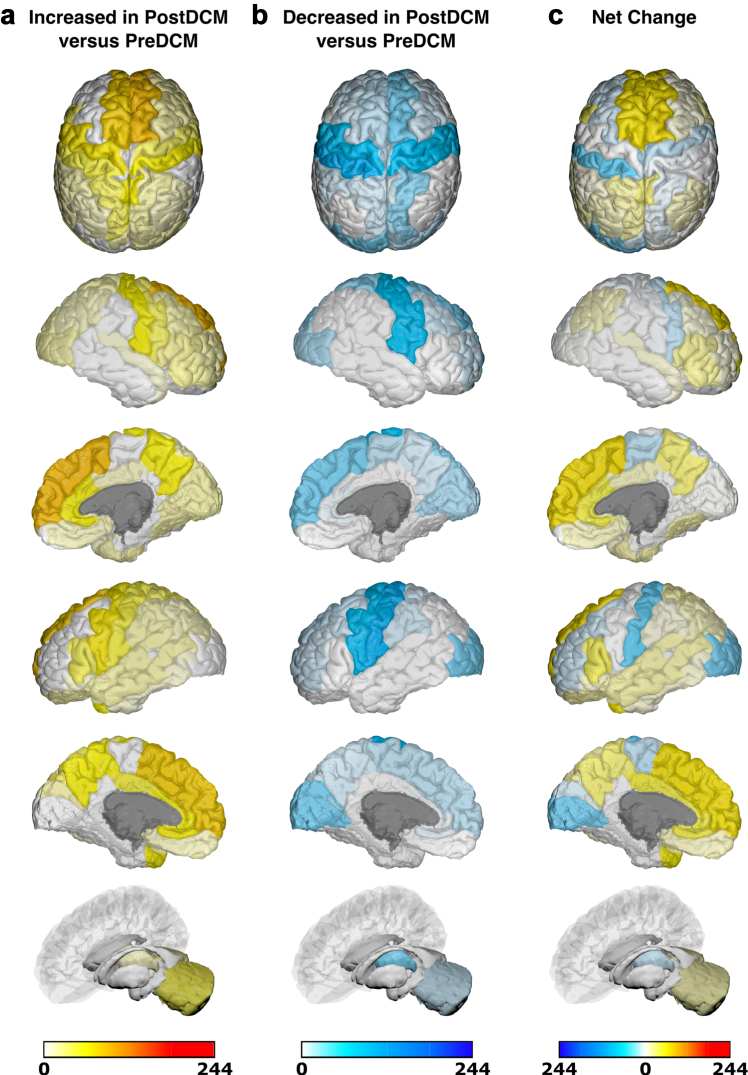
**Anatomical location of foci comparing changes in functional MRI measures in post-surgical patients with DCM (PostDCM) against pre-surgical patients with DCM (PreDCM).** a. Intensity map of foci showing increased functional MRI measures in PostDCM compared to PreDCM with min (no colour) value of 0 PostDCM and max (red colour) value of 244 PostDCM. b. Intensity map of foci showing decreased functional MRI measures in PostDCM compared to PreDCM with min (no colour) value of 0 PostDCM and max (blue colour) value of 244 PostDCM. c. Intensity map of foci showing net change in functional MRI measures in PostDCM compared to PreDCM with min (blue colour) value of net 244 PostDCM, mid-point (no colour) value of net 0 PostDCM and max (red colour) value of net 244 PostDCM.

A total of 16 studies explored functional changes in 286 patients with DCM after surgical intervention (Supplementary Data 9). Six assessed FC,^24^, ^31^, ^32^, ^33^, ^38^, ^43^ eight assessed activation/VOA,^46^, ^47^, ^48^, ^49^, ^51^, ^52^, ^53^, ^54^ one assessed ALFF,^55^ one assessed BOLD signal changes,^47^ and one assessed ReHo.^62^ Functional changes in anatomical brain regions of 85% (244/286) of post-surgical patients with DCM were compared against pre-surgical patients with DCM and mapped onto the Desikan-Killiany atlas (Fig. 9). Key regions found to have increased changes in functional MRI measures post-surgery include: superior frontal (L/R). Key regions found to have decreased changes in functional MRI measures post-surgery include: postcentral (L). Key regions found to have net increased changes in functional MRI measures post-surgery include: superior frontal (L/R), and temporal pole (L). Key regions found to have net decreased changes in functional MRI measures post-surgery include: postcentral (L).

**Fig. 10:**
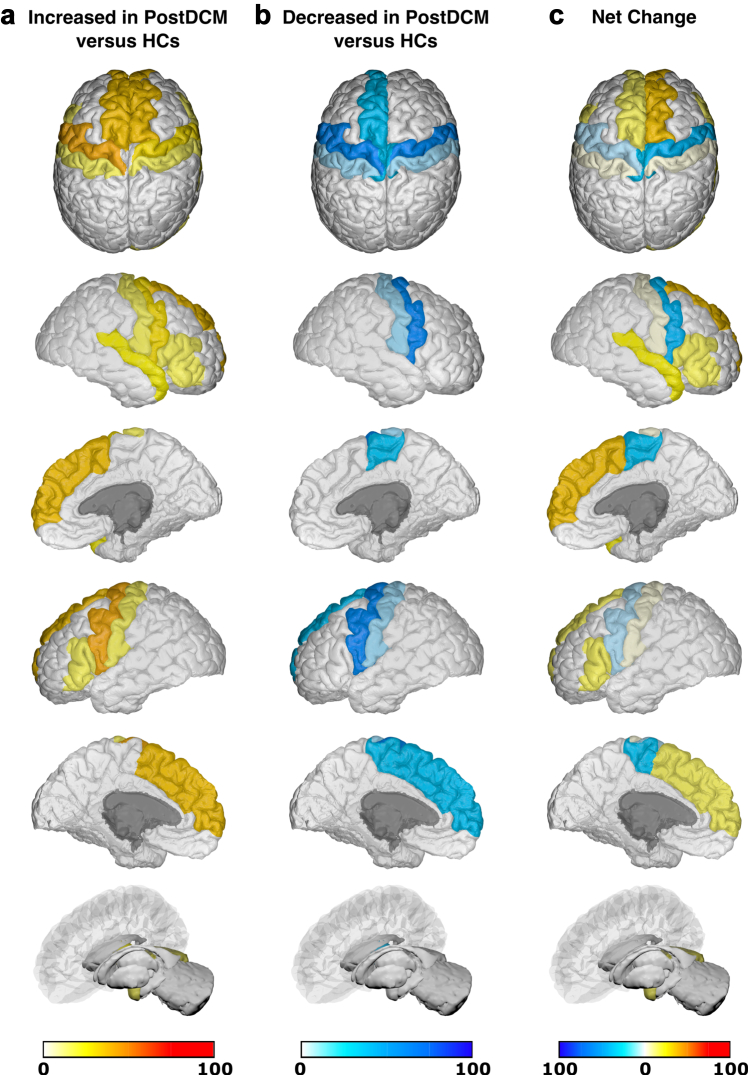
**Anatomical location of foci comparing changes in functional MRI measures in post-surgical patients with DCM (PostDCM) against healthy controls (HCs).** a. Intensity map of foci showing increased functional MRI measures in PostDCM compared to HCs with min (no colour) value of 0 PostDCM and max (red colour) value of 100 PostDCM. b. Intensity map of foci showing decreased functional MRI measures in PostDCM compared to HCs with min (no colour) value of 0 PostDCM and max (blue colour) value of 100 PostDCM. c. Intensity map of foci showing net change in functional MRI measures in PostDCM compared to HCs with min (blue colour) value of net 100 PostDCM, mid-point (no colour) value of net 0 PostDCM and max (red colour) value of net 100 PostDCM.

Functional changes in anatomical brain regions of 33% (100/306) of post-surgical patients with DCM were compared against HCs and mapped onto the Desikan-Killiany atlas (Fig. 10). Key regions found to have increased changes in functional MRI measures post-surgery include: superior frontal (L/R), and precentral (L). Key regions found to have decreased changes in functional MRI measures post-surgery include: precentral (L/R). Key regions found to have net increased changes in functional MRI measures post-surgery include: superior frontal (L/R), superior temporal (R), amygdala (L), and cerebellum (R). Key regions found to have net decreased changes in functional MRI measures post-surgery include: paracentral (L/R), and precentral (R).

Three studies utilised ‘other’ MRI techniques in 73 patients with DCM after surgical intervention (Supplementary Data 10), with all examining metabolite concentrations.^26^, ^49^, ^63^

A total of 43 papers explored the correlational relationships between brain changes before surgery and clinical measures (Table 3). Studies analysed associations with: GMV/WMV (n = 6),^19^, ^20^, ^22^, ^23^, ^24^, ^25^ FC (n = 15),^23^, ^24^, ^30^, ^31^, ^32^, ^33^, ^34^, ^35^, ^36^, ^37^, ^38^, ^39^, ^41^, ^44^, ^45^ activation/VOA (n = 4),^46^, ^47^, ^48^, ^50^ ALFF (n = 8),^21^, ^39^, ^44^, ^55^, ^56^, ^57^, ^58^, ^59^ graph theory (n = 2),^30^^,^^60^ BOLD signal (n = 2),^30^^,^^50^ ReHo (n = 3),^21^^,^^39^^,^^62^ metabolites (n = 2),^49^^,^^64^ and CBF (n = 2).^36^^,^^65^ Correlations were most commonly reported with the JOA, although several studies reported no associations.

**Table 3:** Summary of reported associations between MRI brain changes and clinical characteristics in patients with DCM before surgical intervention.

MRI outcome	Reference	Associations with baseline MRI measurements
GMV/WMV	^19^	No association between GMV and JOA, NDI, disease duration
	^20^	MUCCA volume ↗ WMV overall, primary motor cortex, cerebellum Symptom severity subgroup analysis showed higher GMV in primary motor cortex in none-mild compared to moderate and severe in BA4a and none-mild compared to severe in BA4p, in supplementary motor area in none-mild compared to moderate and severe, in cerebellum areas IV, V, VIIIa and VIIIb in none-mild compared to severe. No WMV differences in symptom severity subgroup analysis.
	^22^	Upper motor (JOA) ↗ GMV in L BA1, L and R BA3a, R BA4p Lower motor (JOA)↗ GMV in L BA3b Sphincter dysfunction (JOA) ↗ GMV in L BA6 No association between GMV and JOA or sensory (JOA)
	^23^	No association between GMV and JOA or disease duration
	^24^	mJOA ↗ cortical thickness of L pars triangularis, L precentral gyrus, L superior temporal gyrus, L lateral occipital gyrus, R caudal anterior cingulate, R superior parietal lobule, R superior frontal gyrus, R postcentral gyrus, R paracentral gyrus
	^25^	CSA at level of maximum compression ↗ medulla volume FA at C2 ↗ medulla volume No association between volumes of pons, midbrain or total brainstem and CSA at level of maximum compression, structural changes of spinal cord or disease duration, although disease duration negatively trended with medulla volume Patients with DCM for >2 years had a smaller medulla volume (compared to those with DCM <2 years)
FC	^23^	No association between gFCD and pre-JOA or disease duration
	^24^	No association between FC and symptom duration
	^30^	MoCA ↗ FC between cerebellum and L superior frontal gyrus, L posterior cerebellum lobe and L mid-posterior cerebellum, between L inferior temporal gyrus and L middle frontal gyrus JOA ↗ FC between L inferior temporal gyrus and B calcarine gyrus, between L precentral gyrus and R calcarine gyrus
	^31^	VAS improvement ratio ↘ FC between middle frontal gyrus and L postcentral gyrus
	^32^	Sensory (JOA) ↗ FC between L thalamus and B lingual gyrus/cuneus/R cerebellar posterior lobe
	^33^	JOA ↘ FC of L BA19, R BA19 Pre-BCVA of OD ↗ FC of L BA7, R BA7 Pre-BCVA of OS ↗ FC of L BA7
	^34^	JOA ↗ mean normalized-FCS in L premotor ventral/precentral operculum, R operculum parietale, R primary sensory cortex FA at C2 trended with mean normalized-FCS in L inferior parietal lobule, R primary sensory cortex No association between mean normalized-FCS and NDI or FA at most severe level JOA ↘ correlation coefficient between R primary sensory cortex BA3b and R insula FA at C2 ↘ correlation coefficient between L premotor ventral/precentral operculum and L premotor dorsal, between R inferior parietal lobule and R premotor dorsal, between R superior parietal lobule and R premotor ventral/insula FA at C2 ↘ correlation coefficient between R primary sensory cortex BA3b and BA2/1
	^35^	*Slow-5 band:* No association between rsFC and disease duration, JOA, NDI, FA at C2 or most severe level *Slow-4 band:* FA at C2 ↗ rsFC coefficients of R middle frontal gyrus, R postcentral gyrus-1, R postcentral gyrus-3 FA at most severe level ↗ rsFC coefficients of R middle frontal gyrus No association between rsFC and JOA or NDI
	^36^	*Region wise:* Pre-JOA ↗ FCS in L precentral gyrus Post-JOA ↗ FCS in L middle frontal gyrus *Voxel wise:* Pre-JOA ↗ FCS in L precentral gyrus MoCA ↗ FCS in R hippocampus *ROI wise:* Pre-JOA ↘ FCS in R calcarine gyrus, L thalamus, L calcarine, L and R precuneus Pre-JOA ↗ FCS in L precentral gyrus JOA recovery ↗ FCS in L and R precuneus, L calcarine Post-JOA ↘ FCS in L calcarine gyrus, R thalamus
	^37^	Pre-BCVA of OD and OS ↗ EC of cerebellum self-connection Improvement in BCVA of OS ↗ EC from cerebellum to L secondary visual cortex (unidirectional) Improvement in BCVA of OD ↗ EC from cerebellum to R secondary visual cortex (unidirectional)
	^38^	Post-gain in 10-s test ↗ FC in 15 clusters (including between R superior frontal gyrus and primary visual cortex, L intracalcarine cortex, L lingual gyrus) Post-gain in 10-s test ↘ FC in 1 cluster JOA-UEM ↘ FC in 3 clusters No association between FC and JOA-UES, JOACMEQ-UEF Formulae developed to predict post-gain in 10-s test using FC between L lingual gyrus and R superior frontal gyrus
	^39^	No association between FC and disease duration, JOA or NDI
	^41^	No association between dFC and JOA or NDI
	^44^	mJOA ↘ FC between caudate and L angular gyrus, L and R precuneus, L and R posterior cingulate cortex mJOACMEQ-LEF ↘ FC between caudate and L angular gyrus, L and R precuneus, L and R posterior cingulate cortex CSA of CST at C2 ↘ FC between caudate and R precuneus, L posterior cingulate cortex, R posterior cingulate cortex
	^45^	JOA recovery rate ↘ FC between L dorsal caudate and L inferior frontal operculum
Activation/VOA	^46^	No association between motor activation and pre-CMCT
	^47^	mJOA ↗ VOA of contralateral M1
	^48^	SDS ↗ activation of anterior cingulate cortex, thalamus
	^50^	*Left hand tapping:* Total SCC volume ↗ VOA in contralateral primary motor cortex, contralateral primary sensory cortex, contralateral premotor cortex, cerebellum, putamen, caudate, thalamus *Right hand tapping:* Total SCC volume ↗ VOA in contralateral primary motor cortex, contralateral premotor cortex, contralateral supplementary motor area, cerebellum, putamen, caudate, thalamus Total SCC volume trended with VOA in primary sensory cortex No association between subcortical activation and mJOA, between motor activation and symptom duration
ALFF	^21^	Pre-BCVA of OD ↗ ALFF of BA18/17, L and R calcarine, L and R precuneus Pre-BCVA of OS ↗ ALFF of L and R calcarine
	^39^	No association between zALFF and disease duration, JOA or NDI
	^44^	mJOA ↗ zALFF of caudate mJOACMEQ-LES ↗ zALFF of caudate FA of spinocerebellar tract at C2 ↗ zALFF of caudate FA of fasciculus cuneatus at C2 ↗ zALFF of caudate FA of fasciculus gracilis at C2 ↘ zALFF of R paracentral lobule
	^55^	Improvement in JOACMEQ-UEF ↗ ALFF in L and R frontal pole, L parsopercularis of inferior frontal gyrus No association between ALFF and improvements in JOA-UEM, JOA-UES or 10-s test
	^56^	FA at C2 ↘ zALFF of R precentral gyrus, R post central gyrus No association between zALFF and disease duration, JOA or FA at most severe level
	^57^	Pre-JOA ↘ zALFF of L precentral gyrus Pre-JOA ↗ zALFF of L and R superior frontal gyrus M1 ALFF can increase prediction of JOA recovery using SVR with feature set including age, gender, duration of myelopathy, pre-JOA and presence of T2 high signal in cervical MR
	^58^	No association between ALFF and pre-JOA or JOA recovery sALFF using voxel in frontal cortices can successfully predict JOA recovery
	^59^	Postoperative pain intensity ↗ ALFF of middle cingulate cortex Pain threshold ↘ ALFF of middle cingulate cortex ALFF of middle cingulate cortex can increase prediction of postoperative axial pain with feature set including JOA, preoperative pain, PVAQ, pain threshold, age, gender and education years
Graph theory	^30^	MoCA ↗ altered shortest path, global efficiency No association between assortativity or hierarchy and MoCA
	^60^	JOA ↗ Eglob, nodal degree in L postcentral gyrus, nodal efficiency in L putamen JOA ↘ Lp No association between global or network parameters and disease duration or NDI score
BOLD signal	^30^	MMSE ↘ BOLD signal variability in R putamen MoCA ↗ BOLD signal variability in L inferior parietal lobule JOA ↗ BOLD signal variability R mid-cingulate gyrus, L precentral gyrus
	^50^	*Left hand tapping:* Total SCC volume ↗ % BOLD signal in contralateral primary motor cortex, contralateral primary sensory cortex, cerebellum, putamen, caudate, thalamus mJOA ↗ % BOLD signal in contralateral primary cortex, contralateral primary sensory cortex *Right hand tapping:* Total SCC volume ↗ % BOLD signal in contralateral primary motor cortex, contralateral primary sensory cortex, contralateral supplementary motor area, cerebellum, putamen, caudate, thalamus mJOA ↗ % BOLD signal in contralateral primary cortex, contralateral primary sensory cortex
ReHo	^21^	Pre-BCVA of OD ↗ ReHo of BA19/18/17, L and R calcarine, L and R precuneus Pre-BCVA of OS ↗ ReHo of BA19/18/17, L and R calcarine, R precuneus Post-BCVA of OD ↗ ReHo of BA19/18/17 Post-BCVA of OS ↗ ReHo of BA19/18/17
	^39^	No association between zReHo and JOA, NDI or disease duration
	^62^	No association between ReHo and JOA, NDI or disease duration
Metabolites	^49^	mJOA ↘ NAA/Cr Change in mJOA ↘ NAA/Cr No association between NAA/Cr and disease duration
	^64^	No association between metabolite concentrations and disease duration, mJOA, NDI or ASIA
CBF	^36^	*Region wise:* Post-JOA ↗ CBF in L middle frontal gyrus *ROI wise:* Pre-JOA ↘ CBF in R calcarine gyrus, L thalamus Post-JOA ↘ CBF in L calcarine gyrus, R thalamus
	^65^	JOA ↘ CBF of R postcentral gyrus NDI ↘ L and R premotor ventral/precentral operculum, L supramarginal gyrus, B supplementary motor area, R postcentral gyrus, L dorsal anterior cingulate cortex, R dorsolateral premotor cortex No association between CBF and disease duration, FA at C2 or most severe level

Arrows indicate significant positive (↗) and negative (↘) correlation.

ALFF = amplitude of low-frequency fluctuation; ASIA = American spinal injury association impairment scale; BA = Brodmann area; BCVA = best corrected visual acuity; BOLD signal = blood oxygen level dependent signal; CBF = cerebral blood flow; CMCT = central motor conduction time; CSA = cross-sectional area; CST = corticospinal tract; DCM = degenerative cervical myelopathy; dFC = dynamic FC; EC = effective connectivity; Eglob = global efficiency; FA = fractional anisotropy; FC = functional connectivity; FCS = FC strength; gFCD = global FC density; GMV = grey matter volume; JOA = Japanese orthopaedic associate score; JOA-UEM = JOA-upper extremity motor score; JOA-UES = JOA-upper extremity sensory score; JOACMEQ-LEF = JOA cervical myelopathy evaluation questionnaire for lower extremity function; JOACMEQ-UEF = JOA cervical myelopathy evaluation questionnaire for upper extremity function; Lp = characteristic path length; mJOA = modified JOA; MMSE = mini-mental state examination; MoCA = Montreal cognitive assessment; MUCCA = mean upper cervical cord area; NAA/Cr = N-acetylaspartate/creatine ratio; NDI = neck disability index; OD = oculus dexter; OS = oculus sinister; Post = after surgical intervention; Pre = before surgical intervention; PVAQ = pain vigilance and awareness questionnaire; ReHo = regional homogeneity; rsFC = resting state FC; sALFF = static ALFF; SCC = spinal cord compression; SDS = Zung self-rating depression scale; SVR = support vector regression; VAS = visual analogue scale; VOA = volume of activation; WMV = white matter volume.

A total of 12 papers studied the relationships between brain changes after surgery and clinical measures (Table 4). Studies analysed associations with: GMV/WMV (n = 3),^19^, ^20^, ^24^ FA (n = 1),^27^ SC (n = 1),^27^ FC (n = 3),^24^, ^32^, ^43^ activation/VOA (n = 3),^46^, ^47^, ^53^ BOLD signal (n = 1),^47^ ReHo (n = 1),^62^ and metabolites (n = 2).^49^^,^^63^ Similar to pre-surgery, post-surgical correlations were most commonly reported with the JOA, although several studies reported no associations.

**Table 4:** Summary of reported associations between MRI brain changes and clinical characteristics in patients with DCM after surgical intervention.

MRI outcome	Reference	Associations with after surgery MRI measurements
GMV/WMV	^19^	No association between GMV and JOA, NDI, disease duration
	^20^	mJOA trended with GMV in primary motor cortex areas BA4a and BA4p
	^24^	No association between cortical thickness and neurological improvement
FA	^27^	mJOA ↗ FA in R internal capsule (anterior limb, posterior limb, retrolenticular part), corpus collosum (body, genu), corona radiata (L anterior, B posterior, B superior), B corticospinal tract, R cerebral peduncle, R inferior cerebellar peduncle, middle cerebellar peduncle, R superior cerebellar peduncle, R medial lemniscus, R fornix stria terminalis, B superior longitudinal fasciculus, thalamus mJOA ↘ FA in L internal capsule (anterior limb), B external capsule mJOA ↗ NQA in B internal capsules, R external capsule, B anterior and superior corona radiata, corpus collosum (body, genu), B corticospinal tract, B cingulum, B superior fronto-occipital fasciculus, B superior longitudinal fasciculus
SC	^27^	mJOA ↗ SC between cerebellum and B lingual gyri, between primary sensorimotor regions and cortical regions (including L precuneus, R caudal middle frontal gyrus, limbic system) mJOA ↘ SC between insular cortex with temporal and parietal lobes, between limbic system with frontal lobes and basal ganglia
FC	^24^	mJOA ↗ FC between L cerebellum and sensorimotor network (including B precentral gyri and R postcentral gyrus) Neurological improvement ↘ FC within cerebellum (especially posterior cerebellum) No association between FC and symptom duration
	^32^	Upper motor (JOA) ↗ FC between thalamus and paracentral lobe/precentral gyrus
	^43^	JOA ↗ FC between R lateral part of sensorimotor network and L precentral gyrus JOACMEQ-UEF ↗ FC between R and L lateral part of sensorimotor network NRS ↘ FC between R lateral part of sensorimotor network and L postcentral gyrus
Activation/VOA	^46^	Pre-CMCT ↗ motor activation in ipsilateral sensorimotor cortex at 12 months. No association between motor activation and pre-CMCT at 6 months.
	^47^	mJOA ↗ VOA of contralateral primary motor cortex at 6 months
	^53^	Change in mJOA-upper at 3 months from baseline ↗ change in activation magnitude during pinch in contralateral primary sensory cortex Increased cord diameter at 3 months from baseline ↗ change in activation magnitude during pinch in contralateral primary motor cortex Change in mJOA-upper at 3 months from baseline ↗ change in VOA during wrist extension in contralateral primary motor cortex, ipsilateral dorsal premotor area, ipsilateral supplementary motor area Change in mJOA-upper at 6 months from baseline ↗ change in VOA during wrist extension in contralateral supplementary motor area
BOLD signal	^47^	mJOA ↗ % BOLD signal of ipsilateral supplementary motor area at 6 months mJOA ↗ change in % BOLD signal of contralateral primary motor cortex at 6 months from baseline, ipsilateral supplementary motor area at 6 months from baseline
ReHo	^62^	No association between ReHo and JOA, NDI or disease duration
Metabolites	^49^	Change in mJOA ↘ change in NAA/Cr at 6 months from baseline No association between NAA/Cr and disease duration
	^63^	Change in motor (JOA) after 6 months ↗ change in NAA in motor cortex after 6 months Similar temporal pattern in changes between time points in motor and sensory portions of ASIA questionnaire and concentration of NAA in motor cortex

Arrows indicate significant positive (↗) and negative (↘) correlation.

ASIA = American spinal injury association impairment scale; BA = Brodmann area; BOLD signal = blood oxygen level dependent signal; CMCT = central motor conduction time; FA = fractional anisotropy; FC = functional connectivity; GMV = grey matter volume; JOA = Japanese orthopaedic associate score; JOACMEQ-UEF = JOA cervical myelopathy evaluation questionnaire for upper extremity function; mJOA = modified JOA; NAA/Cr = N-acetylaspartate/creatine ratio; NDI = neck disability index; NQA = normalized quantitative anisotropy; NRS = numerical rating scale; Post = after surgical intervention; Pre = before surgical intervention; ReHo = regional homogeneity; SC = structural connectivity; VOA = volume of activation; WMV = white matter volume.

## Discussion

The literature surrounding brain changes in DCM has been rapidly expanding in recent years, with over half (25/47) of included studies in this review being published in the last four years. This review aimed to take stock of the current literature to assess MRI evidence of brain changes in individuals with DCM at baseline and following surgery, and whether such changes are associated with clinical measures. We found that most studies, but not all, reported brain changes in patients with DCM both before and after surgery, suggesting pathological processes and plasticity occur beyond the level of the spinal cord. Most of these studies further went on to report associations between MRI outcomes and clinical measures.

Conceptually brain changes in DCM could be explained by: (1) decreased MRI measures representing ‘pathological changes’ driven by DCM that may contribute to symptoms, and (2) increased MRI measures representing ‘compensatory changes’ that might attempt to preserve function in patients with DCM. Whilst our explanation for the observed brain changes remains a hypothesis at this stage, it is supported by the broader literature, particularly the notion that spinal cord injury due to DCM can result in synaptic plasticity in an effort to preserve function.^66^ Our hypothesis is further supported by the evidence base in SCI, where a meta-analysis of MRI brain activation in traumatic SCI similarly concluded that brain changes were likely a function of direct loss of function (comparable to our ‘pathological changes’ hypothesis) and adaptive cortical reorganization (comparable to our ‘compensatory changes’ hypothesis).^67^ Moreover, a more recent voxel-based meta-analysis of structural MRI changes proposed that significant insular atrophy following SCI may in part contribute to the subsequent depression reported in patients,^68^ providing additional support for our hypothesis that ‘pathological changes’ beyond the spinal cord occur and may help to explain the occurrence of symptoms in DCM.

Keeping the short-term follow up of most longitudinal studies in consideration, post-surgical versus pre-surgical DCM changes were mostly confined to the SMN. By contrast, pre-surgical DCM versus HCs changes were more widespread, most commonly involving regions of the SMN, VN and DMN, as well as subcortical structures including the thalamus and cerebellum.

Historically, symptoms of DCM have been presumed to be secondary to neuronal damage at the level of the cervical spinal cord. The association of symptoms poorly explained by spinal cord dysfunction alone, however, leads to the hypothesis that brain changes could play a more active role than first suggested in symptom development.^69^ This notion is consistent with rat models of SCI, where significant apoptosis was reported in supraspinal structures, including the hippocampus.^70^ The exact underlying mechanisms behind this are not completely clear, but one possibility is that neuronal damage in the cervical spine results in retrograde neuronal degeneration, thereby affecting the brain.

The SMN is the key brain network involved in regulating sensory and motor function. Its dysfunction has been heavily implicated to be a driving cause of sensory and motor deficits in several neurological disorders, including Parkinson's disease.^71^ Moreover, disruption of connectivity in the SMN of sensory-deprived patients has been suggested to cause their impairments in sensory-guided movements.^72^ It is likely that damage to the cervical spinal cord in DCM results in similar ‘pathological changes’ in the SMN, due to its direct connection via the corticospinal tract, and these changes contribute to motor deficits observed in DCM.^73^

Symptoms such as blurred vision and cognitive impairment are unlikely integrated at the level of the spinal cord, so their occurrence in both DCM,^13^ and other spinal disorders (such as SCI),^74^ currently renders them ‘atypical’ in the textbooks. ‘Pathological changes’ in the VN provide a more intuitive explanation for reported cases of impaired vision in DCM. Similarly, the DMN and thalamus are important in cognitive processes, so ‘pathological changes’ in these regions likely contribute to the cognitive deficits reported in DCM.

Following ‘pathological change’ at both the level of the spinal cord and brain, considerable plasticity and remodelling occurs to reduce subsequent loss in function – so-called ‘compensatory changes’.^75^ Neuronal injury resulting in neuronal reorganisation has been described for SCI and multiple sclerosis.^76^^,^^77^ Reorganisation broadly utilises two key mechanisms: modification of pre-existing circuity and development of novel circuitry. Genetic and non-genetic factors (e.g., age, mental health) have been shown to impact the effectiveness of plasticity between individuals,^78^ with varying levels of supraspinal compensation between patients in part explaining why the degree of spinal cord compression does not always correlate with severity of symptoms.^79^ An alternative hypothesis suggests that compensatory brain changes may occur due to consequent loss of inhibitory input from damaged regions. For example, following ‘pathological change’ in the primary visual cortex, it can be envisioned that there is reduced physiological inhibition with connected structures, such as the cerebellum, which would be interpreted as ‘compensatory change’ on MRI.^80^ This is consistent with FC analysis in patients with primary open-angle glaucoma, where postretinal neural atrophy of the primary visual cortex led to a reported increased FC between the primary visual cortex and cerebellum.^81^ Whilst the ‘compensatory change’ mechanism provides a clearer explanation as to why significant spinal cord compression can present with only mild deficits,^79^ the two proposed mechanisms of adaptation and loss of inhibition are likely not mutually exclusive.

Reorganisation reported within the cerebellum of patients with DCM is consistent with studies in paraplegic patients.^82^ Given the cerebellum is thought to play an important role in motor control and visual processing, reorganisation within the cerebellum may help compensate for motor and visual deficits.^83^ Importantly, one would hypothesize that ‘compensatory changes’ are a finite resource: as myelopathy worsens, the compensatory ability becomes exhausted,^12^ and ‘compensatory changes’ in subcortical areas attempt to sustain and optimise residual function. Extending this idea, it could be that recruitment of brain regions less functionally related to the damaged area is an indicator that the degree of compensation has become less effective.

Importantly, neural plasticity and ‘compensatory changes’ may not always be completely beneficial (‘adaptive’) to the patient, due to the finite nature of the brain as a resource. This is consistent with white matter network analyses in patients with developmental dyslexia, where increased connectivity in the fusiform gyrus was associated with poorer reading accuracy,^84^ thereby providing support for the notion that ‘compensatory changes’ can be ‘maladaptive’. This idea of ‘maladaptive compensatory changes’ can be extended to DCM, where, for example, increased demand on the visual network to compensate for reduced proprioceptive input in postural control limits the visual network's ability to adequately meet its own physiological demands,^85^ leading to reported deficits such as blurred vision.^13^ Therefore, whilst we suggest that ‘compensatory changes’ are a physiological response with the goal of preserving function, the reality is that this may not always be achieved.

Halted deterioration, and some, albeit limited, recovery, has been widely reported following surgery in both animal models of cervical cord compression and in patients with DCM.^86^^,^^87^ Decompression may restore spinal cord integrity, enabling cervical cord neurons to restore old and build new ascending and descending connections. A reduction in cervical cord compression likely reverses brain ‘pathological changes’ that contributed to symptoms and reduces the dependence on brain ‘compensatory changes’ that developed prior to surgery. This would explain the normalisation of brain changes in patients with DCM towards HCs following surgery in multiple studies. Consistent with this, a separate study reported that the proprioceptive system of patients with DCM relied less heavily on compensatory input from the visual and vestibular systems following surgery, and an overall improvement was observed in postural control.^85^

Whilst decompression of the spinal cord itself contributes to recovery in patients with DCM, plasticity in the brain also likely plays an important role, with further brain ‘compensatory changes’ counteracting the reduced functionality of the spinal cord and brain relative to HCs. Therapies designed to promote brain plasticity, such as brain stimulation, cell therapy and brain-computer interfaces, would likely be beneficial to patients with DCM both before and after surgery. The application of such therapies in DCM have yet to be investigated and may provide a novel approach to the care of said patients. Extending this notion to our hypothesis, it could be that investigation of ‘pathological changes’ informs the development of regenerative therapies, whilst ‘compensatory changes’ inform the development of plasticity-enhancing therapies in DCM.

Moreover, there has been increasing interest in reliable prognostic prediction regarding decompression, both to inform clinical trial interpretation, but also clinically, where the risks of surgery need to be balanced against the benefits of it. Current prediction models utilise age, smoking, duration and severity of myelopathy,^88^ with limited additional gains provided by imaging.^89^ Our results suggest that implementing brain MRI analysis may yield dividends. Most included studies reported associations between brain changes on MRI and clinical measures. This provides further evidence for our previous conclusion that brain changes contribute to symptoms in DCM. Brain changes were most commonly associated with the JOA score, which is the most commonly used metric to assess spinal cord function in DCM.^90^ However, findings are currently inconsistent, with several studies reporting no associations. Experience in neuroimaging for traumatic SCI, where brain MRI now forms part of the recommended pipeline for clinical research, would support this potential,^11^ indicating further work is needed, including standardisation of procedures and identification of key regions of interest, as opposed to a false dawn.

This review represents a qualitative analysis of the literature. Substantial heterogeneity between study designs including MRI pre-processing and type of data analyses used (i.e., whole-brain versus region of interest), did not permit an analysis of numerical effect estimates beyond study characteristics. Region of interest analysis, although having a greater statistical power due to reduced risk of type I errors compared to whole-brain analysis, results in a selection bias within the results, as findings are only reported for areas located within the region of interest. Despite this heterogeneity, Fig. 5, Fig. 6, Fig. 7, Fig. 8, Fig. 9 provide a summary of our results, including net change. Whilst we believe our net change comparison provides a useful visual graphic for the trend in directionality (increased or decreased) of specific brain regions across included studies of a similar MRI technique (i.e., structural or functional), it is limited by the fact that we are comparing studies that would not necessarily be measuring the same MRI outcome using the same methodology. As such, we have limited our interpretation to purely highlight trends, and acknowledge that beyond that, no detailed interpretation should be made. Whilst a detailed assessment of MRI methodology (e.g., pre-processing) was beyond the scope of this article, it is important to be wary of how they may have potentially impacted our findings. For example, use of multiple types of MRI scanner across a protocol, as was the case in at least six included studies, could result in the introduction of false positives.

The heterogeneity also extends to patient disease and treatment characteristics. Whilst most included studies were deemed to identify and, to a lesser extent, deal with confounding factors, there was significant variation in the degree to which this was effectively done. The most effective papers removed potential bias from other neurological disorders (e.g., stroke) in their exclusion criteria and conducted a multivariate regression analysis with most if not all of age, sex, handedness and disease duration as covariates. The advent of a minimum dataset for DCM (AO Spine RECODE-DCM) should help circumvent this in the future, by ensuring key determinants of disease are recorded.^91^

Furthermore, the findings are limited by the small sample size used across included studies, the relative paucity of longitudinal studies and their length of follow up, and the limited phenotyping. The median sample size of patients with DCM in included studies was 28, with most follow-up less than 6 months. Whilst risk of bias for included studies was deemed low to moderate, it is important to acknowledge that the JBI criteria does not factor in the sample size (although it does factor in the length of follow up for cohort studies). The risk of bias scores for individual studies should therefore be interpreted in the context of their sample size, with smaller sample sizes indicating an additional source of bias. Regarding length of follow up, experience from traumatic SCI has shown meaningful insights can come years after injury and treatment.^92^ Moreover the outcome measures (e.g., mJOA) used are limited in their discrimination of deficits, even for example in the distinction of left versus right.^93^ Ultimately sample size and power, as well as extended follow-up, are common challenges for neuroimaging studies, which remain labour intensive and potentially costly, particularly when they deviate from standard of care. So in short, although not unusual for neuroimaging research, these represent limitations and a potential source of bias, which must be resolved in future investigations.

Moreover, a proportion of the included studies in our review are attributable to a small number of specific research groups. Whilst this is to be expected in an emerging field of research, and encouragingly many of the findings were replicated across groups, the field would benefit from investigation from a wider research community, which would enhance the potential to discover novel insights. Perhaps in part due to the heavy reliance of fMRI by a few specific research groups, structural MRI of the brain was underrepresented in our review. Further microstructural investigation in the future would be beneficial as it may provide novel insights into alterations in brain structure associated with DCM.

Finally, it is important to acknowledge that our proposed distinction of ‘pathological’ and ‘compensatory’ brain changes in DCM remains a hypothesis at this stage, although the evidence for brain changes in related fields, such as SCI, is now well-established.^67^^,^^68^ We believe that this working hypothesis provides a useful direction for the field as distinguishing between brain changes related directly to spinal cord injury from those as a result of adaptation may have differing implications for prognostication and guiding the development of novel therapies (e.g., regenerative strategies for ‘pathological changes’ versus plasticity-enhancing therapies for ‘compensatory changes’). Importantly, more evidence in the form of preclinical studies and MRI studies *in vivo* are now required to confirm (or refute) our hypothesis. In addition to the greater sample size and follow-up length of MRI studies, standardisation of methodologies (including scanners, sequences, pre-processing and analysis) as well as shared datasets will be hugely beneficial in overcoming current limitations in the field of DCM neuroimaging and will enable a more robust investigation of our ‘pathological’ and ‘compensatory’ brain changes hypothesis.

In conclusion, the literature provides mounting evidence that brain alterations occur in DCM, respond to surgical treatment and may relate to clinical symptoms. We would propose that brain changes in DCM can be categorised into: (1) ‘pathological changes' that contribute to symptoms, and (2) 'compensatory changes' that attempt to preserve function. Pathological and compensatory changes are not necessarily mutually exclusive, but may help to distinguish changes directly related to the spinal cord injury as opposed to those indicative of adaptation, which is useful as they may have differing implications for prognostication and guiding the development of novel therapies. Whilst we have identified key structures and pathways that are altered in DCM, there remains a substantial degree of uncertainty regarding them, including their directionality, but overall the aggregated findings illustrate the exciting potential for brain MRIs in research and perhaps clinical care.

## Contributors

ARF, ODM and BMD contributed to the conception and design of the study. ARF, ODM and KSL contributed to development of the search strategy. Database search outputs were screened by ARF, ODM, MY, SM, KSL, AB and MK. Data was extracted by ARF, ODM, MY and SM. An assessment of risk of bias of the included studies was performed by ARF, MY and SM. ARF completed the literature review, collated the data and performed the data analysis, interpreted the results and wrote the first draft of the manuscript. ODM and BMD accessed and verified the underlying data analysis and assisted in the interpretation of the results. ODM, MRK, VJFN, EAS and BMD critically reviewed the manuscript and provided guidance in the writing of the manuscript. All authors read and approved the final version of the manuscript.

## Data sharing statement

The data collected for this study can be provided upon reasonable request to the corresponding author (oliver.mowforth@nhs.net).

## Declaration of interests

We declare no competing interests.
