# Supplementary material for: Brain MRI changes in degenerative cervical myelopathy: a systematic review
Source: eBioMedicine. 2023 Dec 19;99:104915. doi: 10.1016/j.ebiom.2023.104915 (PMC10772405; doi:10.1016/j.ebiom.2023.104915)
Supplement: Supplementary Data 1–10 [file mmc1.docx]

**Supplementary Data 1 – PRISMA Checklist**

| **Section and Topic** | **Item #** | **Checklist item** | **Location where item is reported** |
| --- | --- | --- | --- |
| **TITLE** | | |  |
| Title | 1 | Identify the report as a systematic review. | Title Page |
| **ABSTRACT** | | |  |
| Abstract | 2 | See the PRISMA 2020 for Abstracts checklist. | Summary – Pg 1 |
| **INTRODUCTION** | | |  |
| Rationale | 3 | Describe the rationale for the review in the context of existing knowledge. | Introduction – Pg 4-5 |
| Objectives | 4 | Provide an explicit statement of the objective(s) or question(s) the review addresses. | Introduction – Pg 5 |
| **METHODS** | | |  |
| Eligibility criteria | 5 | Specify the inclusion and exclusion criteria for the review and how studies were grouped for the syntheses. | Methods – Pg 6-7 |
| Information sources | 6 | Specify all databases, registers, websites, organisations, reference lists and other sources searched or consulted to identify studies. Specify the date when each source was last searched or consulted. | Methods – Pg 6 |
| Search strategy | 7 | Present the full search strategies for all databases, registers and websites, including any filters and limits used. | Supplementary Data 2 |
| Selection process | 8 | Specify the methods used to decide whether a study met the inclusion criteria of the review, including how many reviewers screened each record and each report retrieved, whether they worked independently, and if applicable, details of automation tools used in the process. | Methods – Pg 6 |
| Data collection process | 9 | Specify the methods used to collect data from reports, including how many reviewers collected data from each report, whether they worked independently, any processes for obtaining or confirming data from study investigators, and if applicable, details of automation tools used in the process. | Methods – Pg 6-7 |
| Data items | 10a | List and define all outcomes for which data were sought. Specify whether all results that were compatible with each outcome domain in each study were sought (e.g. for all measures, time points, analyses), and if not, the methods used to decide which results to collect. | Methods – Pg 6 |
|  | 10b | List and define all other variables for which data were sought (e.g. participant and intervention characteristics, funding sources). Describe any assumptions made about any missing or unclear information. | Methods – Pg 6 |
| Study risk of bias assessment | 11 | Specify the methods used to assess risk of bias in the included studies, including details of the tool(s) used, how many reviewers assessed each study and whether they worked independently, and if applicable, details of automation tools used in the process. | Methods – Pg 6 |
| Effect measures | 12 | Specify for each outcome the effect measure(s) (e.g. risk ratio, mean difference) used in the synthesis or presentation of results. | Methods – Pg 7 |
| Synthesis methods | 13a | Describe the processes used to decide which studies were eligible for each synthesis (e.g. tabulating the study intervention characteristics and comparing against the planned groups for each synthesis (item #5)). | Methods – Pg 7-8 |
|  | 13b | Describe any methods required to prepare the data for presentation or synthesis, such as handling of missing summary statistics, or data conversions. | N/A |
|  | 13c | Describe any methods used to tabulate or visually display results of individual studies and syntheses. | Methods – Pg 7-8  Supplementary Data 4 |
|  | 13d | Describe any methods used to synthesize results and provide a rationale for the choice(s). If meta-analysis was performed, describe the model(s), method(s) to identify the presence and extent of statistical heterogeneity, and software package(s) used. | Methods – Pg 7 |
|  | 13e | Describe any methods used to explore possible causes of heterogeneity among study results (e.g. subgroup analysis, meta-regression). | N/A |
|  | 13f | Describe any sensitivity analyses conducted to assess robustness of the synthesized results. | N/A |
| Reporting bias assessment | 14 | Describe any methods used to assess risk of bias due to missing results in a synthesis (arising from reporting biases). | Methods – Pg 7  Supplementary Data 5 |
| Certainty assessment | 15 | Describe any methods used to assess certainty (or confidence) in the body of evidence for an outcome. | N/A |
| **RESULTS** | | |  |
| Study selection | 16a | Describe the results of the search and selection process, from the number of records identified in the search to the number of studies included in the review, ideally using a flow diagram. | Results – Pg 9 |
|  | 16b | Cite studies that might appear to meet the inclusion criteria, but which were excluded, and explain why they were excluded. | Results – Pg 9 |
| Study characteristics | 17 | Cite each included study and present its characteristics. | Results – Figures 2-4,  Supplementary Data 6 |
| Risk of bias in studies | 18 | Present assessments of risk of bias for each included study. | Results – Pg 9-10  Supplementary Data 3 |
| Results of individual studies | 19 | For all outcomes, present, for each study: (a) summary statistics for each group (where appropriate) and (b) an effect estimate and its precision (e.g. confidence/credible interval), ideally using structured tables or plots. | Supplementary Data 6-12  Results – Table 3-4 |
| Results of syntheses | 20a | For each synthesis, briefly summarise the characteristics and risk of bias among contributing studies. | Results – Pg 9  Supplementary Data 3, 5, 6 |
|  | 20b | Present results of all statistical syntheses conducted. If meta-analysis was done, present for each the summary estimate and its precision (e.g. confidence/credible interval) and measures of statistical heterogeneity. If comparing groups, describe the direction of the effect. | N/A |
|  | 20c | Present results of all investigations of possible causes of heterogeneity among study results. | N/A |
|  | 20d | Present results of all sensitivity analyses conducted to assess the robustness of the synthesized results. | N/A |
| Reporting biases | 21 | Present assessments of risk of bias due to missing results (arising from reporting biases) for each synthesis assessed. | Supplementary Data 3 and 5 |
| Certainty of evidence | 22 | Present assessments of certainty (or confidence) in the body of evidence for each outcome assessed. | N/A |
| **DISCUSSION** | | |  |
| Discussion | 23a | Provide a general interpretation of the results in the context of other evidence. | Discussion – Pg 13-16 |
|  | 23b | Discuss any limitations of the evidence included in the review. | Discussion – Pg 16-18 |
|  | 23c | Discuss any limitations of the review processes used. | Discussion – Pg 16-18 |
|  | 23d | Discuss implications of the results for practice, policy, and future research. | Discussion – 13-18 |
| **OTHER INFORMATION** | | |  |
| Registration and protocol | 24a | Provide registration information for the review, including register name and registration number, or state that the review was not registered. | Methods – Pg 6 |
|  | 24b | Indicate where the review protocol can be accessed, or state that a protocol was not prepared. | Methods – Pg 6 |
|  | 24c | Describe and explain any amendments to information provided at registration or in the protocol. | N/A |
| Support | 25 | Describe sources of financial or non-financial support for the review, and the role of the funders or sponsors in the review. | Summary – Pg 2 |
| Competing interests | 26 | Declare any competing interests of review authors. | Pg 19 |
| Availability of data, code and other materials | 27 | Report which of the following are publicly available and where they can be found: template data collection forms; data extracted from included studies; data used for all analyses; analytic code; any other materials used in the review. | Pg 19 |

**Supplementary Data 2 – Search Strategy**

Full search phrases used for Medline and Embase on 28^th^ August 2023

| **Ovid MEDLINE** | | | 767 articles |
| --- | --- | --- | --- |
| Cervical spine concept | | | |
| 1 | exp Cervical Vertebrae/ or exp Cervical Cord/ or cervical.tw | | |
| DCM concept | | | |
| 2 | Exp Spinal Cord Diseases/ or Exp Spinal Diseases/ | | |
| 3 | degenerat*.tw | | |
| 4 | 2 and 3 | | |
| 5 | Myelopath*.tw | | |
| 6 | Myeloradiculopath*.tw | | |
| 7 | Radiculopath*.tw | | |
| 8 | Exp Spinal Cord Compression/ | | |
| 9 | Exp “Ossification of the Posterior Longitudinal Ligament”/ | | |
| 10 | Ossification of the Posterior Longitudinal Ligament.tw | | |
| 11 | OPLL.tw | | |
| 12 | Exp Spinal Stenosis/ | | |
| 13 | Cervical stenosis.tw | | |
| 14 | Exp Spondylosis/ | | |
| 15 | Spondylosis.tw | | |
| 16 | Spondylotic.tw | | |
| 17 | Degenerative cervical myelopathy.tw | | |
| 18 | DCM.tw | | |
| 19 | Cervical spondylotic myelopathy.tw | | |
| 20 | CSM.tw | | |
| 21 | 4 or 5 or 6 or 7 or 8 or 9 or 10 or 11 or 12 or 13 or 14 or 15 or 16 or 17 or 18 or 19 or 20 | | |
| 22 | 1 and 21 | | |
| MRI concept | | | |
| 23 | Exp Magnetic Resonance Imaging/ or MRI.tw | | |
| 24 | (functional MRI or functional magnetic resonance imaging).tw. | | |
| 25 | (Resting state fMRI or resting-state fMRI or rsfMRI or rs-fMRI or resting state).tw. | | |
| 26 | Exp Neural pathways/ or Exp Brain mapping/ | | |
| 27 | (Connectivity or functional connectivity or structural connectivity).tw. | | |
| 28 | Exp Neuronal Plasticity/ or plasticity.tw or cerebral reorganization.tw or cerebral reorganisation.tw | | |
| 29 | 23 or 24 or 25 or 26 or 27 or 28 | | |
| Brain concept | | | |
| 30 | Exp Brain/ or exp Cerebrum/ or exp Cerebellum/ or exp Cerebral Cortex/ or exp Thalamus/ or exp Frontal Lobe/ or exp Occipital Lobe/ or exp Parietal Lobe/ or exp Temporal Lobe/ or exp Basal Ganglia/ or exp Internal Capsule/ or exp Gray Matter/ or exp White Matter/ or exp Corpus callosum/ | | |
| 31 | (Brain or cerebr* or cerebell* or cerebral cortex or cerebral cortices or cortical* or thalamus or lobe* or gyrus or basal ganglia or internal capsule or corpus callosum or corona radiata or visual-cerebellum or sensorimotor or default-mode network or default mode network or cuneus).tw. | | |
| 32 | 30 or 31 | | |
| Combined concepts | | | |
| 33 | 22 and 29 and 32 | | |
| **EMBASE** | | 1743 articles | |
| Cervical spine concept | | | |
| 1 | exp Cervical Vertebra/ or cervical spine/ or exp Cervical spinal cord/ or cervical.tw | | |
| DCM concept | | | |
| 2 | Exp Spinal Cord Disease/ or Exp Spine Disease/ | | |
| 3 | Exp degeneration/ | | |
| 4 | degenerat*.tw | | |
| 5 | 3 or 4 | | |
| 6 | 2 and 5 | | |
| 7 | Myelopath*.tw | | |
| 8 | Myeloradiculopath*.tw | | |
| 9 | Exp radiculopathy/ | | |
| 10 | Radiculopath*.tw | | |
| 11 | Exp Spinal Cord Compression/ | | |
| 12 | Exp Posterior Longitudinal Ligament/ and exp ossification/ | | |
| 13 | Ossification of the Posterior Longitudinal Ligament.tw | | |
| 14 | OPLL.tw | | |
| 15 | Exp vertebral canal stenosis/ | | |
| 16 | Cervical stenosis.tw | | |
| 17 | Exp Cervical Spondylosis/ | | |
| 18 | Exp Spondylosis/ | | |
| 19 | Spondylosis.tw | | |
| 20 | Spondylotic.tw | | |
| 21 | Exp Cervical myelopathy/ | | |
| 22 | Degenerative cervical myelopathy.tw | | |
| 23 | DCM.tw | | |
| 24 | Exp Cervical spondylotic myelopathy/ | | |
| 25 | Cervical spondylotic myelopathy.tw | | |
| 26 | CSM.tw | | |
| 27 | 6 or 7 or 8 or 9 or 10 or 11 or 12 or 13 or 14 or 15 or 16 or 17 or 18 or 19 or 20 or 21 or 22 or 23 or 24 or 25 or 26 | | |
| 28 | 1 and 27 | | |
| MRI concept | | | |
| 29 | (magnetic resonance imaging or MRI).tw. | | |
| 30 | Exp functional magnetic resonance imaging/ or functional MRI.tw or functional magnetic resonance imaging.tw | | |
| 31 | (Resting state fMRI or resting-state fMRI or rsfMRI or rs-fMRI or resting state).tw. | | |
| 32 | Exp brain mapping/ | | |
| 33 | Exp functional connectivity/ or connectivity.tw or functional connectivity.tw or structural connectivity.tw. | | |
| 34 | Exp nerve cell plasticity/ or plasticity.tw or cerebral reorganization.tw or cerebral reorganisation.tw | | |
| 35 | 29 or 30 or 31 or 32 or 33 or 34 | | |
| Brain concept | | | |
| 36 | Exp brain/ or exp cerebellum/ or exp brain cortex/ or exp thalamus/ or exp frontal lobe/ or exp occipital lobe/ or exp parietal lobe/ or exp temporal lobe/ or exp capsula interna / or exp gray matter/ or exp white matter/ or exp corpus callosum/ | | |
| 37 | (Brain or cerebr* or cerebell* or cerebral cortex or cerebral cortices or cortical* or thalamus or lobe* or gyrus or basal ganglia or internal capsule or corpus callosum or corona radiata or visual-cerebellum or sensorimotor or default-mode network or default mode network or cuneus).tw. | | |
| 38 | 36 or 37 | | |
| Combined concepts | | | |
| 39 | 28 and 35 and 38 | | |

**Supplementary Data 3 – Study Characteristics**

**Supplementary Data 3. Summary of study characteristics of included studies**. Missing information and incorrectly reported information are reported as NR (not reported) and IR (incorrectly reported), respectively. 17/44 studies did not assess post-surgical DCM patients (represented by ‘—’ under ‘Type of Decompression’ column).

*ADC = apparent diffusion coefficient; ALFF = amplitude of low-frequency fluctuations; ALS = amyotrophic lateral sclerosis; ASL = arterial spin labelling; CBF = cerebral blood flow; dALFF = dynamic ALFF; DCM = degenerative cervical myelopathy; DWI = diffusion-weighted imaging; EC = effective connectivity; FA = fractional anisotropy; FC = functional connectivity; FCS = FC strength; FLAIR = fluid attenuated inversion recovery; GFA = global FA; gFCD = global FC density; GMV = grey matter volume; HCs = healthy controls; MR Spec = MR spectroscopy; NQA = normalized quantitative anisotropy; ReHo = regional homogeneity; sALFF = static ALFF; SC = structural connectivity; VOA = volume of activation; WMV = white matter volume.*

^a^ Good/Poor recovery = recovery rate in visual analog scale (VAS) score more/less than 50%.

^b^ Mild and Moderate/Severe DCM were defined as mJOA 15-17 and mJOA ≤ 14, respectively.

^c^ Mild and Moderate DCM were defined as mJOA > 12 and mJOA 9-12, respectively.

^d^ Results are based on analysis of non-matched group of 11 DCM patients pre- and post-surgically.

| **Reference** | **Sample Size (% of M)** | **Mean Age (± SD)** | **Type of Decompression Surgery (if applicable)** | **MRI Scanner** | **Imaging Modality and Timing** | **Outcomes Assessed** | **Type of Analysis** |
| --- | --- | --- | --- | --- | --- | --- | --- |
| Liu et al, 2021 [19] | DCM: 45 (37.8), 41 after surgery  HCs: 45 (37.8) | DCM: 51.2 ± 7.0  HCs: 50.5 ± 5.0 | NR | 3.0T Trio, Siemens | T1 at: baseline and 6 months after surgery | GMV | VBM  • One-sample t-test for within-group analysis (p < 0.05, FDR corrected)  • ANCOVA for between-group analysis with Bonferroni correction (voxel p < 0.01, cluster p < 0.001) |
| Jütten et al, 2021 [20] | DCM: 43 (62.8), 21 after surgery  HCs: 20 (55.0) | DCM: 61.0 ± 11.0  HCs: 64.0 ± 6.0 | NR | 3.0T Prisma, Siemens | T1 at: baseline and 3 months after surgery | GMV/WMV | ROI  • ANCOVA for between-group analysis with Bonferroni correction (p < 0.05) |
| Chen et al, 2018 [21] | DCM: 27 (55.6)  HCs: 11 (54.5) | DCM: 57.9 ± 9.1  HCs: 54.8 ± 8.4 | NR | 3.0T Discovery MR750, General Electric | T1 and rs-fMRI at: baseline only | GMV/WMV, ALFF, ReHo, FC | VBM (GMV/WMV)  • Two-sample t-test for between-group analysis (p < 0.05)  Whole-Brain (ALFF/ReHo)  • One-sample t-test for within-group analysis (p < 0.001, cluster FWE correction p < 0.05)  • Two-sample t-test for between-group analysis (p < 0.001, cluster FWE correction p < 0.05)  ROI (FC)  • Two-sample t-test for between-group analysis (p < 0.001, cluster FWE correction p < 0.05) |
| Wang et al, 2018 [22] | DCM: 30 (63.3)  HCs: 25 (44.0) | DCM: 51.3 ± 7.5  HCs: 51.2 ± 7.7 | — | 3.0T Ingenia, Philips | T1 at: baseline only | GMV | ROI (GMV)  • Two-sample t-test for between-group analysis (voxel p < 0.01, cluster p < 0.05) |
| Zhou et al, 2022 [23] | DCM: 35 (51.4)  HCs: 35 (51.4) | DCM: 54.3 ± 6.3  HCs: 53.9 ± 7.3 | — | 3.0T Discovery MR750, General Electric | T1 and rs-fMRI at: baseline only | GMV, gFCD | VBM (GMV)  • Two-sample t-test for between-group analysis (p < 0.05, FDR corrected)  Whole-Brain (gFCD)  • Two-sample t-test for between-group analysis (p < 0.05, FDR corrected) |
| Wang et al, 2021 [24] | DCM: 19 (73.7)  HCs: 16 (NR) | DCM: 55.2 ± 9.4  HCs: 29.4 ± 11.3 | 8/19 cervical laminectomy and fusion  7/19 laminoplasty  3/19 anterior cervical discectomy and fusion  1/19 anterior cervical corpectomy | 3.0T Prisma, Siemens  3.0T Trio, Siemens | T1 and rs-fMRI at: baseline and 3 months after surgery | Cortical thickness + volume, FC | Whole-Brain (Cortical Thickness/Volume)  • Two-sample t-test for between-group analysis (p < 0.05)  • Paired t-test for intra-group analysis pre- and post-surgery (p < 0.05)  Whole-Brain (FC)  • Two-sample t-test for between-group analysis (p < 0.05)  • Paired t-test for intra-group analysis pre- and post-surgery (p < 0.05)  ROI (FC)  • Two-sample t-test for between-group analysis (p < 0.05)  • Paired t-test for intra-group analysis pre- and post-surgery (p < 0.05) |
| Filimonova et al, 2023 [25] | DCM: 47 (60.0)  Cervical radiculopathy: 25 (52.0) | DCM: 56.8 ± 8.8  Cervical radiculopathy: 46.4 ± 8.1 | — | 3.0T Ingenia, Philips | T1 at: baseline only | Volume | ROI  • ANCOVA for between-group analysis (p < 0.05 FDR corrected) |
| Aleksanderek et al, 2017 [26] | DCM: 28 (75.0)  HCs: 10 (50.0) | DCM: 51.0 ± 2.0  HCs: 48.0 ± 4.0 | 19/28 1-level decompression  9/28 2-level decompression | 3.0T Trio, Siemens | MR spectroscopy and DW-MRI at: baseline and 6 months after surgery | FA and mean diffusivity of white matter, metabolite concentrations | ROI  • Two sample t-test for between-group analysis (p < 0.05)  • Paired t-test for intra-group analysis pre- and post-surgery (p < 0.05) |
| Wang et al, 2022 [27] | DCM: 22 (81.8) | DCM: 56.9 ± 9.0 | 10/22 cervical laminectomy and fusion  9/22 laminoplasty  2/22 anterior cervical discectomy and fusion  1/22 anterior cervical corpectomy | 3.0T Prisma, Siemens | DW-MRI at: baseline and 3 months after surgery | SC, FA, GFA, NQA | ROI  • Two-sample t-test for between-group analysis (p < 0.05, FDR corrected) |
| Koike et al, 2015 [28] | DCM: 16 (NR)  ALS w/o DCM: 19 (57.9)  HCs: 11 (NR) | DCM: 64.5 ± 13.2  ALS w/o DCM: 68.0 ± 8.5  HCs: 62.5 ± 15.3 | — | 3.0T Verio, Siemens | DW-MRI at: baseline only | ADC | ROI  • ANOVA for between-group analysis with Bonferroni correction (p < 0.05) |
| Hoshimaru et al, 2010 [29] | DCM: 10 (30.0) | DCM: 65.2 (NR) | 10/10 cervical laminoplasty | NR | FLAIR at: baseline and 3 months after surgery | NR | Whole-Brain  • No statistical analysis mentioned in methods |
| Zhao et al, 2020 [30] | Dataset 1:  DCM: 27 (55.6)  HCs: 11 (54.5)  Dataset 2:  DCM: 26 (53.8)  HCs: 36 (52.8) | Dataset 1:  DCM: 57.9 ± 9.1  HCs: 54.8 ± 8.4  Dataset2:  DCM: 54.7 ± 8.8  HCs: 53.7 ± 8.3 | — | Dataset 1:  3.0T Discovery MR750, General Electric  Dataset 2:  3.0T Prisma, Siemens | rs-fMRI at: baseline only | FC, graph theory, BOLD signal | ROI (FC/Graph Theory)  • Two-sample t-test for between-group analysis (p < 0.001, cluster FWE correction p < 0.05)  Whole-Brain (BOLD Signal)  • Two-sample t-test for between-group analysis (p < 0.001, cluster FWE correction p < 0.05) |
| Sawada et al, 2020 [31] | DCM good recovery: 12 (41.7)  DCM poor recovery: 15 (73.3) | DCM good recovery: 62.7 ± 16.0  DCM poor recovery: 65.5 ± 13.8 | Anterior fusion, or laminoplasty if more than 3 levels of spinal cord compression  6/12 of good recovery group underwent posterior decompression  10/15 of poor recovery group underwent posterior decompression | 3.0T Ingenia, Philips | fMRI at: baseline and 7 days after surgery | FC | ROI  • Two-sample t-test for between-group analysis (voxel p < 0.05, cluster FWE correction p < 0.05)  • Paired t-test for intra-group analysis pre- and post-surgery (voxel p < 0.05, cluster FWE correction p < 0.05)  • Two-sided T-contrast for significant ROI-to-ROI connectivity (p < 0.05, FDR corrected) |
| Peng et al, 2020 [32] | DCM: 43 (62.8), 21 after surgery  HCs: 43 (62.8) | DCM: 49.1 ± 6.7  HCs were age-matched | NR | 3.0T Siemens (specifics NR) | rs-fMRI at: baseline and 3 months after surgery | FC | ROI  • Two-sample t-test for between-group analysis (p < 0.05)  • Paired t-test for intra-group analysis pre- and post-surgery (p < 0.05) |
| Chen et al, 2020 [33] | DCM: 30 (53.3), 14 (64.3) after surgery  HCs: 20 (50.0) | DCM: 58.3 ± 8.8, 58.6 ± 10.2 after surgery  HCs: 58.7 ± 8.3 | 18/30 anterior decompression  12/30 posterior decompression | 3.0T Discovery MR750, General Electric | rs-fMRI at: baseline and 3 months after surgery | FC | ROI  • Two-sample t-test for between-group analysis (p < 0.001, cluster FWE correction p < 0.05)  • Paired t-test for significant ROI-wise FC analysis (p < 0.05) |
| Zhou et al, 2015 [34] | DCM: 31 (71.0)  HCs: 31 (71.0) | DCM: 51.4 ± 6.3  HCs: 50.9 ± 6.3 | — | 3.0T Trio, Siemens | rs-fMRI at: baseline only | FC, FCS | ROI  • ANCOVA followed by post-hoc two-sample t-test with Monte Carlo correction (p < 0.05) |
| Zhou et al, 2015 [35] | DCM: 17 (52.9)  HCs: 17 (47.1) | DCM: 50.5 ± 7.3  HCs: 50.3 ± 7.3 | — | 3.0T Trio, Siemens | rs-fMRI at: baseline only | FC | ROI  • Two-sample t-test for between-group analysis with Monte Carlo correction (p < 0.05) |
| Wei et al, 2021 [36] | DCM: 27 (IR)  HCs: 24 (50.0) | DCM: 53.7 ± 8.1  HCs: 54.2 ± 7.3 | NR | 3.0T Prisma, Siemens | rs-fMRI and ASL at: baseline only | FC, CBF | Whole-Brain (FC/CBF)  • Two-sample t-test for between-group analysis  ROI (FC/CBF)  • Two-sample t-test for between-group analysis (p < 0.05, FDR corrected) |
| Zhao et al, 2021 [37] | DCM: 27 (55.6)  HCs: 11 (54.5) | DCM: 57.9 ± 9.1  HCs: 54.8 ± 8.4 | NR | 3.0T Discovery MR750, General Electric | rs-fMRI at: baseline only | EC | ROI  • Two-sample t-test for between-group analysis (p < 0.05) |
| Takenaka et al, 2019 [38] | DCM: 28 (50.0), 26 after surgery  HCs: 28 (50.0) | DCM: 66.5 ± 10.9  HCs: 66.5 ± 11.0 | 25/28 cervical laminoplasty  3/28 anterior decompression and fusion | 3.0T Discovery MR750, General Electric | rs-fMRI at: baseline and 6 months after surgery | FC | Whole-Brain  • Two-sample t-test for between-group analysis (p < 0.001, p < 0.000305 FDR and Bonferroni corrected)  • Paired t-test for intra-group analysis pre- and post-surgery (p < 0.001, p < 0.00192 FDR and Bonferroni corrected) |
| Kuang et al, 2019 [39] | DCM: 33 (48.5)  HCs: 33 (45.5) | DCM: 54.8 ± 8.4  HCs: 53.5 ± 8.1 | — | 3.0T Discovery MR750, General Electric | rs-fMRI at: baseline only | FC, ALFF, ReHo | ROI (FC)  • Two-sample t-test for between-group analysis (p < 0.01, FDR corrected)  Whole-Brain (ALFF/ReHo)  • Two-sample t-test for between-group analysis (p < 0.01, FDR corrected) |
| Wang et al, 2022 [40] | Patient cohort: 99 (61.6)  Asymptomatic SCC: 21 (61.9)  Mild DCM: 48 (60.4)  Moderate/Severe DCM: 30 (63.3)  HCs: 17 (52.9) | Patient cohort: 59.0 ± 10.7  Asymptomatic SCC: 57.0 ± 14.2  Mild DCM: 60.3 ± 10.3  Moderate/Severe DCM: 58.4 ± 8.6  HCs: 41.0 ± 14.0 | — | 3.0T Prisma, Siemens | rs-fMRI at baseline only | FC, graph theory | Whole-Brain (FC/Graph Theory)  • Two-sample t-test for between-group analysis (p < 0.05)  ROI (FC/Graph Theory)  • Two-sample t-test for between-group analysis (p < 0.05) |
| Zhao et al, 2022 [41] | DCM: 88 (51.1)  HCs: 77 (50.6) | DCM: 49.2 ± 7.9  HCs: 45.1 ± 8.8 | — | 3.0T Trio, Siemens | rs-fMRI at: baseline only | FC. graph theory | Whole-Brain  • Two-sample t-test for between-group analysis (p < 0.05) |
| Su et al, 2021 [42] | Dataset 1:  DCM: 27 (55.6)  HCs: 11 (54.5)  Dataset 2:  DCM: 26 (53.8)  HCs: 36 (52.8) | Dataset 1:  DCM: 57.9 ± 9.1  HCs: 54.8 ± 8.4  Dataset 2:  DCM: 54.7 ± 8.8  HCs: 53.7 ± 8.3 | NR | Dataset 1:  3.0T Discovery MR750, General Electric  Dataset 2:  3.0T Prisma, Siemens | rs-fMRI at: baseline only | FC | Whole-Brain  • Two-sample t-test for between-group analysis (p < 0.05, FDR corrected) |
| Eto et al, 2022 [43] | DCM: 15 (80.0) | DCM: 65.3 ± 11.3 | 9/15 anterior decompression and fusion  4/15 posterior decompression and fusion  2/15 laminoplasty | 3.0T Achieva, Philips | rs-fMRI at: baseline and 6 months after surgery | FC | Whole-Brain  • Paired-test for intra-group pre- and post-surgery analysis (p < 0.001, p < 0.05 FDR corrected) |
| Wu et al, 2023 [44] | Total DCM: 32 (50.0)  DCM w/ normal gait pattern: 16 (50.0)  DCM w/ abnormal gait pattern: 16 (50.0)  HCs: 16 (50.0) | Total DCM: 52.47  DCM w/ normal gait pattern: 51.38 ± 6.91  DCM w/ abnormal gait pattern: 53.56 ± 9.51  HCs: 52.63 ± 9.51 | — | 3.0T Discovery MR750, General Electric | rs-fMRI at: baseline only | FC  ALFF | ROI (FC)  • ANOVA for between-group analysis (p < 0.001 corrected with FWE correction at cluster level)  Whole-Brain (ALFF)  • ANOVA with Bonferroni post-hoc test for between-group analysis (p < 0.001 corrected with FWE correction at cluster level) |
| Zhao et al, 2022 [45] | Total DCM: 66  DCM w/ depression: 33 (51.5)  DCM  DCM w/o depression: 33 (sex-matched)  HCs: 33 (51.5) | DCM w/ depression: 55.2 ± 7.28  DCM w/o depression: age-matched  HCs: 55.1 ± 6.41 | NR | 3.0T Prisma, Siemens | rs-fMRI at: baseline only | FC | Whole-Brain, ROI  • Two-sample t-test for between-group analysis (p < 0.001, p < 0.05 FWE corrected) |
| Hrabálek et al, 2018 [46] | DCM: 20 (45.0) | DCM: 56.6 (NR) | 17/20 one-level anterior decompression  3/20 two-level anterior decompression | 1.5T Avanto, Siemens  1.5T Symphony, Siemens | fMRI at: baseline, 6 months and 12 months after surgery | Activation during active wrist flexion and extension | ROI  • ANOVA for between-group analysis (p < 0.05 corrected)  • Post hoc group contrasts for intra-group analysis pre and post-surgery (p < 0.05 corrected) |
| Ryan et al, 2018 [47] | DCM: 22 (86.4)  HCs: 10 (70.0) | DCM: 50.0 ± 10.9  HCs: 48.0 ± 9.9 | NR | 3.0T Trio, Siemens | fMRI at: baseline, 6 weeks and 6 months after surgery | VOA during finger tapping, BOLD signal | ROI  • Two-sample t-test for between-group analysis (p < 0.05)  • Paired t-test for intra-group analysis (p < 0.05) |
| Sawada et al, 2018 [48] | DCM: 6 (50.0)  HCs: 5 (100.0) | DCM: 48.0 ± 12.0  HCs: 39.0 ± 12.0 | 4/6 one-level anterior discectomy (anterior fusion)  2/6 two-level anterior discectomy and partial corpectomy (anterior fusion) | 3.0T Ingenia, Philips | fMRI at: baseline and 7 days after surgery | Activation during finger tapping | Whole-Brain  • Two-sample t-test for between-group analysis (p < 0.005, cluster FWE correction p < 0.05)  • Paired t-test for intra-group analysis pre- and post-surgery (voxel p < 0.01, cluster FWE correction p < 0.05)  ROI  • Two-sample t-test for between-group analysis (p < 0.005, cluster FWE correction p < 0.05)  • Paired t-test for intra-group analysis pre- and post-surgery (voxel p < 0.01, cluster FWE correction p < 0.05) |
| Aleksanderek, et al 2017 [49] | Total DCM: 28 (75.0)  Mild DCM: 15 (86.7)  Moderate DCM: 13 (61.5)  HCs: 10 (50.0) | Mild DCM: 50.1 ± 12.0  Moderate DCM: 53.0 ± 9.0  HCs: 48.0 ± 12.0 | 12/15 of mild and 7/13 of moderate DCM 1-level decompression  3/15 of mild and 6/13 of moderate DCM 2-level decompression | 3.0T Trio, Siemens | fMRI and MR spectroscopy at: baseline and 6 months after surgery | VOA during finger tapping, metabolite concentrations | ROI  • Two-sample t-test for between-group analysis (p < 0.05, FDR corrected)  • Paired t-test for intra-group analysis (p < 0.05, FDR corrected) |
| Cronin et al, 2021 [50] | DCM: 23 (60.9) | DCM: 65.0 ± 13.0 | — | 3.0T Prisma, Siemens | fMRI at: baseline only | VOA during finger tapping, BOLD signal | ROI  • *Unclear* |
| Bhagavatula et al, 2016 [51] | DCM: 17 (94.1)  HCs: 12 (NR) | DCM: 51.8 ± 10.7  HCs: NR | 12/17 laminectomy  5/17 anterior decompression | 3.0T Philips (specifics NR) | fMRI at: baseline and 6 months after surgery | VOA during finger tapping | Whole-Brain  • General linear model for between-group analysis with Bonferroni correction (p < 0.005) |
| Duggal et al, 2010 [52] | DCM: 12^d^ (75.0)  HCs: 10 (40.0) | DCM: 49.6 ± 12.8  HCs: 41.0 ± 8.8 | NR | 4T Varian | fMRI at: baseline and 6 months after surgery | VOA during finger tapping | ROI  • Paired t-test for between-group analysis (p < 0.05) |
| Dong et al, 2008 [53] | DCM: 8 (50.0)  HCs: 6 (NR) | DCM: 61.1  HCs: NR | 3/8 anterior cervical discectomy and fusion  2/8 cervical laminoplasty and fusion  1/8 anterior corpectomy and fusion  1/8 laminoplasty  1/8 laminectomy | 1.5T Sonata, Siemens | fMRI at: baseline, 3 months and 6 months after surgery | VOA and activation magnitude during wrist extension and 3-finger pinch | ROI  • Two-sample t-test for between-group analysis  • Paired t-test for intra-group analysis pre- and post-surgery |
| Holly et al, 2007 [54] | DCM: 4 (25.0)  HCs: 5 (NR) | DCM: 58.3  HCs were age-matched | 2/4 laminectomy and fusion  1/4 laminoplasty  1/4 anterior cervical discectomy and fusion | 1.5T Sonata, Siemens | fMRI at: baseline and at least 2 of 6 weeks, 3 months or 6 months after surgery | Activation during ankle dorsiflexion or wrist extension | Whole-Brain  • *Unclear*  ROI  • *Unclear* |
| Takenaka et al, 2020 [55] | DCM: 28 (50.0), 26 after surgery  HCs: 28 (50.0) | DCM: 67.0 ± 11.0  HCs: 67.0 ± 11.0 | 25/28 cervical laminoplasty  3/28 anterior decompression and fusion | 3.0T Discovery MR750, General Electric | rs-fMRI at: baseline and 6 months after surgery | ALFF | Whole-Brain  • Two-sample t-test for between-group analysis (p < 0.05)  • Paired t-test for intra-group analysis (p < 0.05) |
| Zhou et al, 2014 [56] | DCM: 19 (57.9)  HCs: 19 (52.6) | DCM: 49.6 ± 7.4  HCs: 49.4 ± 7.2 | — | 3.0T Trio, Siemens | rs-fMRI at: baseline only | ALFF | ROI  • Two-sample t-test for between-group analysis with Monte Carlo correction (p < 0.05) |
| Zhao et al, 2022 [57] | Dataset 1:  DCM: 21 (52.4)  HCs: 11 (54.5)  Dataset 2:  DCM: 33 (48.5)  HCs: 39 (51.3) | Dataset 1:  DCM: 53.3 ± 9.1  HCs: 54.8 ± 8.4  Dataset 2:  DCM: 53.5 ± 11.9  HCs: 53.7 ± 8.3 | — | Dataset 1:  3.0T Discovery MR750, General Electric  Dataset 2:  3.0T Prisma, Siemens | rs-fMRI at: baseline only | ALFF | Whole-Brain  • Two-sample t-test for between-group analysis (p < 0.001, cluster FWE correction p < 0.05)  ROI  • Two-sample t-test for between-group analysis (p < 0.001, cluster FWE correction p < 0.05) |
| Fan et al, 2022 [58] | DCM: 47 (IR)  HCs: 44 (IR) | DCM: 51.3 ± 2.8  HCs: 51.7 ± 3.6 | NR | 3.0T Discovery MR750, General Electric | rs-fMRI at: baseline only | sALFF, dALFF | Whole-Brain  • Two-sample t-test for between-group analysis (p < 0.001, cluster FWE correction p < 0.05)  ROI  • Multivariate pattern analysis (p < 0.05 FWE corrected) |
| Su et al, 2023 [59] | DCM: 62 (50.0)  HCs: 60 (50.0) | DCM: 53.5 ± 7.38  HCs: 53.4 ± 7.47 | NR | 3.0T Prisma, Siemens | rs-fMRI at: baseline only | ALFF | Whole-Brain, ROI  • Two-sample t-test for between-group analysis with Bonferroni correction (p < 0.001, p < 0.05 FWE corrected) |
| Cao et al, 2021 [60] | DCM: 67 (52.2)  HCs: 60 (53.3) | DCM: 47.5 ± 7.4  HCs: 45.5 ± 10.6 | — | 3.0T Trio, Siemens | rs-fMRI at: baseline only | Graph theory | Whole-Brain  • Non-parametric permutation test for between-group analysis (p < 0.05, FDR corrected) |
| Kuang et al, 2020 [61] | DCM: 33 (48.5), 31 included in analysis  HCs: 33 (45.5), 31 included in analysis | DCM: 54.8 ± 8.4  HCs: 53.5 ± 8.1 | — | 3.0T Discovery MR750, General Electric | T1 at: baseline only | Graph theory | Whole-Brain  • Non-parametric permutation test for between-group analysis (p < 0.05) |
| Tan et al, 2015 [62] | DCM: 21 (42.9)  HCs: 21 (sex-matched) | DCM: 48.0 ± 7.0  HCs were age-matched | NR | 3.0T Trio, Siemens | rs-fMRI at: baseline and 3 months after surgery | ReHo | ROI  • Two-sample t-test for between-group analysis (p < 0.05, GRF corrected)  • Paired t-test for intra-group analysis pre- and post-surgery (p < 0.05, GRF corrected) |
| Goncalves et al, 2016 [63] | DCM: 17 (94.1)  HCs: 8 (62.5) | DCM: 52.0 ± 2.0  HCs: 48.0 ± 3.0 | NR | 3.0T Prisma, Siemens  3.0T Trio, Siemens | MR spectroscopy at: baseline, 6 weeks and 6 months after surgery | Metabolite concentrations | ROI  • Two-sample t-test for between-group analysis (p < 0.05)  • Paired t-test for intra-group analysis pre- and post-surgery (p < 0.05) |
| Kowalczyk et al, 2012 [64] | DCM: 24 (66.7)  HCs: 11 (63.6) | DCM: 53.0 ± 2.0  HCs: 46.0 ± 4.0 | — | 3.0T Trio, Siemens | MR spectroscopy at: baseline only | Metabolite concentration | ROI  • Two-sample t-test for between-group analysis with post hoc analysis using Tukey’s test (p < 0.05) |
| Zhou et al, 2018 [65] | DCM: 18 (72.2)  HCs: 18 (72.2) | DCM: 51.9 ± 7.8  HCs: 50.5 ± 7.1 | — | 3.0T Trio, Siemens | pCASL at: baseline only | CBF | Whole-Brain  • Two-sample t-test for between-group analysis with GRF correction (voxel p < 0.01, cluster p < 0.05)  ROI  • Two-sample t-test for between-group analysis with GRF correction (voxel p < 0.01, cluster p < 0.05) |

**Supplementary Data 4 – JBI Critical Appraisal Checklist**

**Cross-sectional studies**


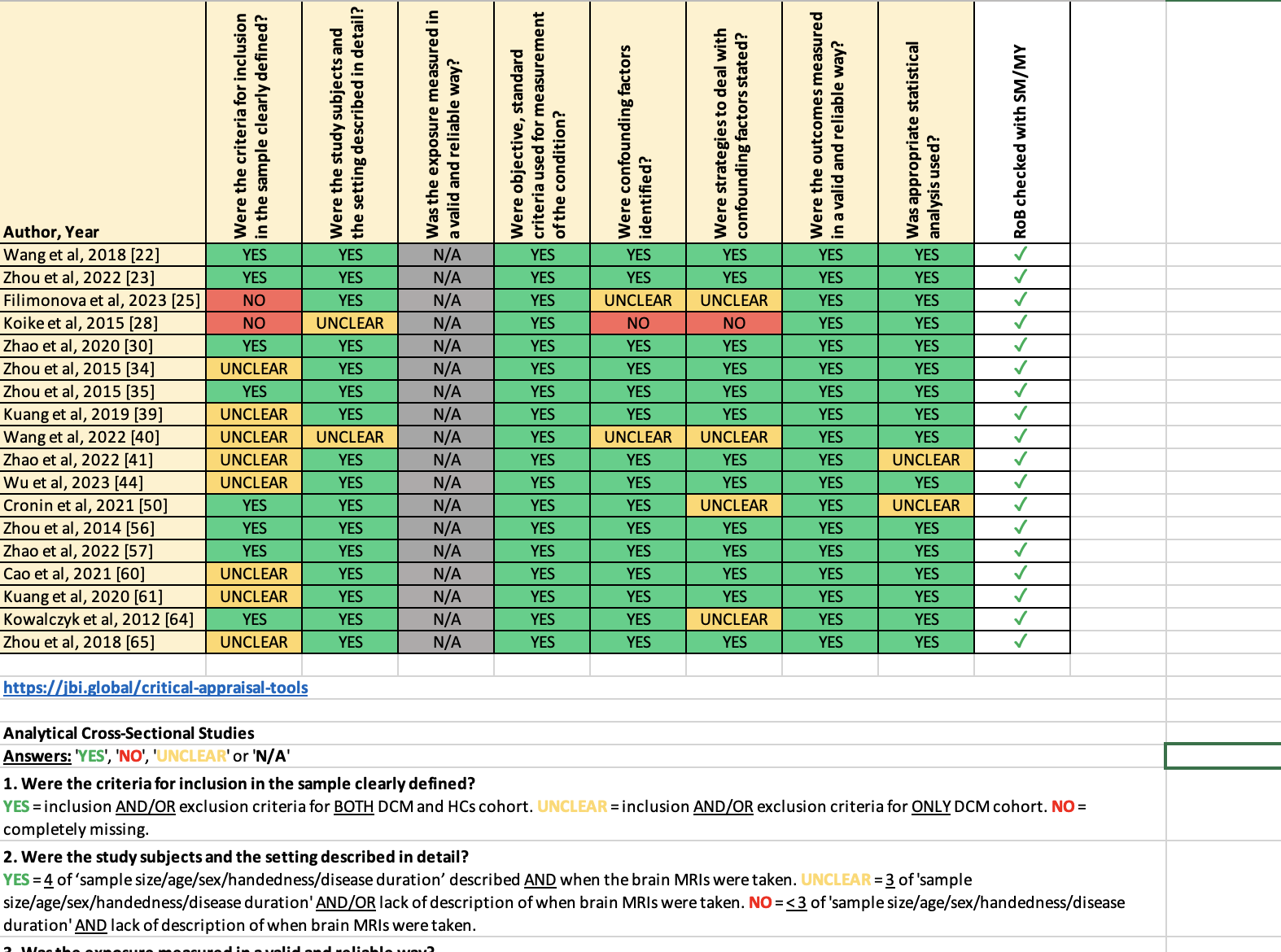


The criteria for inclusion was deemed unclear in eight studies [34, 39, 40, 41, 44, 60, 61, 65] and inappropriate in two studies [25, 28], mainly due to a lack of clearly outlined criteria for the comparator used. The description of study subjects and setting was mostly adequate, except in two studies, which reported on too few subject characteristics (e.g. age, sex, disease duration) [28, 40]. Reassuringly, objective, standard criteria to measure DCM was successfully employed in all cross-sectional studies. Although identification of confounders (e.g. age, other neurological disorders) was mostly adequate, two studies were deemed unclear [25, 40] and one study was identified to have major omissions [28]. Strategies to deal with confounders was performed to a lower quality, with four studies deemed unclear [25, 40, 50, 64] and one study deemed inadequate, most commonly due to lack of a multivariate regression analysis [28]. All cross-sectional studies were deemed to measure outcomes in a valid and reliable way. All studies applied appropriate statistical analysis except two, which were deemed unclear for not stating the specific analysis tool used in their methodology [41, 50].

**Cohort studies**


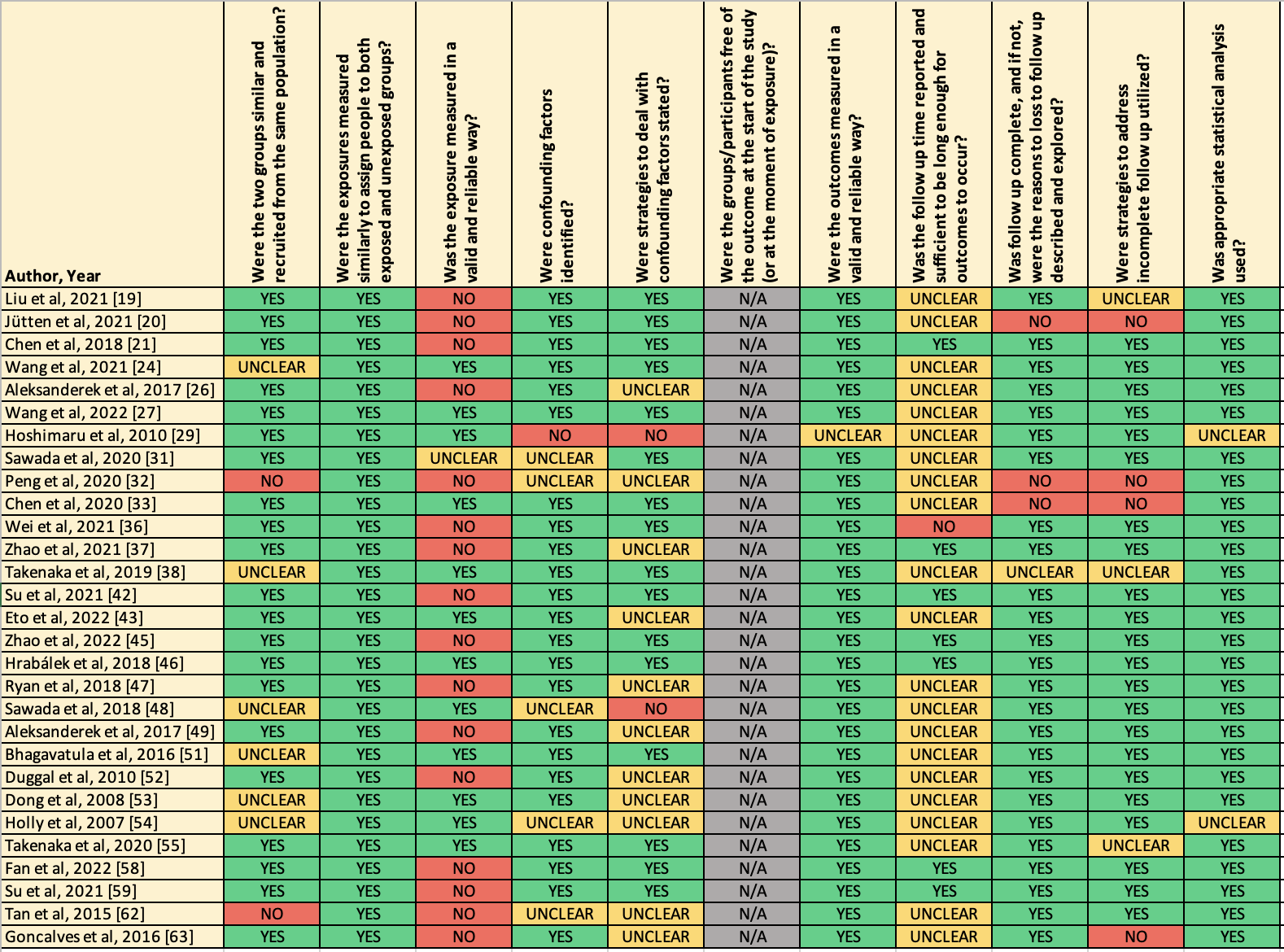


Cohort selection was deemed adequate in most studies, except six that were deemed unclear [24, 48, 51, 53, 54] and two that were deemed inadequate [32, 62], primarily due to lack of a clear study selection criteria. All cohort studies provided an adequate description of how a diagnosis of DCM was made. In contrast, description of the exposure was generally poorly described, with most studies omitting the type of decompression surgery (e.g. laminectomy) performed [19-21, 26, 31, 32, 36, 37, 42, 45, 47, 49, 52, 58, 59, 62, 63]. Identification of confounders was mostly adequate, except in five studies that were deemed unclear [31, 32, 48, 54, 62] and one study that was deemed inadequate [29]. Similar to the cross-sectional studies, strategies to deal with confounders was performed to a lower quality, with just under half of the studies deemed unclear or inadequate [26, 29, 32, 37, 43, 47-49, 52-54, 62, 63]. All except one paper measured outcomes in a valid and reliable way, where the study that was deemed unclear failed to clearly outline their protocol for MRI data acquisition and analysis [29]. Only seven studies were deemed to have a sufficiently long follow up [21, 37, 42, 45, 46, 58, 59], which was deemed to be at least 1.5 months after surgery for clinical measures (e.g. JOA) or one year after surgery for observing changes in brain MRI based on the supporting literature. Future studies would therefore likely benefit from a longer follow-up period as neuroplasticity has been shown to occur over the duration of years (as opposed to days or months). Follow-up was either complete or adequately explored if not in most studies, except one that was deemed unclear [38] and three that were deemed inadequate [20, 32, 33]. Where follow-up was not complete, strategies to deal with incomplete follow-up were not described [19, 20, 32, 33, 38, 55, 63]. Most studies applied appropriate statistical analysis, except two that did not specify the specific analysis tool used [29, 54].

Overall (across both cross-sectional and cohort studies), 42 studies were deemed low risk, whilst 5 studies were deemed to have a moderate risk.

**Supplementary Data 5 – Pre-Surgical Structural Changes**

**Supplementary Data 5. Summary of included studies analysing structural changes in patients with degenerative cervical myelopathy (DCM) before surgical intervention**. Missing information is reported as NR (not reported). Incorrectly reported information is reported as IR. Arrows indicate significantly increased (↑) or decreased (↓) changes.

*ADC = apparent diffusion coefficient; ALFF = amplitude of low-frequency fluctuations; ALS = amyotrophic lateral sclerosis; ASL = arterial spin labelling; CBF = cerebral blood flow; dALFF = dynamic ALFF; DCM = degenerative cervical myelopathy; DWI = diffusion-weighted imaging; EC = effective connectivity; FA = fractional anisotropy; FC = functional connectivity; FCS = FC strength; FLAIR = fluid attenuated inversion recovery; GFA = global FA; gFCD = global FC density; GMV = grey matter volume; HCs = healthy controls; MR Spec = MR spectroscopy; NQA = normalized quantitative anisotropy; ReHo = regional homogeneity; sALFF = static ALFF; SC = structural connectivity; VOA = volume of activation; WMV = white matter volume.*

| **Reference** | **Sample Size (% of M)** | **Mean Age (± SD)** | **Imaging Modality and Timing** | **Outcomes Assessed** | **Structural Changes at Baseline** | |
| --- | --- | --- | --- | --- | --- | --- |
| Liu et al, 2021 [19] | DCM: 45 (37.8), 41 after surgery  HCs: 45 (37.8) | DCM: 51.24 ± 7  HCs: 50.47 ± 5 | T1 at: baseline and 6 months after surgery | GMV | **DCM vs HCs**  ↓ GMV in L caudate nucleus and R thalamus |  |
| Jütten et al, 2021 [20] | DCM: 43 (62.8), 21 after surgery  HCs: 20 (55.0) | DCM: 61 ± 11  HCs: 64 ± 6 | T1 at: baseline and 3 months after surgery | GMV, WMV | **DCM vs HCs**  ↓ GMV in primary sensory cortex (area 1, 2 and 3b), primary motor cortex (area 4a), cerebellum (area IV, V, VIIIa, VIIIb)  No significant change in GMV in fusiform gyrus or hippocampus |  |
| Chen et al, 2018 [21] | DCM: 27 (55.6)  HCs: 11 (54.5) | DCM: 57.9 ± 9.1  HCs: 54.8 ± 8.4 | T1 and rs-fMRI at: baseline only | GMV, WMV, ALFF, ReHo, FC | **DCM vs HCs**  No significant change in GMV or WMV |  |
| Wang et al, 2018 [22] | DCM: 30 (63.3)  HCs: 25 (44.0) | DCM: 51.32 ± 7.52  HCs: 51.2 ± 7.71 | T1 at: baseline only | GMV | **DCM vs HCs**  ↓ GMV in primary sensory cortex (L BA1, L BA2, L and R BA3a, L BA3b), primary motor cortex (L BA4a, R BA4p), somatosensory association cortex (L BA5), SMA (L BA6)  No significant change in GMV in R BA1, R BA2, R BA3b, R BA4a, L BA4p, R BA5, and R BA6 |  |
| Zhou et al, 2022 [23] | DCM: 35 (51.4)  HCs: 35 (51.4) | DCM: 54.27 ± 6.25  HCs: 53.92 ± 7.28 | T1 and rs-fMRI at: baseline only | GMV, gFCD | **DCM vs HCs**  ↓ GMV in L thalamus, B SMA, cerebellum vermis  ↑ GMV in L inferior occipital cortices |  |
| Wang et al, 2021 [24] | DCM: 19 (73.7)  HCs: 16 (NR) | DCM: 55.2 ± 9.4  HCs: 29.4 ± 11.3 | T1 and rs-fMRI at: baseline and 3 months after surgery | Cortical thickness + volume, FC | **DCM vs HCs**  No significant change in cortical thickness or volume |  |
| Filimonova et al, 2023 [25] | DCM: 47 (60.0)  Cervical radiculopathy: 25 (52.0) | DCM: 56.8 ± 8.8  Cervical radiculopathy: 46.4 ± 8.1 | T1 at: baseline only | Volume | **DCM vs Cervical Radiculopathy**  ↓ volume in whole brainstem, medulla, pons, midbrain  ↓ volume in L putamen (adjusted p = 0.087), R putamen/pallidum (p < 0.05 before FDR, adjusted p > 0.1)  No significant change in thalamus or caudate volumes |  |
| Aleksanderek et al, 2017 [26] | DCM: 28 (75.0)  HCs: 10 (50.0) | DCM: 51 ± 2  HCs: 48 ± 4 | MR spectroscopy and DWI at: baseline and 6 months after surgery | FA and mean diffusivity of white matter, metabolite concentrations | **DCM vs HCs**  No significant change in FA or mean diffusivity |  |
| Wang et al, 2022 [27] | DCM: 22 (81.8) | DCM: 56.9 ± 9.0 | DWI at: baseline and 3 months after surgery | SC, FA, GFA, NQA | — |  |
| Koike et al, 2015 [28] | DCM: 16 (NR)  ALS w/o DCM: 19 (57.9)  HCs: 11 (NR) | DCM: 64.5 ± 13.2  ALS w/o DCM: 68.0 ± 8.5  HCs: 62.5 ± 15.3 | DWI at: baseline only | ADC | **DCM vs HCs**  No significant change in ADC in intracranial corticospinal tract (precentral gyrus, corona radiata, posterior limb of internal capsule, cerebral peduncle, genu of corpus collosum) |  |
| Hoshimaru et al, 2010 [29] | DCM: 10 (30.0) | DCM: 65.2 (NR) | FLAIR at: baseline and 3 months after surgery | NR | Mild brain atrophy  No organic brain lesions |  |

In total, 11 studies examined structural changes in 322 DCM patients before surgical intervention. Seven studies assessed GMV/WMV changes [19-25]. Patients with DCM were found to have a smaller GMV across regions of the SMN [20, 22, 23], particularly the primary motor and sensory cortices, and subcortical structures, including the thalamus [19, 23], caudate nucleus [19] and cerebellum [20, 23]. One study reported an increase in GMV in the inferior occipital cortices [23]. Four studies reported no significant change in GMV/WMV, either across the entire brain or within regions of interest [20-22, 24, 25]. This includes Wang *et al* (2018), which reported no significant change in regions of the SMN, despite reporting a smaller GMV for the equivalent regions of the SMN in the contralateral hemisphere [22]. Of the two studies that assessed FA [26, 27], one found no significant change in FA or mean diffusivity between DCM patients and HCs [26]. Koike *et al* (2015) found no significant change in ADC along the intracranial corticospinal tract of DCM patients [28]. Finally, one study observed global atrophy but no lesions in DCM patients [29].

**Supplementary Data 6 – Pre-Surgical Functional Changes**

**Supplementary Data 6. Summary of included studies analysing functional changes in patients with degenerative cervical myelopathy (DCM) before surgical intervention.** Missing information is reported as NR. Incorrectly reported information is reported as IR. Arrows indicate significantly increased (↑) or decreased (↓) changes.

*ADC = apparent diffusion coefficient; ALFF = amplitude of low-frequency fluctuations; ALS = amyotrophic lateral sclerosis; ASL = arterial spin labelling; CBF = cerebral blood flow; dALFF = dynamic ALFF; DCM = degenerative cervical myelopathy; DWI = diffusion-weighted imaging; EC = effective connectivity; FA = fractional anisotropy; FC = functional connectivity; FCS = FC strength; FLAIR = fluid attenuated inversion recovery; GFA = global FA; gFCD = global FC density; GMV = grey matter volume; HCs = healthy controls; MR Spec = MR spectroscopy; NQA = normalized quantitative anisotropy; ReHo = regional homogeneity; sALFF = static ALFF; SC = structural connectivity; VOA = volume of activation; WMV = white matter volume.*

| **Reference** | **Sample Size (% of M)** | **Mean Age (± SD)** | **Imaging Modality and Timing** | **Outcomes Assessed** | **Functional Changes at Baseline** | |
| --- | --- | --- | --- | --- | --- | --- |
| Chen et al, 2018 [21] | DCM: 27 (55.6)  HCs: 11 (54.5) | DCM: 57.9 ± 9.1  HCs: 54.8 ± 8.4 | T1 and rs-fMRI at: baseline only | GMV + WMV, FC, ALFF, ReHo | **DCM vs HCs**  ↑ FC between BA17 and posterior cingulate lobe (BA29/30)  ↓ ALFF in occipital lobe (B precuneus, L calcarine – BA18/7)  ↑ ALFF in cerebellar posterior lobe  ↓ ReHo in occipital lobe (B cuneus, B calcarine, B precuneus – BA19/18/17)  ↑ ReHo in B cerebellar posterior lobe |  |
| Zhou et al, 2022 [23] | DCM: 35 (51.4)  HCs: 35 (51.4) | DCM: 54.27 ± 6.25  HCs: 53.92 ± 7.28 | T1 and rs-fMRI at: baseline only | GMV, gFCD | **DCM vs HCs**  No significant change in distribution of gFCD hubs  ↑ gFCD in B inferior occipital cortices, cerebellum vermis  ↓ gFCD in B SMA, L thalamus, L superior frontal gyrus |  |
| Wang et al, 2021 [24] | DCM: 19 (73.7)  HCs: 16 (NR) | DCM: 55.2 ± 9.4  HCs: 29.4 ± 11.3 | T1 and rs-fMRI at: baseline and 3 months after surgery | Cortical thickness + volume, FC | **DCM vs HCs**  ↓ FC between cerebral cortex and thalamus, hippocampus and putamen (specifically between SMA, pre- and post-central gyrus and putamen)  ↑ FC between visual network and L SMA, sensorimotor system and B insular cortices, between cuneus to sensorimotor system, between cuneus and L superior parietal lobule |  |
| Zhao et al, 2020 [30] | Dataset 1:  DCM: 27 (55.6)  HCs: 11 (54.5)  Dataset 2:  DCM: 26 (53.8)  HCs: 36 (52.8) | Dataset 1:  DCM: 57.9 ± 9.1  HCs: 54.8 ± 8.4  Dataset2:  DCM: 54.7 ± 8.8  HCs: 53.7 ± 8.3 | rs-fMRI at: baseline only | FC, graph theory, BOLD signal | **DCM vs HCs**  ↑ FC between L posterior cerebellum lobe and L superior frontal gyrus and L mid-posterior cerebellum, between L inferior temporal gyrus and L posterior cerebellum lobe and R fusiform gyrus, between L inferior temporal gyrus and B calcarine gyrus and R inferior occipital gyrus, between L superior temporal gyrus and L middle frontal gyrus, between L precentral gyrus and R calcarine gyrus, between L inferior parietal gyrus and B calcarine gyrus and R superior occipital gyrus (*voxel wise analysis*)  ↑ FC between superior frontal gyrus and anterior cingulate gyrus (*region wise analysis*)  ↓ global shortest path, ↑ Eglob, ↓ assortativity in DMN  No significant change in nodal parameters  ↓ BOLD-signal variability in L precentral gyrus, L superior frontal gyrus, L inferior frontal gyrus, L inferior parietal gyrus, L thalamus, L putamen, L pallidum, L angular gyrus, L supramarginal gyrus, L superior temporal gyrus, L inferior temporal gyrus, R cerebellum posterior lobe, R putamen, R pallidum, R precuneus, R cuneus, R calcarine, B posterior cingulate gyrus, B SMA, B middle cingulate gyrus, midbrain |  |
| Sawada et al, 2020 [31] | DCM good recovery: 12 (41.7)  DCM poor recovery: 15 (73.3) | DCM good recovery: 62.7 ± 16.0  DCM poor recovery: 65.5 ± 13.8 | fMRI at: baseline and 7 days after surgery | FC | **Poor (PR) vs Good (GR) Recovery:**  ↑ FC between R middle frontal gyrus with L postcentral gyrus, between L middle frontal gyrus, insular cortex, thalamus and superior temporal gyrus with L postcentral gyrus from R postcentral gyrus, between R superior frontal gyrus and L anterior cingulate cortex, between B postcentral gyrus and contralateral middle frontal gyrus  No significant change in FC in insular cortex, thalamus, prefrontal cortex, middle frontal gyrus and superior frontal gyrus  FC between L postcentral gyrus and R middle frontal gyrus most effective in distinguishing PR from GR |  |
| Peng et al, 2020 [32] | DCM: 43 (62.8), 21 after surgery  HCs: 43 (62.8) | DCM: 49.07 ± 6.73  HCs were age-matched | rs-fMRI at: baseline and 3 months after surgery | FC | **DCM vs HCs**  ↑ FC between L thalamus with B lingual gyrus/cuneus (visual association cortex/primary visual cortex)/R cerebellum posterior lobe |  |
| Chen et al, 2020 [33] | DCM: 30 (53.3), 14 (64.3) after surgery  HCs: 20 (50.0) | DCM: 58.3 ± 8.8, 58.6 ± 10.2 after surgery  HCs: 58.7 ± 8.3 | rs-fMRI at: baseline and 3 months after surgery | FC | **DCM vs HCs**  ↑ FC between L BA7 and R lingual, R vermis, R inferior occipital gyrus, B calcarine, B fusiform gyrus and B precuneus, between R BA7 and R cerebellum (superior lobe), between L BA19 and L cerebellum (inferior lobe), between R BA19 and B cerebellum (inferior lobe) |  |
| Zhou et al, 2015 [34] | DCM: 31 (71.0)  HCs: 31 (71.0) | DCM: 51.38 ± 6.33  HCs: 50.91 ± 6.31 | rs-fMRI at: baseline only | FC, FCS | **DCM vs HCs**  ↓ FCS in L premotor ventral/precentral operculum, R operculum parietale 4  ↑ FCS in L and R inferior parietal lobule, L and R superior parietal lobule, L and R premotor dorsal, R primary somatosensory complex  rsFC network patterns of all seed regions showed group differences between DCM and HCs  ↓ rsFCs in seed regions (L premotor ventral/precentral operculum, R operculum parietale 4) from areas with decreased FCS  ↑ rsFCs in seed regions (L or R inferior parietal lobule, L or R premotor dorsal, L or R superior parietal lobule, R primary somatosensory complex) from areas with increased FCS^i^, except for ↓ rsFC between R superior occipital gyrus and R superior parietal lobule |  |
| Zhou et al, 2015 [35] | DCM: 17 (52.9)  HCs: 17 (47.1) | DCM: 50.53 ± 7.27  HCs: 50.26 ± 7.31 | rs-fMRI at: baseline only | FC | **DCM vs HCs**  *Slow-5 band:*  ↓ rsFC between thalamus and B primary motor cortex, B primary and secondary somatosensory cortex, B premotor cortex (inferior frontal gyrus, superior frontal gyrus/SMA) and R temporal cortex (middle temporal pole, parahippocampagyrus)  ↑ rsFC between thalamus and R prefrontal cortex  No significant change in rsFC between thalamic segments and occipital or posterior parietal cortices  *Slow-4 band*  ↑ rsFC between thalamus and R primary motor cortex, B primary and secondary somatosensory cortex, B prefrontal cortex, R premotor cortex and L temporal cortex  No significant change in rsFC between thalamic segments and occipital, or posterior parietal cortices |  |
| Wei et al, 2021 [36] | DCM: 27 (IR)^j^  HCs: 24 (50.0) | DCM: 53.7 ± 8.1  HCs: 54.2 ± 7.3 | rs-fMRI and ASL at: baseline only | FCS, CBF | **DCM vs HCs**  *Whole grey matter*:  ↓ CBF-FCS coupling in whole grey matter at subject- and group-level analysis  *Region-wise*:  ↓ CBF-FCS coupling in superior frontal gyrus  ↑ CBF-FCS in middle frontal gyrus  ↓ FCS in L precentral gyrus, L postcentral gyrus  ↑ FCS in R hippocampus, R parahippocampus, R amygdala, R pallidum, R thalamus, L thalamus  No significant change in region wise CBF  *Voxel wise*:  ↑ FCS in L thalamus, R thalamus, R anterior cingulate, R hippocampus  ↓ FCS in L precentral gyrus  *ROI wise*:  ↓ CBF-FCS coupling in L and R thalamus, R calcarine gyrus  ↓ FCS in the L precentral gyrus, L postcentral gyrus, R precentral gyrus  ↑ FCS in L and R thalamus |  |
| Zhao et al, 2021 [37] | DCM: 27 (55.6)  HCs: 11 (54.5) | DCM: 57.9 ± 9.1  HCs: 54.8 ± 8.4 | rs-fMRI at: baseline only | EC | **DCM vs HCs**  ↑ strength of bidirectional connections between cerebellum and L secondary visual cortex, unidirectional connection from R secondary visual cortex to cerebellum |  |
| Takenaka et al, 2019 [38] | DCM: 28 (50.0), 26 after surgery  HCs: 28 (50.0) | DCM: 66.5 ± 10.9  HCs: 66.5 ± 11.0 | rs-fMRI at: baseline and 6 months after surgery | FC | **DCM vs HCs**  ↓ FC in 25 clusters, including between R superior frontal gyrus and primary visual cortex, L intracalcarine cortex and L lingual gyrus  ↑ FC in 5 clusters |  |
|  |  |  |  |  |  |  |
| Kuang et al, 2019 [39] | DCM: 33 (48.5)  HCs: 33 (45.5) | DCM: 54.78 ± 8.41  HCs: 53.52 ± 8.13 | rs-fMRI at: baseline only | FC, ALFF, ReHo | **DCM vs HCs**  ↑ FC between L medial superior frontal gyrus and L postcentral gyrus and L rolandic operculum, between L supramarginal gyrus and L calcarine and L postcentral gyrus  ↓ FC between L medial superior frontal gyrus and R medial superior frontal gyrus  ↑ zALFF in L medial superior frontal gyrus  ↑ zReHo in L supramarginal gyrus |  |
| Wang et al, 2022 [40] | Patient cohort: 99 (61.6)  Asymptomatic SCC: 21 (61.9)  Mild DCM: 48 (60.4)  Moderate/Severe DCM: 30 (63.3)  HCs: 17 (52.9) | Patient cohort: 59.0 ± 10.7  Asymptomatic SCC: 57.0 ± 14.2  Mild DCM: 60.3 ± 10.3  Moderate/Severe DCM: 58.4 ± 8.6  HCs: 41.0 ± 14.0 | rs-fMRI at baseline only | FC, graph theory | *HCs:* 23 regions as brain hubs. Transitioning to asymptomatic SCC, 89 connections between cerebellum, brainstem and thalamus ↑, while 58 connections between frontal lobe, thalamus and supplementary regions ↓. Driven by changes in R superior frontal gyrus, L precentral gyrus, L putamen, L SMA, B thalamus, regions responsible for visual input and spatial navigation, and several subregions of cerebellum and brainstem.  *Asymptomatic SCCs:* 20 regions as brain hubs. Transitioning to mild DCM, 85 connections with the cerebellum ↑, while 94 connections between the cerebellum and anterior cingulate, R precentral gyrus, B superior frontal gyrus and B SMA ↓. Driven by changes in centrality of anterior cingulate, R precentral gyrus, B superior frontal gyrus, B SMA and several subregions of cerebellum.  *Mild DCM:* 12 regions as brain hubs. No cortical regions were identified as brain hubs, but several subregions of cerebellum and brainstem maintained roles. Transitioning to moderate/severe DCM, 52 connections between cerebellar vermis and brainstem to L superior frontal gyrus, L precentral gyrus and thalamus ↑, while 64 connections within the cerebellum ↓.  *Moderate/Severe DCM:* 10 regions as brain hubs. |  |
| Zhao et al, 2022 [41] | DCM: 88 (51.1)  HCs: 77 (50.6) | DCM: 49.22 ± 7.91  HCs: 45.13 ± 8.76 | rs-fMRI at: baseline only | FC, graph theory | **DCM vs HCs**  All showed 4 dynamic functional connection states with ↑ MDT in state 2.No significant change in number of transitions between four states.  *State 1*:  ↑ FC between sensorimotor network and language network  ↓ FC between salience network and precuneus  *State 2:*  ↑ FC in executive control network and salience network  ↓ FC in default mode network  *State 3:*  ↑ FC in language network and auditory network  ↓ FC in R default mode network and sensorimotor network  *State 4*:  ↑ FC in executive control network  ↓ FC in visual network and sensorimotor network  ↑ variance in Eloc in R Heschel gyrus, L and R superior temporal gyrus, L middle temporal gyrus  No significant change in variance of Eglob  Significantly different nodal degree in L and R superior frontal gyrus (orbital part), L middle occipital gyrus, L superior frontal gyrus (medial orbital), R precuneus, L gyrus rectus, L angular gyrus  Significantly different nodal efficiency in L superior temporal gyrus, L superior frontal gyrus (orbital part), L and R middle occipital gyrus, L and R lenticular nucleus of pallidum, L and H superior frontal gyrus (medial orbital)  Significantly different betweenness centrality in R middle temporal gyrus, L precuneus  Significantly different nodal cluster coefficient in R superior temporal gyrus, R Heschel gyrus, L temporal pole of superior temporal gyrus |  |
| Su et al, 2021 [42] | Dataset 1:  DCM: 27 (55.6)  HCs: 11 (54.5)  Dataset 2:  DCM: 26 (53.8)  HCs: 36 (52.8) | Dataset 1:  DCM: 57.9 ± 9.1  HCs: 54.8 ± 8.4  Dataset 2:  DCM: 54.7 ± 8.8  HCs: 53.7 ± 8.3 | rs-fMRI at: baseline only | FC | **DCM vs HCs**  ↑ FC mainly between frontal lobe and cerebellum and thalamus, between temporal lobe and cerebellum and thalamus |  |
| Eto et al, 2022 [43] | DCM: 15 (80.0) | DCM: 65.3 ± 11.3 | rs-fMRI at: baseline and 6 months after surgery | FC | — |  |
| Wu et al, 2023 [44] | Total DCM: 32 (50.0)  DCM w/ normal gait pattern: 16 (50.0)  DCM w/ abnormal gait pattern: 16 (50.0)  HCs: 16 (50.0) | Total DCM: 52.47  DCM w/ normal gait pattern: 51.38 ± 6.91  DCM w/ abnormal gait pattern: 53.56 ± 9.51  HCs: 52.63 ± 9.51 | rs-fMRI at: baseline only | FC, ALFF | **Abnormal Gait Pattern DCM vs HCs**  ↑ zALFF in R caudate  ↓ zALFF in R postcentral gyrus, R paracental lobule  ↑ FC between caudate and L angular gyrus, L and R precuneus, L and R posterior cingulate cortex  **Normal Gait Pattern DCM vs HCs**  ↑ zALFF in R caudate  ↓ zALFF in R postcentral gyrus, R paracental lobule  **Abnormal vs Normal Gait Pattern DCM**  ↓ zALFF in R caudate |  |
| Zhao et al, 2022 [45] | Total DCM: 66  DCM w/ depression: 33 (51.1)  DCM  DCM w/o depression: 33 (sex-matched)  HCs: 33 (51.5) | DCM w/ depression: 55.2 ± 7.28  DCM w/o depression: age-matched  HCs: 55.1 ± 6.41 | rs-fMRI at: baseline only | FC | **Depressed vs Non-Depressed DCM:**  ↓ FC between L dorsal caudate and L inferior frontal operculum |  |
| Hrabálek et al, 2018 [46] | DCM: 20 (45.0) | DCM: 56.6 (NR) | fMRI at: baseline, 6 months and 12 months after surgery | Activation during active wrist flexion and extension | **Abnormal vs Normal MEP DCM**  ↑ activation in ipsilateral cerebellum for both R and L wrists |  |
| Ryan et al, 2018 [47] | DCM: 22 (86.4)  HCs: 10 (70.0) | DCM: 50 ± 10.9  HCs: 48 ± 9.9 | fMRI at: baseline, 6 weeks and 6 months after surgery | VOA during finger tapping, BOLD signal | **DCM vs HCs**  ↓ VOA in contralateral primary sensory cortex, ipsilateral SMA  ↓ % BOLD signal in contralateral SMA |  |
| Sawada et al, 2018 [48] | DCM: 6 (50.0)  HCs: 5 (100.0) | DCM: 48 ± 12  HCs: 39 ± 12 | fMRI at: baseline and 7 days after surgery | Activation during finger tapping | **DCM vs HCs**  Both had activation in precentral gyrus and cerebellum, but DCM additionally had activation in SMA |  |
| Aleksanderek, et al 2017 [49] | Total DCM: 28 (75.0)  Mild DCM: 15 (86.7)  Moderate DCM: 13 (61.5)  HCs: 10 (50.0) | Mild DCM: 50.1 ± 12  Moderate DCM: 53.0 ± 9  HCs: 48.0 ± 12 | fMRI and MR spectroscopy at: baseline and 6 months after surgery | VOA during finger tapping, metabolite concentrations | **Mild DCM vs HCs**  No significant change in VOA  **Moderate DCM vs HCs**  No significant change in VOA  **Mild vs Moderate DCM**  ↑ VOA near primary motor cortex (L parietal lobe, postcentral gyrus, BA5) |  |
| Cronin et al, 2021 [50] | DCM: 23 (60.9) | DCM: 65 ± 13 | fMRI at: baseline only | VOA during finger tapping, BOLD signal | — |  |
| Bhagavatula et al, 2016 [51] | DCM: 17 (94.1)  HCs: 12 (NR) | DCM: 51.76 ± 10.67  HCs: NR | fMRI at: baseline and 6 months after surgery | VOA during finger tapping | **DCM vs HCs**  ↑ VOA in L (contralateral) primary sensory cortex, R (ipsilateral) primary sensory cortex, cerebellum, combined sensory and motor cortices |  |
| Duggal et al, 2010 [52] | DCM: 12^q^ (75.0)  HCs: 10 (40.0) | DCM: 49.6 ± 12.8  HCs: 41.0 ± 8.8 | fMRI at: baseline and 6 months after surgery | VOA during finger tapping | **DCM vs HCs**  ↓ VOA in primary sensory cortex  ↑ VOA in premotor cortex, primary motor cortex |  |
| Dong et al, 2008 [53] | DCM: 8 (50.0)  HCs: 6 (NR) | DCM: 61.125  HCs: NR | fMRI at: baseline, 3 months and 6 months after surgery | VOA and activation magnitude during wrist extension and 3-finger pinch | **DCM vs HCs**  ↓ VOA in contralateral primary sensory cortex, primary motor cortex, dorsal premotor area during pinch task, but same VOA in these regions during wrist extension  Between-task comparison showed ↓VOA in ipsilateral primary motor cortex and primary sensory cortex and ↓ activation magnitude in contralateral SMA during 3-finger pinch than during wrist extension |  |
| Holly et al, 2007 [54] | DCM: 4 (25.0)  HCs: 5 (NR) | DCM: 58.25  HCs were age-matched | fMRI at: baseline and at least 2 of 6 weeks, 3 months or 6 months after surgery | Activation during ankle dorsiflexion or wrist extension | **DCM cases vs HCs**  *Case 1*: ↑ activity in primary motor cortex, premotor area and SMA  *Case 2:* ↑ activity in posterior and medial to the primary motor cortex for wrist  *Case 3*: ↑ activity in B medial motor areas for leg  *Case 4*: ↑ activity in dorsal premotor area |  |
| Takenaka et al, 2020 [55] | DCM: 28 (50.0), 26 after surgery  HCs: 28 (50.0) | DCM: 67 ± 11  HCs: 67 ± 11 | rs-fMRI at: baseline and 6 months after surgery | ALFF | **DCM vs HCs**  ↓ ALFF in B posterior supramarginal gyrus  ↑ ALFF in B primary sensorimotor cortices, L visual cortex (occipital pole, lingual gyrus and intracalcrine cortex) |  |
| Zhou et al, 2014 [56] | DCM: 19 (57.9)  HCs: 19 (52.6) | DCM: 49.63 ± 7.36  HCs: 49.46 ± 7.21 | rs-fMRI at: baseline only | ALFF | **DCM vs HCs**  ↑ ALFF in R precentral gyrus, R postcentral gyrus, L SMA |  |
| Zhao et al, 2022 [57] | Dataset 1:  DCM: 21 (52.4)  HCs: 11 (54.5)  Dataset 2:  DCM: 33 (48.5)  HCs: 39 (51.3) | Dataset 1:  DCM: 53.3 ± 9.13  HCs: 54.8 ± 8.4  Dataset 2:  DCM: 53.5 ± 11.9  HCs: 53.7 ± 8.3 | rs-fMRI at: baseline only | ALFF | **DCM vs HCs**  ↑ ALFF in L precentral gyrus, B superior frontal gyrus  ↓ ALFF in R precuneus, R calcarine gyrus  **High JOA vs Low JOA DCM**  ‘Relative-high-JOA’ compared to ‘relative-low-JOA’ DCM had ↓ zALFF in L precentral gyrus  ‘High-JOA-high-MSCC’ compared to ‘low-JOA-low-MSCC’ DCM had ↓ zALFF in L precentral gyrus |  |
| Fan et al, 2022 [58] | DCM: 47 (IR)  HCs: 44 (IR) | DCM: 51.3 ± 2.8  HCs: 51.7 ± 3.6 | rs-fMRI at: baseline only | sALFF, dALFF | **DCM vs HCs**  ↑ sALFF in L thalamus, L putamen  ↓ dALFF in B postcentral gyrus  DCM patients could successfully be identified from HCs using sALFF and dALFF with key voxels in B SMA, B primary motor cortex, B primary sensory cortex, B posterior cerebellum.  Key voxels for classification accuracy in B frontal cortices and B temporal gyri for sALFF and B frontal cortices, B inferior temporal gyrus, B inferior occipital gyrus and L posterior cerebellum for dALFF |  |
| Su et al, 2023 [59] | DCM: 62 (50.0)  HCs: 60 (50.0) | DCM: 53.3 ± 7.38  HCs: 53.4 ± 7.47 | rs-fMRI at: baseline only | ALFF | **DCM vs HCs**  ↑ ALFF in L middle cingulate cortex, L superior frontal gyrus  ↓ ALFF in R precentral gyrus, L primary visual cortex  **Postoperative vs Non-Postoperative Axial Pain DCM**  ↑ ALFF in middle cingulate cortex |  |
| Cao et al, 2021 [60] | DCM: 67 (52.2)  HCs: 60 (53.3) | DCM: 47.52 ± 7.36  HCs: 45.51 ± 10.64 | rs-fMRI at: baseline only | Graph theory | **DCM vs HCs**  ↓ Lp, ↓ Cp, ↓ λ, ↑ γ, ↑ σ, ↑ Eglob  No significant change in Eloc  ↓ nodal centralities, in B lingual gyrus, B posterior cingulate gyrus, B putamen, L postcentral gyrus, R middle temporal gyrus  ↑ nodal centralities in B inferior cerebellum, B precuneus, B angular gyrus, B superior frontal gyrus (medial part), L anterior cingulate and paracingulate gyri, L median cingulate gyrus, R superior frontal gyrus (medial part) |  |
| Kuang et al, 2020 [61] | DCM: 33 (48.5), 31 included in analysis  HCs: 33 (45.5), 31 included in analysis | DCM: 54.78 ± 8.41  HCs: 53.53 ± 8.13 | T1 at: baseline only | Graph theory | **DCM vs HCs**  ↑ Eglob, ↓ Eloc, ↓ Lp, ↓ Cp, ↓σ  ↑ AUC for Eglob, ↓ AUC for Eloc, ↓ AUC for Lp, ↓ AUC for Cp, ↓ AUC for σ  ↑ betweenness in L superior parietal gyrus, L SMA  ↓ betweenness in R middle occipital gyrus  Equal resilience to random failure, but maximum relative size of giant connected components was ~10% larger in HCs on removal of 44 nodes in targeted attack (significant on removal of 44, 45 and 50 nodes) |  |
| Tan et al, 2015 [62] | DCM: 21 (42.9)  HCs: 21 (sex-matched) | DCM: 47.95 ± 7  HCs were age-matched | rs-fMRI at: baseline and 3 months after surgery | ReHo | **DCM vs HCs**  ↓ ReHo in L postcentral gyrus, L precentral gyrus  ↑ ReHo in R superior parietal lobule |  |

A total of 36 studies explored functional changes in 1200 DCM patients before surgical intervention. Of the 19 studies focussing on an aspect of FC, 16 assessed FC [21, 24, 30-35, 38-45], two assessed FC strength (FCS; a marker of voxel-level degree centrality) [34, 36], one assessed global FC density (gFCD; a marker of whole-brain FC patterns at the voxel level) [23] and one assessed effective connectivity (EC; a marker of the directional connection across brain region) [37]. All observed alterations in FC [21, 23, 24, 30-45]. Eight studies reported decreased FC within regions of the SMN [23, 24, 34-36, 40, 41]. Wang *et al* (2022) observed increased FC changes in several areas, including parts of the SMN [40]. Two other studies similarly identified increased FC within the SMN [34, 39]. Altered FC in visual-associated regions were also commonly observed, with eight studies reporting increased FC [21, 23, 24, 32, 33, 37, 39, 40]. Conversely, one study reported predominantly decreased FC within the brain, including three FC between the visual cortex and superior frontal gyrus [38]. Other brain networks were also impacted, with increased FC reported in the DMN [30] and pain-related network [31]. Zhao *et al* (2022) assessed dynamic FC using a sliding window analysis and found that, although DCM patients and HCs shared the same four functional states, multiple brain networks exhibited altered coupling in each state [41]. Zhao *et al* (2015) utilised two slow frequency bands (0.01-0.027 Hz and 0.027-0.073 Hz) to characterise thalamocortical disturbances [34], with another study also identifying changes in subcortical regions including the thalamus and cerebellum [42].

Nine studies examined brain activation/VOA, of which six did so during finger tapping [47-52], one during wrist flexion and extension [46], one during wrist extension and 3-finger pinch [53] and one during wrist flexion and ankle dorsiflexion [54]. Differences in activation between DCM patients and HCs were identified in regions within the SMN and cerebellum [46-49, 51-53]. Despite this, multiple studies seemed to conflict with regards to the directionality of changes. Two studies reported no significant change in activation between DCM patients and HCs [49, 53], although Dong *et al* (2008) did report on differences between varying severities of DCM [53].

All eight of the studies that investigated ALFF identified some alterations in DCM patients [21, 39, 44, 55-59]. Cortical regions that were found to have increased ALFF include the primary motor and/or sensory cortex [55-57], SMA [56], medial superior frontal gyrus [39, 57] and the visual cortex [55]. Cortical regions associated with a decreased ALFF include the posterior supramarginal gyrus [55], precuneus [57], calcarine gyrus [57] and the visual [21, 59] and primary sensory cortices [44, 58]. Subcortical structures were also found to have altered ALFF, with increased ALFF measured in the thalamus [58], putamen [58] and cerebellum [21]. Fan *et al* (2022) illustrated the significance of investigating ALFF changes by using ALFF to differentiate DCM patients from HCs with high accuracy [58].

Five studies used graph theory analysis [30, 40, 41, 60, 61]. Zhao *et al* (2020) found decreased global shortest path and assortativity but increased global efficiency (Eglob) in the DMN of DCM patients [30]. Another paper reported altered graph theory parameters (including increased Eglob), with nodal centralities decreased in the sensorimotor and visual regions and increased in the DMN and cerebellum [60]. Kuang *et al* (2020) found decreased characteristic path length (Lp), clustering coefficient (Cp), small worldness (σ) and local efficiency (Eloc) and increased Eglob, with reported betweenness increased in the SMA and superior parietal gyrus but decreased in the middle occipital gyrus [61]. One study reported increased variance in Eloc, but no significant change in Eglob, with significant differences in betweenness centrality and nodal degree, efficiency, and cluster coefficient [41]. Wang *et al* (2022) observed a decreasing number of brain hubs important for sensorimotor function as pathogenesis progressed [40].

Three papers examined BOLD signal changes before surgery, two of which identified decreased BOLD signal % or variability within the SMA [30, 47], with Zhao *et al* (2020) further identifying decreased BOLD signal variability in the SMN, DMN and visual network [30]. All three papers interested in ReHo identified differences between DCM and HCs, with no overlap in identified regions [21, 39, 62]. Decreased ReHo was identified in the primary motor and sensory cortices [62] and occipital lobe [21], whilst increased ReHo was identified in the supramarginal gyrus [62], superior parietal lobule [62] and cerebellum [21].

**Supplementary Data 7 – Pre-Surgical ‘Other MRI’ Techniques**

**Supplementary Data 7. Summary of included studies utilising ‘other MRI’ techniques in patients with degenerative cervical myelopathy (DCM) before surgical intervention.** Missing information is reported as NR. Incorrectly reported information is reported as IR. Arrows indicate significantly increased (↑) or decreased (↓) changes.

*ADC = apparent diffusion coefficient; ALFF = amplitude of low-frequency fluctuations; ALS = amyotrophic lateral sclerosis; ASL = arterial spin labelling; CBF = cerebral blood flow; dALFF = dynamic ALFF; DCM = degenerative cervical myelopathy; DWI = diffusion-weighted imaging; EC = effective connectivity; FA = fractional anisotropy; FC = functional connectivity; FCS = FC strength; FLAIR = fluid attenuated inversion recovery; GFA = global FA; gFCD = global FC density; GMV = grey matter volume; HCs = healthy controls; MR Spec = MR spectroscopy; NQA = normalized quantitative anisotropy; ReHo = regional homogeneity; sALFF = static ALFF; SC = structural connectivity; VOA = volume of activation; WMV = white matter volume.*

| **Reference** | **Sample Size (% of M)** | **Mean Age (± SD)** | **Imaging Modality and Timing** | **Outcomes Assessed** | **Changes at Baseline** | |
| --- | --- | --- | --- | --- | --- | --- |
| Aleksanderek et al, 2017 [26] | DCM: 28 (75.0)  HCs: 10 (50.0) | DCM: 51 ± 2  HCs: 48 ± 4 | MR spectroscopy at: baseline and 6 months after surgery | Metabolite concentrations | **DCM vs HCs**  No significant change in metabolite concs |  |
| Wei et al, 2021 [36] | DCM: 27 (IR)^j^  HCs: 24 (50.0) | DCM: 53.7 ± 8.1  HCs: 54.2 ± 7.3 | ASL at: baseline only | CBF | **DCM vs HCs**  ↓ CBF in L precentral gyrus, L postcentral gyrus, L and R precuneus, R calcarine gyrus |  |
| Aleksanderek, et al 2017 [49] | Total DCM: 28 (75.0)  Mild DCM: 15 (86.7)  Moderate DCM: 13 (61.5)  HCs: 10 (50.0) | Mild DCM: 50.1 ± 12  Moderate DCM: 53.0 ± 9  HCs: 48.0 ± 12 | fMRI and MR spectroscopy at: baseline and 6 months after surgery | Metabolite concentrations | **Mild DCM vs HCs**  ↓ NAA/Cr ratio in primary motor cortex  **Moderate DCM vs HCs**  No significant change in NAA/Cr  **Mild vs Moderate DCM**  ↓ NAA/Cr ratio in primary motor cortex |  |
| Goncalves et al, 2016 [63] | DCM: 17 (94.1)  HCs: 8 (62.5) | DCM: 52 ± 2  HCs: 48 ± 3 | MR spectroscopy at: baseline, 6 weeks and 6 months after surgery | Metabolite concentrations | — |  |
| Kowalczyk et al, 2012 [64] | DCM: 24 (66.7)  HCs: 11 (63.6) | DCM: 53 ± 2  HCs: 46 ± 4 | MR spectroscopy at: baseline only | Metabolite concentration | **DCM vs HCs**  ↓ NAA/Cr ratios in motor cortex. Tukey’s post hoc analysis showed no significant change when comparing left side but ↓NAA/Cr when comparing right side motor cortex  No significant change in mI/Cr ratio or other metabolite concentrations |  |
| Zhou et al, 2018 [65] | DCM: 18 (72.2)  HCs: 18 (72.2) | DCM: 51.94 ± 7.81  HCs: 50.50 ± 7.12 | pCASL at: baseline only | CBF | **DCM vs HCs**  ↓ CBF in L premotor ventral/precentral operculum, B dorsal anterior cingulate cortex  ↑ CBF in L paracentral lobule, R paracentral lobule/SMA, R postcentral gyrus  L paracentral lobule, R paracentral lobule/SMA and R postcentral gyrus could differentiate myelopathy-related impairment of DCM from HCs |  |

Six studies utilised other MRI techniques (MR spectroscopy and ASL) in 142 DCM patients before surgical intervention. Four studies analysed metabolite concentrations in DCM patients before surgery [26, 49, 63, 64]. Whilst one study found no significant changes in metabolite concentration [26], two observed decreased N-acetylaspartate/creatine (NAA/Cr) ratios in the primary motor cortex [49, 64]. Despite Aleksanderek *et* al (2017) reporting changes in metabolites between mild DCM patients and HCs, they did not identify such a change in the primary motor cortex of moderate DCM patients [49]. Of the two studies that assessed cerebral blood flow (CBF), one study identified decreased CBF mainly in the sensorimotor regions [36], whilst the other identified increased CBF in sensory and motor-sensory processing regions [65]. Zhou *et al* (2018) also identified decreased CBF in the premotor ventral and precentral operculum regions [65].

**Supplementary Data 8 – Post-Surgical Structural Changes**

**Supplementary Data 8. Summary of included studies analysing structural changes in patients with degenerative cervical myelopathy (DCM) after surgical intervention.** Missing information is reported as NR. Incorrectly reported information is reported as IR. Arrows indicate significantly increased (↑) or decreased (↓) changes.

*ADC = apparent diffusion coefficient; ALFF = amplitude of low-frequency fluctuations; ALS = amyotrophic lateral sclerosis; ASL = arterial spin labelling; CBF = cerebral blood flow; dALFF = dynamic ALFF; DCM = degenerative cervical myelopathy; DWI = diffusion-weighted imaging; EC = effective connectivity; FA = fractional anisotropy; FC = functional connectivity; FCS = FC strength; FLAIR = fluid attenuated inversion recovery; GFA = global FA; gFCD = global FC density; GMV = grey matter volume; HCs = healthy controls; MR Spec = MR spectroscopy; NQA = normalized quantitative anisotropy; ReHo = regional homogeneity; sALFF = static ALFF; SC = structural connectivity; VOA = volume of activation; WMV = white matter volume.*

| **Reference** | **Type of Decompression Surgery** | **Imaging Modality and Timing** | **Outcomes Assessed** | **Structural Changes After Surgery** | |
| --- | --- | --- | --- | --- | --- |
| Liu et al, 2021 [19] | NR | T1 at: baseline and 6 months after surgery | GMV | **Post- vs Pre-Surgery DCM**  ↓ GMV in B cerebellar posterior lobe  ↑ GMV in brainstem  **Post-Surgery DCM vs HCs**  ↓ GMV in L caudate nucleus  ↑ GMV in R inferior temporal gyrus, R middle orbitofrontal cortex, B lingual gyrus/precuneus/posterior cingulate cortex |  |
| Jütten et al, 2021 [20] | NR | T1 at: baseline and 3 months after surgery | GMV, WMV | **Post- vs Pre-Surgery DCM**  No significant change in GMV or WMV in primary motor cortex, primary sensory cortex, cerebellum, supplementary motor area |  |
| Wang et al, 2021 [24] | 8/19 cervical laminectomy and fusion  7/19 laminoplasty  3/19 anterior cervical discectomy and fusion  1/19 anterior cervical corpectomy | T1 and rs-fMRI at: baseline and 3 months after surgery | Cortical thickness + volume, FC | **Post- vs Pre-Surgery DCM**  No significant change in cortical thickness or volume2 |  |
| Aleksanderek et al, 2017 [26] | 19/28 1-level decompression  9/28 2-level decompression | MR spectroscopy and DWI at: baseline and 6 months after surgery | FA and mean diffusivity of white matter, metabolite concentrations | **Post-Surgery DCM vs HCs**  No significant change in FA and mean diffusivity |  |
| Wang et al, 2022 [27] | 10/22 cervical laminectomy and fusion  9/22 laminoplasty  2/22 anterior cervical discectomy and fusion  1/22 anterior cervical corpectomy | DWI at: baseline and 3 months after surgery | SC, FA, GFA, NQA | **Post- vs Pre-Surgery DCM**  No significant change in GFA  ↓ NQA particularly in prefrontal and frontal areas  ↑ NQA in B internal capsule, B external capsule, corpus collosum (body and genu), B anterior and superior corona radiata, L posterior corona radiata, B corticospinal tract, B cingulum, L superior fronto-occipital fasciculus, B superior longitudinal fasciculus  ↓ SC in frontal lobes, between frontal lobes and R caudate, R insula, L precuneus, primary sensorimotor regions and cingulate gyrus |  |
| Hoshimaru et al, 2010 [29] | 10/10 cervical laminoplasty | FLAIR at: baseline and 3 months after surgery | NR | Mild brain atrophy  No organic brain lesions |  |

Six studies examined structural changes in 141 DCM patients after surgical intervention. Two studies found no significant change in GMV when comparing 3 months post-surgical DCM patients against pre-surgical patients [20, 24]. Liu *et al* (2021), however, found that GMV decreased in the cerebellum and increased in the brainstem after a longer duration follow-up after surgery of 6 months [19]. When comparing against HCs, they found that GMV in post-surgical patients decreased in the caudate nucleus and increased in the inferior temporal gyrus, middle orbitofrontal cortex, and lingual/precuneus/posterior cingulate cortex. Two papers assessed FA after surgery [26, 27]. Aleksanderek *et al* (2017) found no significant change in FA or mean diffusivity in the primary motor and sensory cortices and the cerebellum [26]. Whilst Wang *et al* (2022) found no difference in global fractional anisotropy (GFA) between post- and pre-surgical DCM patients, they identified decreased structural connectivity within and between the prefrontal/frontal lobes [27]. Hoshimaru *et al* (2010) also reported mild atrophy and no lesions after surgery [29].

**Supplementary Data 9 – Post-Surgical Functional Changes**

**Supplementary Data 9. Summary of included studies analysing functional changes in patients with degenerative cervical myelopathy (DCM) after surgical intervention.** Missing information is reported as NR. Incorrectly reported information is reported as IR. Arrows indicate significantly increased (↑) or decreased (↓) changes.

*ADC = apparent diffusion coefficient; ALFF = amplitude of low-frequency fluctuations; ALS = amyotrophic lateral sclerosis; ASL = arterial spin labelling; CBF = cerebral blood flow; dALFF = dynamic ALFF; DCM = degenerative cervical myelopathy; DWI = diffusion-weighted imaging; EC = effective connectivity; FA = fractional anisotropy; FC = functional connectivity; FCS = FC strength; FLAIR = fluid attenuated inversion recovery; GFA = global FA; gFCD = global FC density; GMV = grey matter volume; HCs = healthy controls; MR Spec = MR spectroscopy; NQA = normalized quantitative anisotropy; ReHo = regional homogeneity; sALFF = static ALFF; SC = structural connectivity; VOA = volume of activation; WMV = white matter volume.*

| **Reference** | **Type of Decompression Surgery** | **Imaging Modality and Timing** | **Outcomes Assessed** | **Functional Changes After Surgery** | |
| --- | --- | --- | --- | --- | --- |
| Wang et al, 2021 [24] | 8/19 cervical laminectomy and fusion  7/19 laminoplasty  3/19 anterior cervical discectomy and fusion  1/19 anterior cervical corpectomy | T1 and rs-fMRI at: baseline and 3 months after surgery | Cortical thickness + volume, FC | **Post- vs Pre-Surgery DCM**  ↑ FC between R superior frontal gyrus and L cerebellum area 6, between L superior frontal gyrus and L cerebellum area 10  ↓ FC between L thalamus and L cerebellum area 10, between R superior frontal gyrus and L postcentral gyrus |  |
| Sawada et al, 2020 [31] | Anterior fusion, or laminoplasty if more than 3 levels of spinal cord compression  6/12 of good recovery group underwent posterior decompression  10/15 of poor recovery group underwent posterior decompression | fMRI at: baseline and 7 days after surgery | FC | **Post-Surgery Poor (PR) vs Good (GR) Recovery:**  No significant change in FC (*seed-to-voxel*)  PR group had significant FC between postcentral gyrus and dorsolateral prefrontal cortex, whilst GR group had no significant FC (*seed-to-seed*)  **Post- vs Pre-Surgery Poor Recovery**  ↓ FC between postcentral gyrus a dorsolateral prefrontal cortex (including superior and middle frontal gyrus) |  |
| Peng et al, 2020 [32] | NR | rs-fMRI at: baseline and 3 months after surgery | FC | **Post- vs Pre-Surgery DCM**  ↓ FC between B thalamus and paracentral lobe/precentral gyrus  ↑ FC between B thalamus with posterior cingulate lobe, angular gyrus and medial prefrontal  **Post-Surgery DCM vs HCs**  ↓ FC between R thalamus with B paracentral lobe/precentral gyrus  ↑ FC between R thalamus with pons/superior temporal gyrus |  |
| Chen et al, 2020 [33] | 18/30 anterior decompression  12/30 posterior decompression | rs-fMRI at: baseline and 3 months after surgery | FC | **Post- vs Pre-Surgery DCM**  ↓ FC in L and R BA7, L BA19  No significant change in FC in R BA19 |  |
| Takenaka et al, 2019 [38] | 25/28 cervical laminoplasty  3/28 anterior decompression and fusion | rs-fMRI at: baseline and 6 months after surgery | FC | **Post- vs Pre-Surgery DCM**  ↑ FC in 13 clusters. FC between L supracalcarine cortex and R superior frontal gyrus overlapped with clusters showing ↑ FC in pre-surgery DCM. No cluster showed ↓ FC |  |
| Eto et al, 2022 [43] | 9/15 anterior decompression and fusion  4/15 posterior decompression and fusion  2/15 laminoplasty | rs-fMRI at: baseline and 6 months after surgery | FC | **Post- vs Pre-Surgery DCM**  ↑ FC between R lateral portion of sensorimotor network and L precentral gyrus, L postcentral gyrus and L lateral portion of sensorimotor network, between L lateral portion of sensorimotor network and R central opercular cortex, between L inferior temporal gyrus and L superior parietal lobule  ↓ FC between L postcentral gyrus and L supramarginal gyrus of salience network |  |
| Hrabálek et al, 2018 [46] | 17/20 one-level anterior decompression  3/20 two-level anterior decompression | fMRI at: baseline, 6 months and 12 months after surgery | Activation during active flexion/extension of R and L wrists | **Abnormal vs Normal MEP DCM**  *Right wrist:* ↑ activation in contralateral sensorimotor cortex at 6 months and persisting at 12 months for  *Left wrist:* ↑ activation in contralateral sensorimotor cortex and cerebellum at 6 months and persisting at 12 months, with ↑ activation in ipsilateral sensorimotor cortex at 12 months (not present at 6 months)  *Normal MEP group:* activation ↓ in ipsilateral sensorimotor cortex for R at 6 months and L at 12 months. Activation ↑ in contralateral sensorimotor cortex at 12 months for R wrist.  *Abnormal MEP group:* activation ↑ in contralateral sensorimotor cortex at 6 months for R and L wrist. Activation ↓ for cerebellum in R at 6 months and L at 12 months. |  |
| Ryan et al, 2018 [47] | NR | fMRI at: baseline, 6 weeks and 6 months after surgery | VOA during finger tapping, BOLD signal | **6 Weeks Post-Surgery DCM vs HCs**  ↑ VOA in contralateral M1, contralateral SMA, ipsilateral SMA. Difference in change in VOA significant in contralateral PMC, contralateral SMA, ipsilateral PMC  ↓ % BOLD signal in contralateral SMA  **6 Months Post- vs Pre-Surgery DCM**  ↑ % BOLD signal of ipsilateral SMA  **6 Months vs 6 Weeks Post-Surgery DCM**  Difference in change in % BOLD signal significant in contralateral SMA |  |
| Sawada et al, 2018 [48] | 4/6 one-level anterior discectomy (anterior fusion)  2/6 two-level anterior discectomy and partial corpectomy (anterior fusion) | fMRI at: baseline and 7 days after surgery | Activation during finger tapping | **Post- vs Pre-Surgery DCM**  ↓ activation in SMA and ACC  No significant change in FC in thalamus |  |
| Aleksanderek, et al 2017 [49] | 12/15 of mild and 7/13 of moderate DCM 1-level decompression  3/15 of mild and 6/13 of moderate DCM 2-level decompression | fMRI and MR spectroscopy at: baseline and 6 months after surgery | VOA during finger tapping, metabolite concentrations | No significant change in activation between mild DCM, moderate DCM or HCs after surgery |  |
| Bhagavatula et al, 2016 [51] | 12/17 laminectomy  5/17 anterior decompression | fMRI at: baseline and 6 months after surgery | VOA during finger tapping | **Post- vs Pre-Surgery DCM**  ↓ VOA in ipsilateral primary sensory cortex, combined sensory and motor cortices  ↑ VOA in premotor and SMA (both were not activated in pre-surgery DCM at all)  **Post-Surgery DCM vs HCs**  ↑ VOA in contralateral primary sensory cortex, cerebellum, combined sensory and motor cortices, contralateral precentral gyrus, premotor and SMA (latter 2 were not activated in HCs at all) |  |
| Duggal et al, 2010 [52] | NR | fMRI at: baseline and 6 months after surgery | VOA during finger tapping | **Post- vs Pre-Surgery DCM**  ↑ VOA in sensorimotor cortex, primary motor cortex, primary sensory cortex. Post-surgery DCM had additional activation in BA1 (not present in pre-surgery).  **Post-Surgery DCM vs HCs**  ↓ VOA in sensorimotor cortex, primary sensory cortex  ↑ VOA in primary motor cortex |  |
| Dong et al, 2008 [53] | 3/8 anterior cervical discectomy and fusion  2/8 cervical laminoplasty and fusion  1/8 anterior corpectomy and fusion  1/8 laminoplasty  1/8 laminectomy | fMRI at: baseline, 3 months and 6 months after surgery | VOA and activation magnitude during wrist extension and 3-finger pinch | **3 Months Post-Surgery DCM vs HCs**  ↓ activation magnitude in contralateral dorsal premotor area during pinch  ↓ VOA in B anterior cingulate motor cortex, ipsilateral SMA, contralateral primary sensory cortex during wrist extension  **6 Months Post-Surgery DCM vs HCs**  ↓ activation magnitude in contralateral primary motor cortex during pinch  **6 vs 3 Months Post-Surgery DCM**  ↑ activation magnitude in contralateral dorsal premotor area during pinch task  Between-task differences in pre-surgery gradually disappeared after surgery, with exception of larger VOA in the ipsilateral cerebellum at 3 months post-surgery and higher activation magnitude in contralateral dorsal premotor area at 6 months post-surgery for pinch-task compared to wrist extension.  Linear ↑ in VOA in ipsilateral primary motor cortex and primary sensory cortex during pinch and ↓ in activation magnitude in contralateral SMA during wrist extension from pre-surgery to 3 months to 6 months post-surgery  Linear dorsal (z coordinate) shift in centre of ipsilateral dorsal premotor area activation during pinch. Linear lateral (x coordinate) shift in centre of bilateral primary sensory cortex activation during wrist extension. |  |
| Holly et al, 2007 [54] | 2/4 laminectomy and fusion  1/4 laminoplasty  1/4 anterior cervical discectomy and fusion | fMRI at: baseline and at least 2 of 6 weeks, 3 months or 6 months after surgery | Activation during ankle dorsiflexion or wrist extension | **Post-Surgery DCM cases vs HCs**  2 DCM patients demonstrated focussing patterns of cortical activation which incrementally ↓ from baseline and began to resemble HCs. Other 2 patients had shifting patterns of activation along with additional recruitment following by focussing of the activation at later time points.  *Case 1*: 6-weeks post-surgery had more restricted activation patterns to a smaller region of the contralateral primary motor and sensory areas and much smaller portions of the SMA and premotor cortex. This persisted 3 months after surgery.  *Case 2*: 3 months post-surgery had ↓ activation pattern size followed by significant focussing to a small region corresponding to the area of the wrist at 6 months.  *Case 3*: 6-weeks post-surgery had similar extent of activity but pattern was restricted entirely to contralateral medial motor cortex. At 3-months, activation had ↑ in primary motor cortex, mostly contralateral to foot with much more involvement of B SMA and dorsal premotor areas. At 6-months, there was a focussing pattern to primarily include contralateral ankle motor area. |  |
| Takenaka et al, 2020 [55] | 25/28 cervical laminoplasty  3/28 anterior decompression and fusion | rs-fMRI at: baseline and 6 months after surgery | ALFF | **Post- vs Pre-Surgery DCM**  ↓ ALFF in visual cortex, B precentral gyrus, R lingual gyrus, R occipital pole, L precentral gyrus. B primary sensorimotor cortex and L visual cortex overlapped with clusters showing increased ALFF in pre-surgical DCM patients.  ↑ ALFF in L frontal pole/B superior frontal gyrus/L paracingulate gyrus, L frontal pole, precuneus cortex, L temporal pole, L superior frontal gyrus, L supramarginal gyrus (posterior division), R temporal pole, R superior frontal gyrus, L supramarginal gyrus (posterior division) |  |
| Tan et al, 2015 [62] | NR | rs-fMRI at: baseline and 3 months after surgery | ReHo | **Post- vs Pre-Surgery DCM**  ↓ ReHo in R superior parietal lobule  ↑ ReHo in L postcentral gyrus, L precentral gyrus |  |

A total of 16 studies explored functional changes in 286 DCM patients after surgical intervention. All six of the studies that investigated FC identified significant changes [24, 31-33, 38, 43]. Takenaka *et al* (2019) only reported increased FC following surgery, including between the supracalcarine cortex and superior frontal gyrus, which was decreased in DCM patients prior to surgery [38]. FC recovery associated with the visual cortex [34] and SMN [43] were also reported. Two studies identified post-surgical FC changes associated with subcortical structures, including the cerebellum [24] and thalamus [24, 32]. Sawada *et al* (2020) observed decreased FC between the postcentral gyrus and dorsolateral prefrontal cortex following surgery in the poor recovery group [31]. Although seed-to-voxel analysis (explores FC of each unit of an anatomical atlas with seed regions of interest) revealed no significant change between poor and good recovery groups, seed-to-seed analysis (explores FC of seed regions of interest with each other) identified that the same FC between the postcentral gyrus and dorsolateral prefrontal cortex was significant only in the poor recovery group. This FC was later shown to be most effective at distinguishing poor recovery from good recovery patients [31].

All eight studies that examined activation/VOA reported changes in the SMN following surgery [46-49, 51-54], with multiple reporting the stabilisation of DCM patients towards HC measurements [48, 49, 51, 53, 54]. In a longitudinal study, Dong *et al* (2008) found that both finger pinch- and wrist extension-related activations in sensorimotor cortices became more similar to those of HCs after surgery [53]. Similarly, Holly *et al* (2007) found that DCM patients all eventually exhibited focussing patterns of activation within the SMN to more resemble HCs following surgery [54]. Bhagavatula *et al* (2016) found decreased VOAs in the sensorimotor cortices became more similar to those seen in HCs (despite still remaining increased following surgery) and that post-surgical patients had recruited two new regions, the premotor area and SMA [51]. Sawada *et al* (2018) reported diminished activation in the ACC and SMA, the latter being increased in pre-surgical patients [48]. On the other hand, Ryan *et al* (2018) reported that the overall stable pattern of VOAs in DCM patients from baseline to 6 months differed to HCs, who showed a temporal decline associated with motor learning [47]. Hrabálek et al (2018) reported persistent hyperactivation in the sensorimotor cortex of abnormal MEP DCM patients, even after surgery [46].

Takenaka *et al* (2020) reported on several changes in ALFF following surgery, including a decrease in the sensorimotor and visual cortices, which overlapped with clusters that showed increased ALFF in pre-surgical patients [55]. Ryan *et al* (2018) reported that % BOLD signal decreased in the contralateral SMA in 6 weeks post-surgical patients compared to HCs, but later increased in 6 months post-surgical patients compared to pre-surgical patients [47]. Tan *et al* (2015) reported a decrease in ReHo in the superior parietal lobule and an increase in ReHo in the pre- and post-central gyri following decompression [62]. These changes are the opposite to those measured in pre-surgical patients.

**Supplementary Data 10 – Post-Surgical ‘Other MRI’ Techniques**

**Supplementary Data 10. Summary of included studies utilising ‘other MRI’ techniques in patients with degenerative cervical myelopathy (DCM) after surgical intervention.** Missing information is reported as NR. Incorrectly reported information is reported as IR. Arrows indicate significantly increased (↑) or decreased (↓) changes.

*ADC = apparent diffusion coefficient; ALFF = amplitude of low-frequency fluctuations; ALS = amyotrophic lateral sclerosis; ASL = arterial spin labelling; CBF = cerebral blood flow; dALFF = dynamic ALFF; DCM = degenerative cervical myelopathy; DWI = diffusion-weighted imaging; EC = effective connectivity; FA = fractional anisotropy; FC = functional connectivity; FCS = FC strength; FLAIR = fluid attenuated inversion recovery; GFA = global FA; gFCD = global FC density; GMV = grey matter volume; HCs = healthy controls; MR Spec = MR spectroscopy; NQA = normalized quantitative anisotropy; ReHo = regional homogeneity; sALFF = static ALFF; SC = structural connectivity; VOA = volume of activation; WMV = white matter volume.*

| **Reference** | **Type of Decompression Surgery** | **Imaging Modality and Timing** | **Outcomes Assessed** | **Changes After Surgery** | |
| --- | --- | --- | --- | --- | --- |
| Aleksanderek et al, 2017 [26] | 19/28 1-level decompression  9/28 2-level decompression | MR spectroscopy at: baseline and 6 months after surgery | FA and mean diffusivity of white matter, metabolite concentrations | **Post- vs Pre-Surgery DCM**  ↓ NAA/Cr ratio  No significant change in Cr, Cho/Cr, Ins/Cr or Glu/Cr ratios over time |  |
| Aleksanderek, et al 2017 [49] | 12/15 of mild and 7/13 of moderate DCM 1-level decompression  3/15 of mild and 6/13 of moderate DCM 2-level decompression | MR spectroscopy at: baseline and 6 months after surgery | Metabolite concentrations | **Post-Surgery Mild DCM vs HCs**  ↓ NAA/Cr ratio  **Post- vs Pre-Surgery Mild DCM**  No significant change in NAA/Cr ratio  **Post-Surgery Moderate DCM vs HCs**  ↓ NAA/Cr ratio  **Post- vs Pre-Surgery Moderate DCM**  ↓ NAA/Cr ratio  **Post-Surgery Moderate vs Mild DCM**  NAA/Cr ratio in moderate DCM decreased such that mild and moderate DCM patients had similar NAA/Cr ratios. Both mild and moderate DCM patients had VOAs that did not differ significantly, shifted towards primary sensory cortex (L parietal lobule) |  |
| Goncalves et al, 2016 [63] | NR | MR spectroscopy at: baseline, 6 weeks and 6 months after surgery | Metabolite concentrations | **6 Weeks Post- vs Pre-Surgery DCM**  ↓ NAA in motor cortex  No significant change in NAA in sensory cortex  **6 Months vs 6 Weeks Post-Surgery DCM**  No significant change in NAA in motor cortex  ↓ NAA in sensory cortex  **6 Months Post- vs Pre-Surgery DCM**  ↓ NAA in motor cortex and sensory cortex  No significant change in other metabolites in motor or sensory cortices across all three time points or when comparing single- and multi-level disease |  |

### Three studies utilised other MRI techniques (MR spectroscopy and ASL) in 73 DCM patients after surgical intervention. All three of the studies that measured metabolite concentrations in DCM patients following surgery observed a decrease in NAA levels, suggesting that despite reported clinical improvement, metabolic dysfunction persists [26, 49, 63]. Interestingly, Goncalves *et al* (2016) reported a temporal difference whereby NAA decreased first in the primary motor cortex, then later in the primary sensory cortex [63].
